# Precision dietary strategies for enriching animal-derived foods with antioxidants and omega-3 fatty acids

**DOI:** 10.1016/j.vas.2026.100791

**Published:** 2026-07-29

**Authors:** Omeralfaroug Ali, Moses Teye, Tamás Tóth, George Bazar, András Szabó, Richard Badu, Mohamed Maki, Haruna Gado Yakubu

**Affiliations:** a Agribiotechnology and Precision Breeding for Food Security National Laboratory, Institute of Physiology and Animal Nutrition, Department of Animal Physiology and Health, Hungarian University of Agriculture and Life Sciences, Kaposvár, Hungary; b Department of Animal Science, School of Agriculture, College of Agriculture and Natural Sciences, University of Cape Coast, Cape Coast, Ghana; c Agricultural and Food Research Centre, Széchenyi István University, Győr, Hungary; d Adexgo Kft, Balatonfüred, Hungary; e Department of Biochemistry and Medical Chemistry, Medical School, University of Pécs, Pécs, Hungary

**Keywords:** Functional feed additives, Biofortification of animal products, Selenium, n-3 polyunsaturated fatty acids, Precision enrichment, Bioavailability, Antioxidant synergy, Rumen biohydrogenation

## Abstract

Functional food demand has driven research into dietary strategies that enhance the nutritional value of animal-derived foods. This narrative review (2010–2025; 225 studies) evaluates supplementation with selenium (Se), zinc (Zn), vitamin E (Vit E) and omega-3 polyunsaturated fatty acids (n-3 PUFAs) to enrich livestock-derived meat, milk and eggs, proposing a precision enrichment framework. Organic and some novel forms of Se and Zn generally show greater efficacy than inorganic sources in many studies, largely due to differences in bioavailability and metabolic handling. Potent synergies were identified between Se-dependent glutathione peroxidase and Vit E (extending antioxidant efficacy) and between plants (C18:3n3-rich) and marine oils (C20:5n3/C20:6n3-rich), yielding broad-spectrum lipid enrichment. Unlike monogastric animals, in ruminants, protected lipid forms are often more effective for improving transfer of unsaturated fatty acids into animal products by reducing ruminal biohydrogenation, although responses vary with lipid source, protection method, diet and species. However, high dietary unsaturation is positively associated with oxidative risk, generally warranting antioxidant co-supplementation to preserve product stability and shelf-life. Although some studies used inclusion levels above regulatory recommendations (EU/EFSA and FDA), comprehensive toxicological or environmental assessment were not conducted. Based on the available evidence, functional food development requires shifting from single-additive approaches to a "precision enrichment" framework integrating nutrient-specific deposition pathways, species-specific physiological barriers, and nutrient synergies/antagonisms. Effective biofortification is supported by current literature; however, to confirm commercial viability and environmental sustainability, future efforts should prioritize long-term toxicological assessments at high inclusion levels, rigorous human health intervention trials, and economic feasibility studies.

## Highlights

• Organic Se/Zn forms generally outperformed inorganic forms in most reviewed studies (attributed largely to higher bioavailability and, for Se, non-selective protein incorporation), though evidence heterogeneity exists.
• Rumen-protected lipids are generally advantageous in ruminants to reduce biohydrogenation losses, although partial enrichment has also been reported with some unprotected sources; monogastric animals need C20:5n3 (EPA)/C22:6n3 (DHA)-rich oils due to conversion limitations (low *FADS2*/Δ6-desaturase activity).
• Se-dependent glutathione peroxidase spares Vit E (α-tocopherol) by reducing lipid hydroperoxides; Vit C regenerates the tocopheroxyl radical.
• Antioxidant co-supplementation may need to be adjusted according to dietary PUFA load, with species- and product-specific validation.
• A precision nutrition framework integrating forms, deposition mechanisms, nutrient interactions (synergy/antagonism), and species-specific physiology (monogastrics vs ruminants) is proposed to optimize functional product development.

## Graphical abstract

**Figure:**
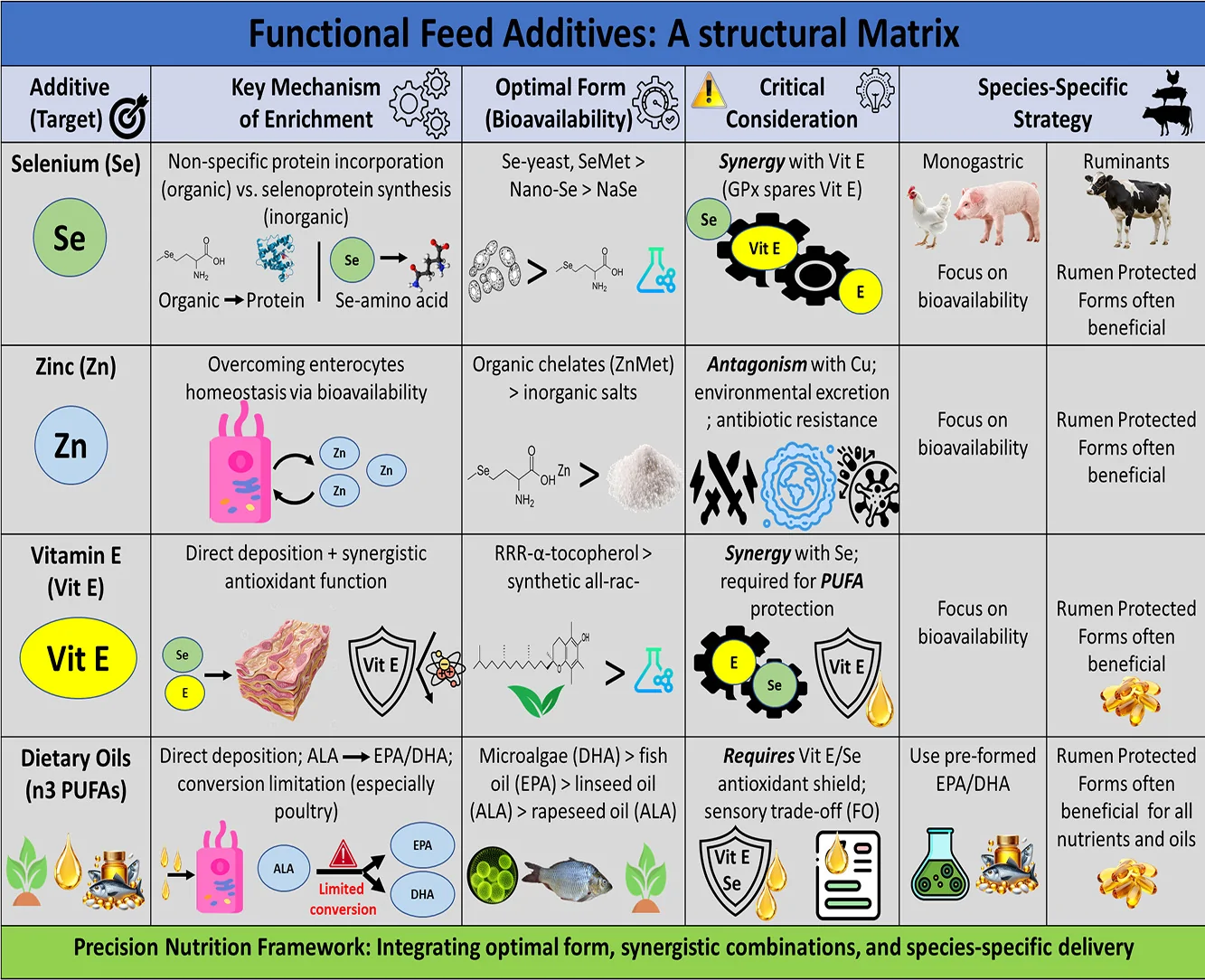

Summarizing feed additives and their precision enrichment framework for functional animal products.

Implications and importance of the review

What you eat comes from what animals eat. This review suggests that adding specific nutrients like selenium, zinc, vitamin E and healthy omega-3 fats to livestock feed can improve the nutrient profile of meat, eggs and milk, although direct disease-reduction benefits require confirmation through robust human intervention studies. We also introduce a practical decision framework to help farmers and nutritionists select appropriate nutrients, doses, and forms for each animal species, avoiding overuse that may waste resources and harm the environment. This review links animal nutrition with functional food development by integrating evidence across multiple nutrients and animal-derived products.

## 1 Introduction

***Animal-derived foods*** such as meat, milk, and eggs are among the main sources of high-quality protein for human growth and development (Mariotti, 2019). Their consumption also provides a valuable source of vitamins and minerals (Andreoli et al., 2021; Caffarelli et al., 2010). Health-conscious consumers, such as individuals diagnosed with cardiovascular disorders, are also interested in animal products with low to moderate fat contents and a favourable ratio of unsaturated fatty acids (UFAs), especially those with high omega-3 (n-3) FA levels (Liput et al., 2021). Consequently, one of the primary goals for animal nutritionists is to reduce the ratio of saturated FAs (SFAs) to polyunsaturated FAs (PUFAs) in these animal sources to help mitigate the diet-associated risk of diseases (Liput et al., 2021; Zhubi-Bakija et al., 2021). This risk-mitigation objective can be effectively achieved by strategies that simultaneously reduce SFA content and increase n-3 PUFA proportions, especially in a proportional relevancy to omega-6 (n-6), thereby improving the dietary n-6:n-3 ratio toward the recommended range of 4:1 or lower. However, caution is needed when such strategies are executed, as diets with a high unsaturation index are inherently susceptible to lipid peroxidation, a process that not only degrades diet quality during storage but can also compromise the antioxidant status, product quality, and shelf life of the derived animal foods. This vulnerability provides a mechanistic rationale for the co-supplementation of antioxidant micronutrients, especially with vitamin E (Vit E) and selenium (Se), alongside n-3 PUFA-enriching strategies, a central theme developed throughout this review. Overall, the potential biological value of enriched animal products is very broad, as they have shown preventive associations with chronic diseases like cancer, cardiovascular diseases, gastrointestinal tract disorders, and neurological conditions, as supported primarily by epidemiological and preclinical models (Alkhatib et al., 2017; Fekete et al., 2025; Galasso et al., 2019; Luvián-Morales et al., 2022; Zhang et al., 2025a); robust human intervention trials remain limited. However, most preclinical evidence is derived from animal models and, to a limited extent, from human subjects, underscoring the demand for robust clinical validation to consolidate public health recommendations and attain broader market acceptance. Without comprehensive human data, the full potential and credibility of these functional animal products remain constrained.

The concept of functional food dates back to the mid-1980s in Japan. Currently, several ***definitions*** exist; however, in common perceptions, functional food is commonly understood and defined as the food commodity that comprises at least one or more specific nutrient(s) or non-nutrient(s), which, upon consumption, may help promote consumer health and/or reduce the risk of chronic diseases, acting beyond the traditionally known nutritional effects (Garud et al., 2023). The Food and Agriculture Organization holds a similar definition, highlighting a role that extends beyond basic nutrition (https://www.fao.org), whereas the European Food Safety Authority (EFSA) definition focuses on beneficial effects on targeted bodily functions in a way beyond adequate nutritional effects (Duttaroy, 2019). In a practical sense, functional product production can be achieved through several schemes: (1) the addition of an element/component (e.g., minerals or probiotics), (2) the removal or reduction of a component (e.g., fatty acids, or FAs), (3) the modification of a component (e.g., hydrolysis), (4) the enhancement of bioavailability (e.g., enzymes), and (5) any combination thereof. In terms of feed additives, strategies and novelties within this industry serve diverse perspectives; for example, strengthening animal health and performance, enriching product quality, and advancing environmental sustainability. This philosophy highlights the entanglement and relevance of animal nutrition within the broader context of food systems and environmental footprints.

**Fig. 1:**
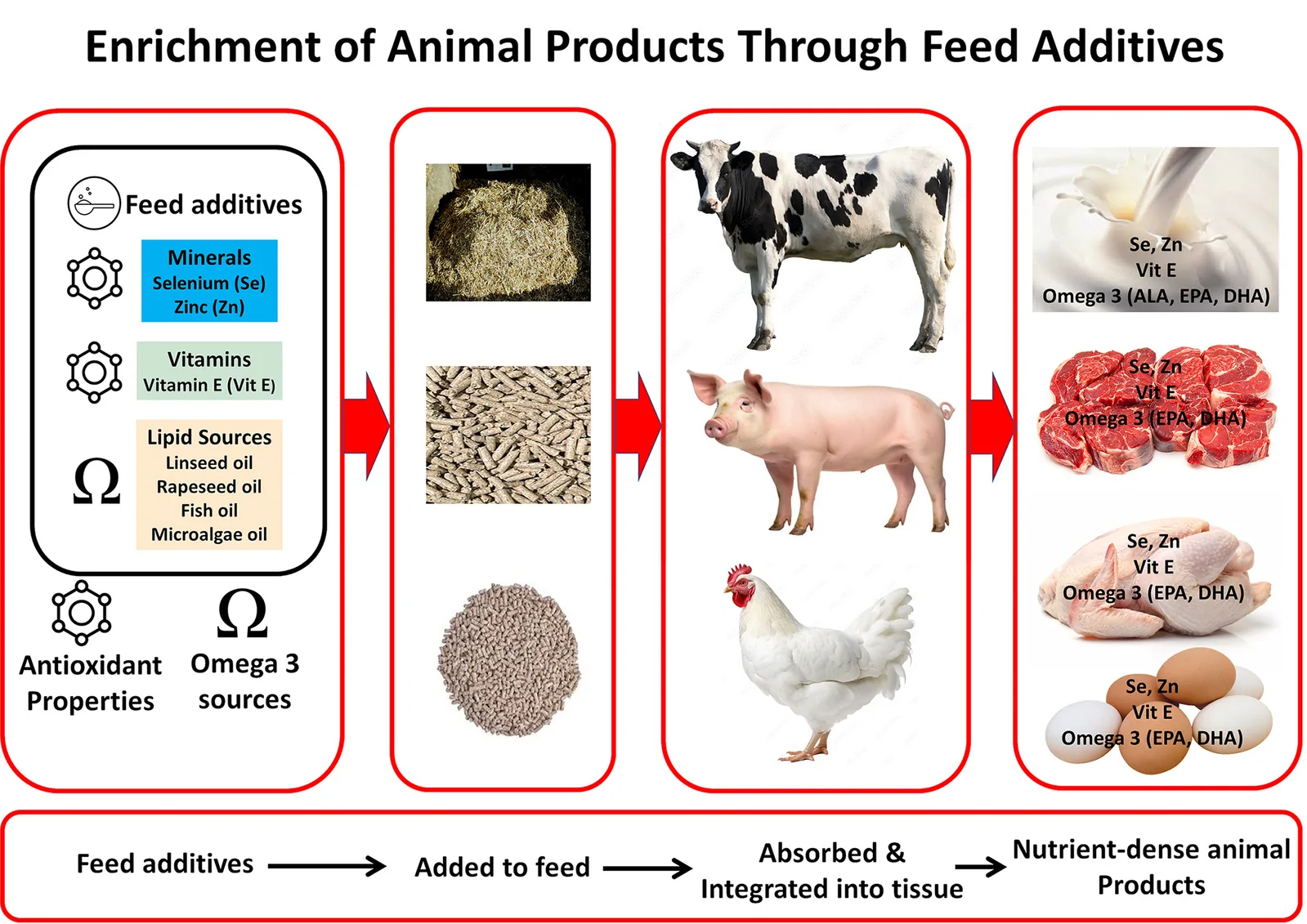
A diagram showing common additives and raw materials (inputs) used in feed supplementation to improve animal product quality (outputs).

The ***development of functional products*** has positively shifted consumer perception and demand, consequently prompting producers to prioritize their production toward highly functional products. This increasing demand for antioxidant-enriched meat, eggs, and milk has placed a premium on products such as Se-enriched meat (Li et al., 2024), α-tocopherol acetate (Vit E)-enriched eggs (Nemati et al., 2020), Zn-enriched meat (Huang et al., 2019), and n-3 PUFA-enriched products (Kolanowski & Laufenberg, 2006). This market shift is underpinned by efficient biological processes, wherein the animal’s body serves as a biological converter with efficient metabolic pathways (e.g., deposition, elongation and desaturation) capable of synthesizing or converting added or supplemented feed elements, such as sodium selenite (Na_2_SeO_3_, or NaSeIII) or selenate (Na_2_SeO_4_, or NaSeVI), Se-yeast, zinc oxide (ZnO), zinc sulfate (ZnSO_4_)_,_ and α-tocopherol acetate, into bioavailable forms for deposition in the final animal product, thereby enriching its value, as demonstrated in Fig. 1.

***Dietary manipulation strategies*** have shown considerable effectiveness in improving the antioxidant and essential element contents of meat, milk, and eggs, which is achieved through the inclusion of these supplements in animal diets at acceptable thresholds that have the propensity to enrich the final product (El-Sabrout et al., 2024). In this regard, feed additives are simply strategic dietary tools aimed at mitigating risks and/or improving quality; hence, their roles take into account enrichment, stabilization and risk management. These aspects facilitate the efficient use of diverse or low-quality feedstuffs, which is a pillar of economic resilience. Generally, common categories of feed additives include minerals, vitamins, FAs, enzymes, pro- and prebiotics, antioxidants, and, historically, antibiotic growth promoters (AGPs); however, we deliberately ***focus on nutritional strategies*** involving trace minerals like Se and Zn, the antioxidant Vit E, and dietary oils for the following reasons: (1) some are directly deposited into animal tissues and, consequently, their obtained products. (2) their reported efficacy (whether through direct accumulation or upon hydrolysis and metabolism) in enriching animal products (meat, milk and eggs). (3) their extensive use in the diets of livestock animals, and (4) their market availability and affordability. Thus, within the context of creating functional animal products and nutritional practices, this review highlights the following key additive types: key nutrients associated with the integrated antioxidant system (i.e., Se, Zn, and Vit E, which are found in both inorganic and organic forms (possibly natural and/or synthetic), and dietary oils serving as essential FA sources (i.e., microalgae, fish, linseed, and rapeseed oils that are rich in n-3 PUFAs).

When the literature has been explored, most existing reviews have focused on the effects of a single additive on a particular animal product; such an approach inherently narrows both the mechanistic understanding and the practical applicability of dietary manipulation strategies. In contrast, this review provides a ***comprehensive synthesis*** (including multiple livestock species), highlights key synergies and antagonisms, discusses limitations in the literature, and identifies future priorities related to the functional effects of different antioxidants (Se, Zn, and Vit E) and dietary oils across various animal products (meat, milk and eggs). Specifically, this review aims to: (i) outline and critically evaluate dietary manipulation strategies used to improve the nutritional and functional quality of animal-derived foods over the past fifteen years (2010–2025); (ii) identify species-specific physiological constraints and dietary interactions that govern enrichment efficacy; and (iii) propose a coherent framework of precision enrichment that integrates these biological determinants to guide future research and practical feed formulation.

## 2 Methodology or conceptual framework of the review

**Fig. 2:**
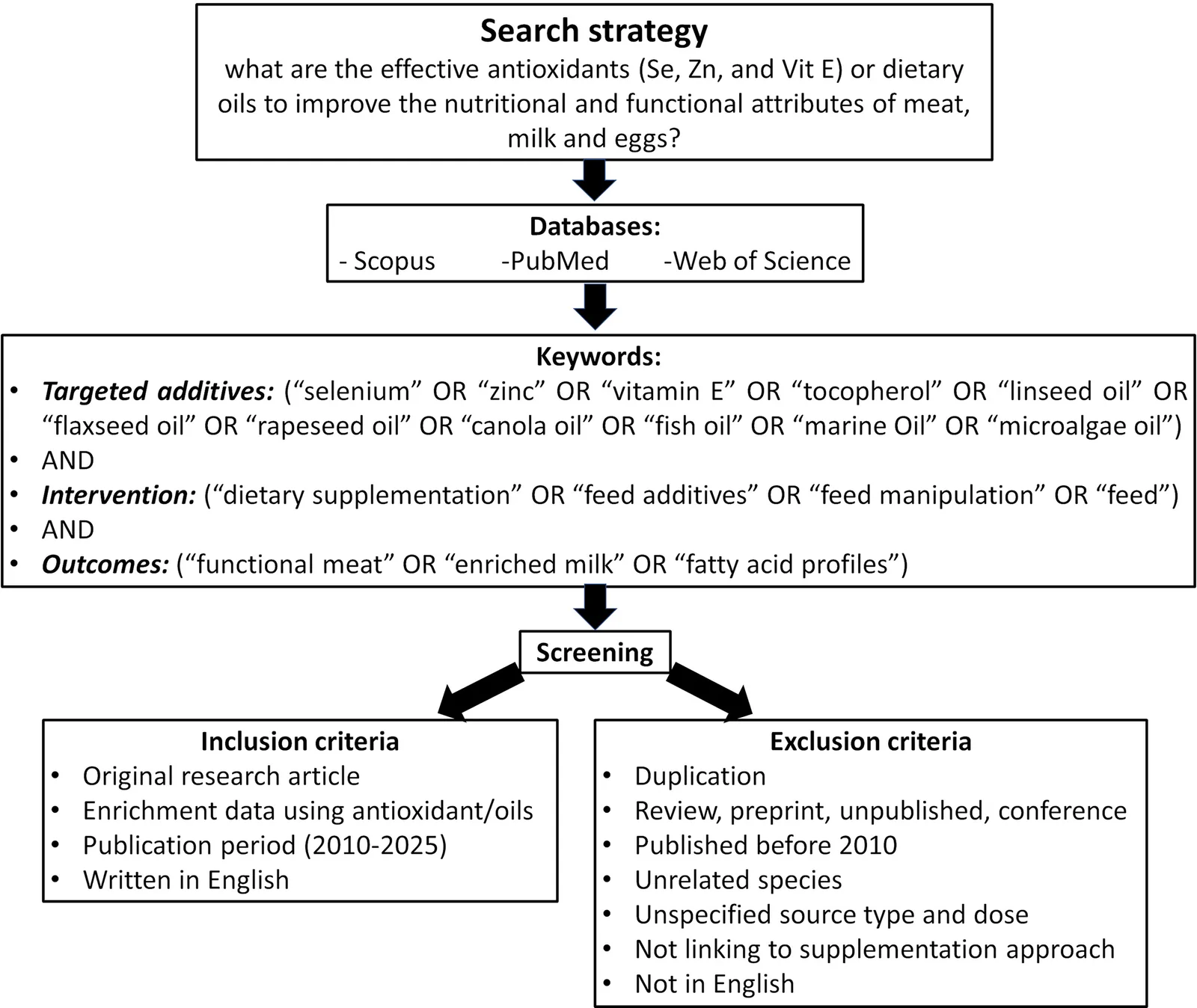
A diagram showing the search, screening, and selection strategies of the reviewed literature.

This structured narrative review was conducted to identify, select, and critically arrange relevant research on the topic, as summarized in Fig. 2.

### 2.1 Search strategy

**Fig. 3:**
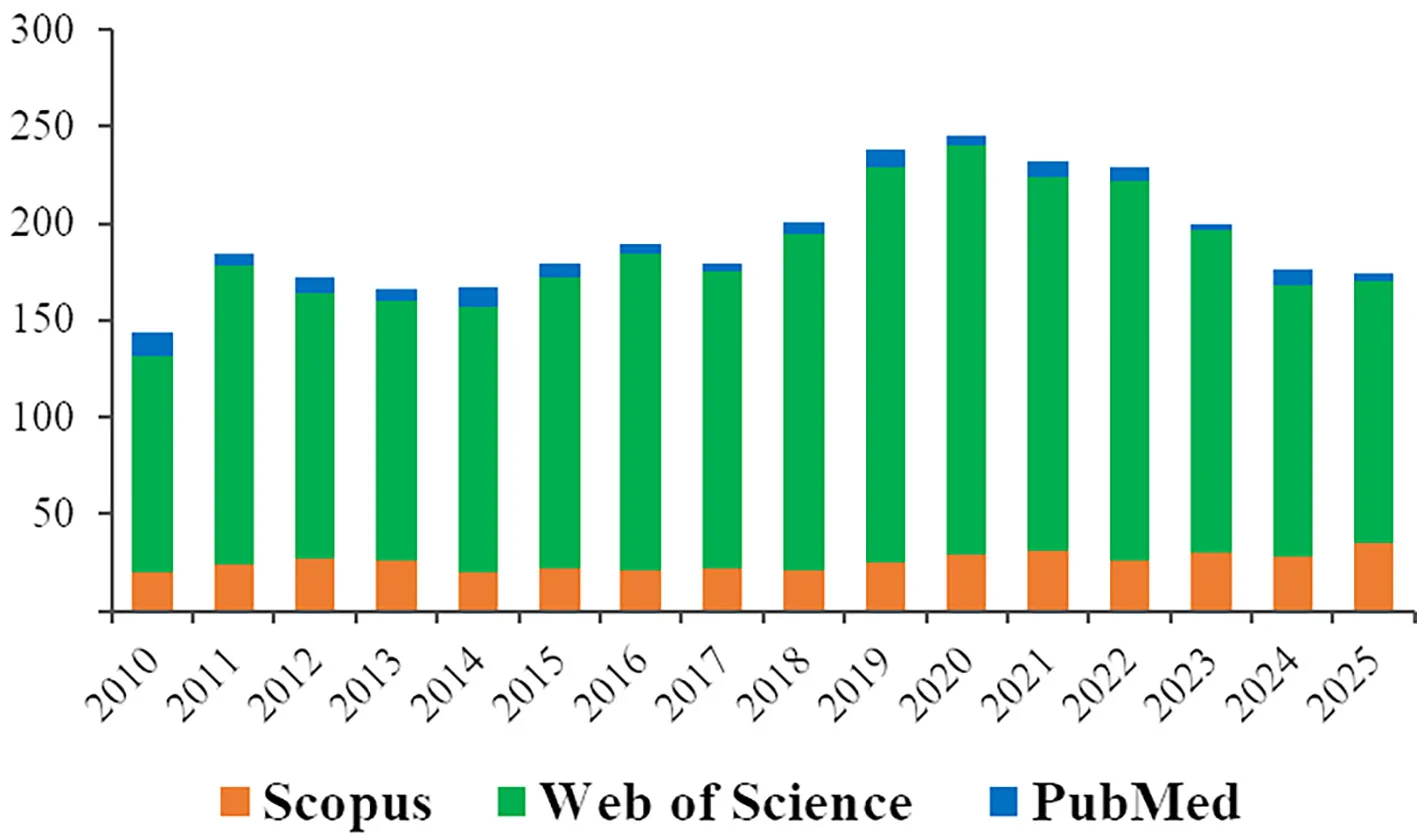
Recorded annual accumulation of articles in the researched databases, on the basis of key search terms. The chart shows the increasing trend (translated into annual publication numbers) in the review topics every 5 years during the investigated period.

The systematic search strategy for scientific or peer-reviewed publications was performed between January 1, 2010 and December 31, 2025, using the following databases: Scopus, PubMed and Web of Science. The search strategy was performed in a way to address the following core questions: “What are the effective antioxidants (Se, Zn, and Vit E) or dietary oils that improve the nutritional and functional attributes of meat, milk and eggs? During the search, a combination of keywords related to feed additives, target animals, and animal products was employed with the help of Boolean operators (“AND” and “OR”) to create a comprehensive search string. These key search terms included the following: (1) targeted additives: (“selenium” OR “zinc” OR “vitamin E” OR “tocopherol” OR “linseed oil” OR “flaxseed oil” OR “rapeseed oil” OR “canola oil” OR “fish oil” OR “marine oil” OR “microalgae oil”) AND (2) intervention: (“dietary supplementation” OR “feed additives” OR “feed manipulation” OR “feeding”) AND (3) outcomes: (“functional meat” OR “enriched milk” OR “enriched egg” OR “fatty acid profiles” OR “fatty acid composition”) AND (4) species: (“bovine” OR “cow” OR “beef” OR “dairy cow” OR “sheep” OR “lamb” OR “ewe” OR “goat” OR “poultry” OR “chicken” OR “broiler” OR “layer hen” OR “turkey” OR “duck” OR “goose” OR “rabbit”). A critical component of the synthesis was the comparative analysis of the efficacy of different nutrient forms and sources. The recorded article numbers from each database can be seen in Fig. 3.

### 2.2 Inclusion and exclusion criteria

Overall, studies were selected on the basis of the following criteria: (a) Original research article. (b) Investigating the nutrient enrichment in meat, milk and eggs through dietary antioxidants (Se, Zn and Vit E) and/or oils (linseed, rapeseed, fish and microalgae oils) supplementation. (c) Publications that fall within the time frame January 2010 to December 2025; however, in a few instances, earlier studies before the selected period were used in the comparison discussion. (d) Publications that were written and published in the English language. Further eligibility criteria included the following: (1) populations: swine, ruminants and poultry; (2) intervention: dietary supplementation with clearly defined levels of additives; (3) comparator: control diet free of additive(s) or with different forms or levels of the same additive; and (4) outcome: determination of nutritional changes (importantly, the quantitative determination of Se, Zn, Vit E (α-tocopherol), or FA concentrations in meat, milk, or eggs) in animal products. In the absence of an indexed report, non-indexed research articles were included. The exclusion criteria were as follows: (a) Unpublished manuscripts, non-peer reviewed manuscripts, pre-prints, conference abstracts, reviews and meta-analysis papers. (b) Studies published before 2010. (c) Studies not written in the English language. (d) studies did not directly link nutrient enrichment to dietary supplementation. (e) Studies for which the full text was unavailable. (f) Where multiple studies with near-identical designs and outcomes were identified, the most recent, highest-quality, or highest-impact study was selected as representative, with the selection rationale documented.

### 2.3 Data extraction and synthesis

Given the broad, multiadaptive scope of this review, a narrative synthesis was deemed most appropriate, wherein the collected records were synthesized narratively by categorizing reports on the supplemented additive used, the targeted animal product, and the animal product (like a subcategory). Within this process, all identified records were screened by three reviewers on the basis of the title and abstract, followed by full-text assessment for eligible studies. As an initial database search, approx. 3076 records were yielded. Upon removing 430 duplicate records, a total of 2645 records were screened on the basis of title and abstract levels, in which 2254 records were excluded as they did not meet the criteria, such as wrong species, outcomes not adequately described, review articles, … etc. The remaining 391 records were assessed for eligibility, through which 166 records were excluded for reasons like unavailable full text, undefined source or type, not being directly linked to the considered supplementation approach, non-English or duplicate reporting of the same dataset, ultimately leading to 225 original research studies included in the qualitative synthesis. Of note, 2 studies published in early 2026 (Hazarika et al., 2026; Salahi Kojur et al., 2026) were identified through supplementary hand-searching after the formal database search had concluded. These studies were used solely as contextual references to discuss recent trends and were not included among the 225 studies forming the qualitative evidence synthesis. Other studies, not among the 225 records, were also used to provide a comprehensive discussion, notably on distinct topics yet relevant to enrichment and the development of a precision enrichment framework. Any disagreements between reviewers were resolved through structured discussion or by the involvement of another reviewer. The extracted data were homogenized into a standardized form that included the type of dietary supplement used, its form and inclusion level, the farm animal species, the investigated animal product(s) (i.e., meat, eggs, or milk), and the primary outcomes (changes in the Se, Zn, Vit E and FA contents within the tissue/products). Upon collection establishment, a secondary sub-selection was applied, in which, when a large number of studies were available for a specific subcategory, those studies (across each additive and animal product category) with the most relevant key findings, dose-response thresholds, or mechanistic contrasts were selectively discussed in the text to illustrate broader trends as well as to avoid lengthy text. In addition, comprehensive summaries of essential information are presented in tables to enable easy understanding of dietary manipulation strategies across different studies.

Statistical meta-analysis was inappropriate due to the variation in experimental designs, animal species, Se/Zn/Vit E forms, oil sources, inclusion levels, and outcome measures across the included research. As a result, the results are narratively summarized and qualitatively evaluated for consistency, dose-response relationships, and effects specific to species and forms. Hence, the qualitative synthesis was structured deductively around 3 predetermined themes derived from the scoping objective: (1) form-dependent bioavailability (comparing inorganic, organic, and nano-forms); (2) species-specific physiological constraints (monogastric vs. ruminant digestion); and (3) nutrient interactions (synergies and antagonisms). Within each theme, data were synthesized narratively to identify consistent directional trends, dose-response thresholds, and mechanistic explanations for observed variances. Finally, charts that summarize key data make it simple to compare dietary manipulation techniques across studies.

## 3 Dietary strategies in animal nutrition that increase the levels of antioxidants and fatty acids

### 3.1 Selenium (Se)

Selenium (Se) is a nonmetal trace element (despite that earlier literature categorized Se as a metalloid, the current IUPAC convention (revised 2021) categorizes Se among the nonmetals) that is one of the most often used antioxidant micronutrients in animal feed formulation and food fortification (Kieliszek & Błażejak, 2016). As a cofactor for antioxidant enzymes like glutathione peroxidase and thioredoxin reductase, Se helps protect cells from damage to their membranes and reduces oxidative stress. It is also vital for animal physiology and metabolism, as it regulates thyroid function (Surai, 2006), the immune system (Hoffmann & Berry, 2008), fertility (Mistry & Kurlak, 2015), and overall health (Pecoraro et al., 2022). Furthermore, its activity in the muscles of animals can enhance the oxidative stability of meat (Chen et al., 2023; Giro et al., 2021); however, its deficiency can have severe health implications, including, but not limited to, muscle stiffness, tremors, motor disturbances, hind-end paralysis, and heart failure, particularly in young livestock (3-8 weeks of age). Common diseases associated with Se deficiency in livestock include white muscle disease (nutritional muscular dystrophy), retained placenta, ill-thrift, osteoporosis, and mastitis (Surai, 2006), all of which contribute to significant economic losses. Se has, therefore, been extensively used in animal production for its beneficial health effects and for producing functional foods such as Se-enriched meats, eggs, and milk (Kieliszek & Błażejak, 2016). In this context, adequate dietary Se intake has been associated with thyroid and cognitive function in observational and preclinical studies (Genchi et al., 2023), although causal relationships and optimal intake ranges for consumers of enriched animal products remain to be established through controlled human intervention trials. However, while Se has health benefits, excessive Se intake (above 400 µg/day) can be harmful to humans, indicating potential Se toxicity (Genchi et al., 2023; Vinceti et al., 2018). Such a conclusion is likely driven partly from animal models, which are prone to Se toxicity. Herein, the recent legislation has established a 0.2–0.5 mg/kg diet as the maximum total Se limit (Bampidis et al., 2024; Food and Drug Administration (FDA), 2025), depending on its form (organic *vs.* inorganic).

The ability to raise human Se levels through the oral ingestion of fortified animal-based foods exemplifies a direct and scalable public health approach, especially for regions where soil Se deficits lead to insufficient dietary intake. Hence, the manipulation of the Se content in eggs, milk and meat illustrates the direct application of animal nutrition research to address pervasive human public health issues, extending its impact beyond just animal performance to broader societal health improvements.

#### 3.1.1 Selenium forms: bioavailability and metabolic fate

**Fig. 4:**
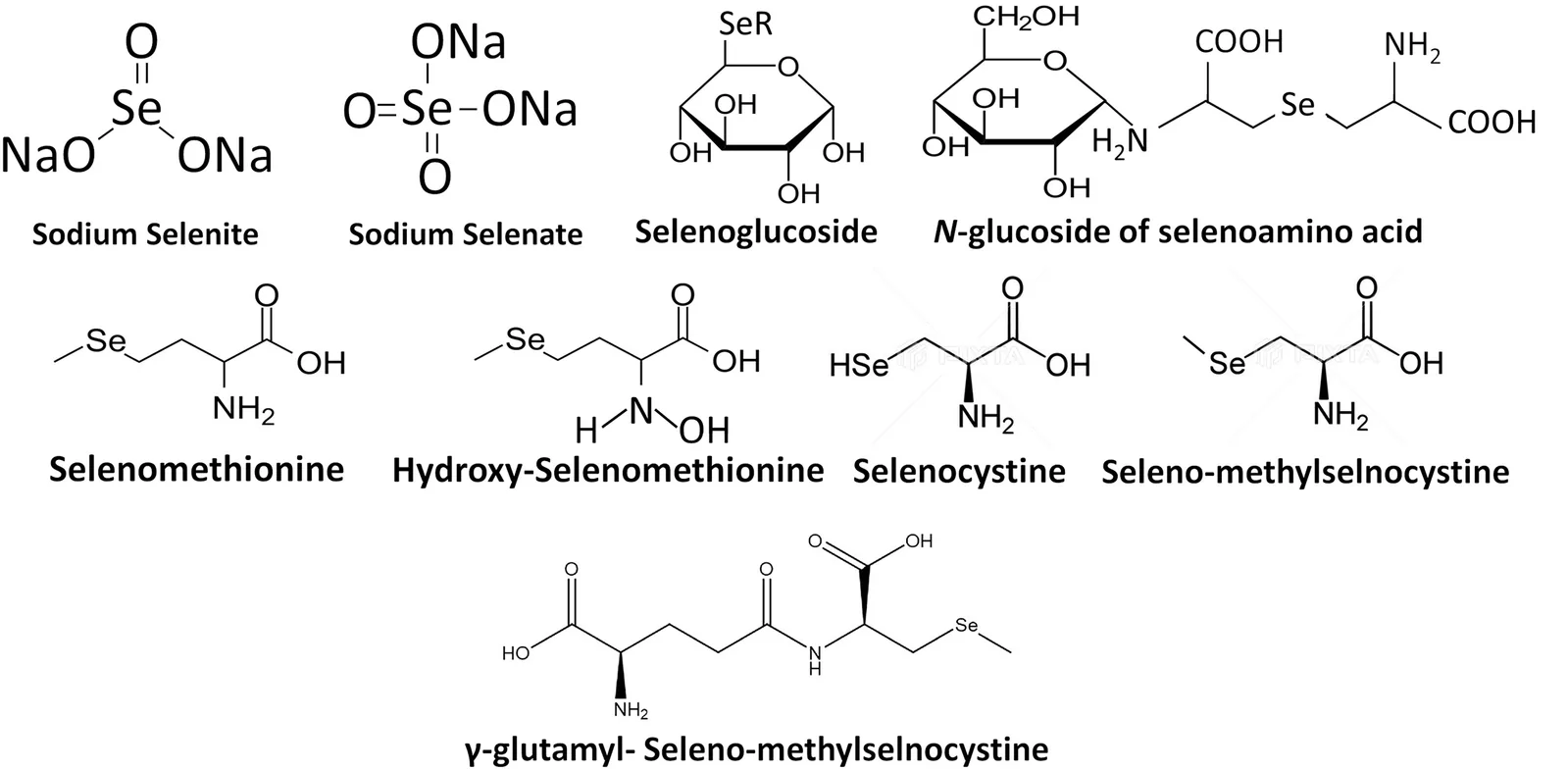
The structures of the different dietary selenium compounds mentioned in this review.

Naturally, selenium typically exists in four oxidative states, i.e., element (Se), selenide (Se^2-^), selenite (Se^4+^) and selenate (Se^6+^), while organically, it is available in gaseous (dimethylselenide, dimethyldiselenide) and nongaseous (bound to amino acids) forms (Mechora, 2019). At the biological level, selenate (Se^6+^) is mainly absorbed via the Na^+^-dependent sulfate co-transporter (NaSi-1/SLC13A1), which is expressed predominantly in the proximal small intestine (duodenum and jejunum), in a process that is rapid but subject to competition with dietary sulfate. On the other hand, selenite (Se^4+^) absorption occurs mainly in the duodenum and proximal jejunum through passive diffusion and potentially anion exchange mechanisms; unlike selenate, it is not a substrate for NaSi-1 and its absorption efficiency is generally lower and more variable depending on luminal redox conditions and the presence of thiols (e.g., glutathione), which can reduce selenite to selenide prior to absorption (Nickel et al., 2009; Vendeland et al., 1994, 1995). Notably, both selenate and selenite are ultimately reduced into selenide (Se^2-^) as the common intermediate en route to selenoprotein synthesis. Nutritionally, the most common ***sources*** of Se for feed supplementation/fortification include inorganic forms like NaSeIII and NaSeVI (EFSA, 2015), and organic forms such as selenoglucoside (SeGlu) (Zhou et al., 2020), Se-enriched *Cardamine violifolia* (SeCv, which is a Se-hyperaccumulating crucifer whose predominant Se species include Se-methylselenocysteine and γ-glutamyl-Se-methylselenocysteine, with minor contributions from selenomethionine and selenate (Zhao et al., 2022)), selenomethionine (SeMet, which includes various forms like hydroxyl-SeMet (OH-SeMet)), selenocysteine (SeCys), Se-yeast (containing diverse forms of selenoamino acids) (Rayman, 2020), seleno-methylselenocysteine (MSeCys), and γ-glutamyl-seleno-methylselenocysteine (Glu-SeMetCys) (Briens et al., 2014; Khanam & Platel, 2016); see Fig. 4. Considerably, in recent years, neither MSeCys nor Glu-SeMetCys has been well investigated for use in functional foods, representing a priority for further investigations. On the other hand, Se-enriched probiotics, in the form of Se-enriched bacteria, have been commonly used in feed supplementation strategies. These bacteria can utilize inorganic Se sources for cellular integration, subsequently converting them into organic forms, such as selenoproteins (Mohamed et al., 2020). In addition, the progress achieved in the field of nanotechnology has led to the utilization of Se nanoparticles (Nano-Se), which are encapsulated forms (often made up of lipids and polymers, protective structures) that preliminary evidence suggests may exhibit greater solubility and bioavailability than organic and inorganic forms in feed supplementation (Dawood et al., 2021; Nabi et al., 2020; Zhang, 2012), though large-scale, long-term validation in commercial settings is still required.

A marked finding in this field is the ease with which the Se content in these products can be manipulated through dietary interventions. However, an examination of earlier and recent literature (3.1.2, 3.1.3) revealed that Se accumulation profoundly ***varies*** depending on the Se form***,*** animal species, tissue type and diet composition. The form/chemical structure likely plays a noteworthy role in influencing bioavailability and metabolic processes, wherein inorganic sources are specifically incorporated (the active path) into selenoproteins (e.g., glutathione peroxidase) through the dedicated UGA-recoding machinery (selenocysteine insertion sequence (SECIS) element and dedicated elongation factor EFSec/EEFSEC), and this process undergoes strict homeostatic control whereby excess Se is excreted as urinary trimethylselenonium. Organic sources (e.g., SeMet, which is obtained from Se-yeast) are typically absorbed through intestinal amino acid transporters (more specifically, the neutral amino acid transporter B⁰/SLC1A5) and non-selectively bound to unintended sites on proteins or peptides (Fairweather-Tait et al., 2010; Labunskyy et al., 2014); hence, they are incorporated into general body bulk protein wherever methionine codons are translated, accordingly creating a reservoir and enriching animal protein products. Thus, the non-selective incorporation of dietary organic Se forms helps explain their high bioavailability rates, given that the reservoir is not immediately subject to homeostatic excretion; rather, it is mobilized over time. In addition, this organic form’s high bioavailability can be attributed to its high solubility and incorporation rate, as well as its longer retention period in the body, indicating efficient utilization (Fairweather-Tait et al., 2010). Therefore, organic Se can be referred to as a direct contributor to tissue/product enrichment, indexing its generally greater capacity to elevate Se levels in tissue/products compared to inorganic Se forms. Typically, organic Se sources reflect 60–95% bioavailability (Briens et al., 2014; Mahan et al., 2014; Surai & Fisinin, 2016; Takahashi et al., 2017), while inorganic Se forms reflect 30–60% (Juniper et al., 2008). Organic Se sources (e.g., Se-enriched plants (like wheatgrass), SeMet and Se-yeast) demonstrate comparable or superior bio-accessibility and tissue retention compared to inorganic forms (e.g., NaSe, including III and VI), with supplementation of organic sources elevating Se levels in animal tissues/products (e.g., broiler breast muscle and growing pig muscle) 2.5–3-fold more efficiently (Briens et al., 2014; Cun et al., 2015; Mahan et al., 2014). It is important to note that Se content varies across tissues, in which the highest deposition occurs in kidney and liver tissues, and notable storage also takes place in muscle.

#### 3.1.2 Impact of dietary selenium on animal products

The following sections provide evidence that the strategic choice of Se form and dose is effective for enriching animal products, with the organic forms consistently outperforming inorganic selenite due to the mechanism of non-specific incorporation.

##### 3.1.2.1 Effect of dietary selenium on the meat selenium content

Across the majority of reviewed studies in both monogastric and ruminant species, organic selenium sources (Se-yeast, SeMet, and OH-SeMet) resulted in greater Se deposition in muscle tissue than inorganic NaSe; however, some studies reported meaningful enrichment with inorganic forms (e.g., Aghwan et al. (2016) for NaSeIII in goat meat). This outperforming pattern of organic forms is mainly attributed to the non-specific incorporation of Se into body proteins, a mechanism extensively discussed in Section 3.1.1. For example, according to Silva et al. (2019), 0.45 mg of SeMet in ***pigs’*** finisher diets optimized Se deposition in muscles (which then confers antioxidant properties), whereas equivalent or higher doses of NaSeIII (0.3 and 0.6 mg/kg) targeted Se accumulation in the liver (P ≤ 0.05). In addition, Jiang et al. (2017) showed that organic Se-yeast, at 0.3 mg/kg feed, not only increased Se concentration (54% more than inorganic Se) in muscle but also drastically improved water-holding capacity of pork (by 58–74%) when it was supplemented with 1.5% linseed oil/kg diet, marking the synergism between Vit E and oils rich in PUFAs beneficial concerning the water-holding capacity. Complementing these findings, similar conclusions have been reported, alongside the lack of effect on meat qualities, as seen the study of Kawecka et al. (2013): 0.2 mg Se-yeast/kg with or without Vit E supplementation. A further study on pigs by Zabashta et al. (2018) demonstrated the benefit of integrating probiotics in Se-enriching strategy, revealing NaSeIII+ iodine+ probiotics (0.35 mg NaSeIII+ 0.2 mg I/kg feed) resulted in more efficient Se and I accumulation in liver, heart, and pork meat compared to minerals without probiotics. In a similar manner to pigs, fattening cows benefit from organic Se sources more than inorganic forms. For example, in ***beef*** production, specifically young Charolais bulls and Nellore cattle, Se-yeast supplementation demonstrated higher deposition efficacy (P ≤ 0.01) in *longissimus thoracis* compared to inorganic sources like NaSeIII at identical or varying (0.3 and 0.47 mg/kg DM) inclusion levels (Cozzi et al., 2011; Silva et al., 2020). This efficacy is likely to increase with Vit E co-supplementation, as demonstrated by Corrêa et al. (2021): 2.5 mg Se/kg DM+ 500 IU Vit E/kg DM). The stable Se form, OH-SeMet, has also been investigated, showing Se deposition efficacy in lamb meat compared to the control without Se supplementation (Bezerra et al., 2020): 0.5 mg OH-SeMet/kg feed. However, the superior efficacy of organic Se sources does not entirely neglect the potential role of NaSe in ruminants, which has been acknowledged in goats. Aghwan et al. (2016) reported that 0.6 mg NaSeIII/kg DM significantly elevated Se content (P ≤ 0.05) in the chevon goat *longissimus lumborum* muscle, suggesting that this inorganic form feeding strategy can still serve as a tool to produce Se-enriched meat, albeit potentially less effective compared to organic forms. Furthermore, SeVI has also demonstrated an additional distinct role by modulating the lipid profile, wherein its combination with linseed oil in lambs’ diets significantly upregulated ∆9-desaturation and, consequently, increased *cis*-9, *trans*-11 conjugated linoleic acid (CLA) isomers in adipose tissue and muscle, meanwhile decreasing FA catabolism (Czauderna et al., 2012).

Numerous other studies have also reported the superiority of organic Se forms over inorganic sources, a pattern that has also been documented in ***poultry*** muscle/meat, liver, and heart. For instance, SeMet supplementation markedly increased Se deposition (P ≤ 0.01) compared to NaSeIII (Wang et al., 2011; Yuan et al., 2011). Furthermore, SeMet in broiler diets has been reported to decrease health risk indexes and promote beneficial health attributes (Del Puerto et al., 2017), showcasing a dual nutritional benefit. Other organic Se sources also demonstrated Se deposition efficacy in broilers, even beyond inorganic Se. As an illustration, Liu et al. (2020) found 111–394% relative bioavailability in broiler breast from Se-yeast (0–0.4 mg/kg feed) compared to dietary NaSeIII (P ≤ 0.05). In addition, a quadratic Se deposition pattern (rather than linear) in broiler tissues was observed across Se-yeast inclusion levels of 0.1, 0.2, and 0.4 mg/kg feed (Liu et al., 2023a). Notably, Phyto-biotic addition was reported to further enhance the effect of dietary Se-yeast on oxidative stability and Se absorption (Konkol et al., 2021). At the comparative level across multiple Se forms, 0.5 mg of SeMet or Se-yeast/kg diet of yellow-feathered broiler diets elevated Se retention in breast muscle and boosted antioxidant capacity compared to NaSeIII, with SeMet uniquely enhanced meat redness (P ≤ 0.05), pointing to Se form-specific effects on muscle quality beyond mere deposition (Chen et al., 2024a). likewise, dietary bacterial selenoprotein (e.g., *Enterobacter cloacae*) exhibited higher deposition efficacy compared to NaSeIII (Mohamed et al., 2020); however, this efficacy was most likely strain-dependent, as Se deposition from *Klebsiella pneumoniae* and *Stenotrophomonas maltophilia* did not significantly differ (P ≤ 0.05). Moreover, both hydroxy selenomethionine (OH-SeMet) and Nano-Se emerged as effective additives in the limited studies available. OH-SeMet linearly increased breast muscle Se concentration (P ≤ 0.001) (Tang et al., 2021), whereas dietary Nano-Se, SeMet or Se-yeast supplementation at 0.3-0.5 mg/kg feed remarkably exceeded the deposition efficacy of dietary NaSeIII in the breast and thigh muscles (P ≤ 0.05), with Nano-Se exhibiting a deposition profile similar to Se-yeast, and preliminary evidence showing a lower toxicity risk at these elevated tissue concentrations (Bakhshalinejad et al., 2019; Bień et al., 2022; Khajeh Bami et al., 2022; Liu et al., 2015a). A further organic Se source, namely Se-enriched *Cardamine violifolia* (a Se-rich plant), also increased broiler chicken breast Se content by 14.4–127% at 0.2 and 0.5 mg Se/kg feed (Zhao et al., 2022), depicting a lower efficacy compared to Se-yeast (Liu et al., 2020). Generally, the strong efficacy pattern of organic source has been further extended to goose by Łukaszewicz et al. (2016) and Nemati et al. (2021), who found that Se-yeast significantly increased Se deposition and antioxidant capacity in the goose meat and liver, thereby extending meat shelf life. A similar superiority finding was demonstrated in quail meat using 0.2 mg SeMet or Se-enriched kale sprouts/kg feed (Chantiratikul et al., 2021), highlighting that even plant-based organic Se carriers can serve as effective enrichment tools. Quails generally appeared to benefit from dietary Se-yeast, with higher inclusion rates (1.5–3.5 mg/kg feed) increased Se not only in muscle and liver but also in kidney and heart (Islam et al., 2024). Se-conjugated protein from the diet of black soldier fly larvae is another novel organic form of Se that is noteworthy. According to Kurniawan et al. (2025), when this Se-conjugated protein was fed to broiler ducks at a rate of 5%, the level of monounsaturated FAs in meat increased without negatively impacting carcass characteristics; however, higher inclusion rates (7.5%) decreased slaughter weight and carcass yield, demonstrating the need for cautious scaling of the source and dose of novel Se conjugates.

Of note, the Se-induced antioxidant capacity is limited under high dietary PUFA supplementation, although this is controversial across studies, depending on the animal species and the specific chemical form of Se (Danuta et al., 2020; Rozbicka-Wieczorek et al., 2014; Yin et al., 2025), with the majority of obtained outcomes suggesting inorganic Se to exhibit pro-oxidant behaviour when co-supplemented with PUFAs, as compared to Se-yeast. Corroborating this concern, in broilers, Nano-Se and organic Se forms enriched oxidative stability and maintained the PUFA profile compared to inorganic sources (Giamouri et al., 2023), further underlining the inorganic Se's form-dependent oxidative risk in PUFA-rich matrices. The positive interaction between organic Se and PUFA has likewise been documented in the broiler and rabbit models, where Se-yeast levels (0.1–2.5 mg/kg feed) elevated the muscle Se content in a dose-dependent manner as well as favourably modulated the FA composition by increasing PUFA levels (Nyquist et al., 2013; Papadomichelakis et al., 2017), suggesting a dual enrichment benefit of organic Se beyond simple mineral deposition. Notably, for rabbits, 0.5 mg/kg identified as optimal for oxidative stability (Papadomichelakis et al., 2017); however, a relatively lower Se-yeast inclusion level (0.45 mg/kg diet) produced a 2-fold increase in Se in hind leg and Longissimus muscles (Matics et al., 2017).

In ***summary***, Se-yeast, SeMet and Nano-Se appear to be the most effective additives for enrichment strategies, with 0.2-0.5 mg/kg (for organic and inorganic forms, respectively) demonstrating an optimal balance between muscle enrichment and regulatory adherence (namely, European Union jurisdictions). Despite the high inclusion rates of organic Se (> 0.3 mg/kg feed), which enrich the Se content of meat, numerous concerns exist regarding potential toxicity, especially with long-term supplementation. Moving forward, further large-scale assessments are important to validate the safety and cost-effectiveness of novel forms, as well as environmental hazards.

##### 3.1.2.2 Effect of dietary selenium on the egg selenium content

Se has also been used in animal feed to increase the production of functional (enriched) eggs, with enriched products delivering approximately 30–35 µg of Se, which could theoretically contribute approximately 50% of the recommended daily allowance (RDA) for humans (Fisinin et al., 2009), though rigorous human intervention trials confirming health outcomes remain limited. These Se-enriched eggs are now widely marketed in various countries globally, including the UK, Ireland, Mexico, and Australia, and are often priced similarly to free-range eggs.

Based on the literature, comparative studies of Scheideler et al. (2010), Li et al. (2024) and Han et al. (2017) showed that organic Se sources (e.g., Se-yeast and SeMet) generally deposited more Se into the whole egg, yolk (can reach up to 40% more), and albumen than did inorganic NaSeIII (P ≤ 0.001 and P ≤ 0.05, respectively) across the treatments examined, reflecting a similar pattern to that of meat (Section 3.1.2.1). Further characterization of these enrichment dynamics has been demonstrated by Lu et al. (2018), who observed a substantial elevation in egg Se concentration from day one of supplementation with 0.3 mg Se-yeast more than with NaSeIII, underscoring the rapid initial transfer of organic Se. Notably, this study demonstrated the positive linear and quadratic increment in egg Se over time for both organic forms. Building on this, a comprehensive dose-response comparison was presented by Wang et al. (2025), Lu et al. (2020), Lv et al. (2019), and Delezie et al. (2014). According to the majority of these studies, SeMet feed was as effective as Se-yeast at equivalent levels and much more efficient than NaSeIII for egg Se enrichment, with both organic sources producing a linear dose-dependent rise in egg Se content. However, Delezie et al. (2014) found that dietary SeMet exhibited the highest Se transfer to eggs, which was further supported by the findings of Asadi et al. (2017). Zhang et al. (2025b), Lu et al., (2019) and Bennett and Cheng (2010) have also demonstrated these dose-dependencies, though these studies raise questions about the physiological threshold for meaningful consumer benefit versus diminishing returns at supra-nutritional doses. In this context, although Zhang et al. (2025b) and Bennett and Cheng (2010) identified 1.5 and 5.1 mg/kg diet as the optimal inclusion level (no marginal Se enrichment attained with higher inclusion levels), tissue Se deposition associated with organic sources in spent hens has been observed at low doses (< 0.3 mg/kg DM diet), below the toxicity threshold. Interestingly, the distribution of Se within the egg is determined by Se form, in which SeMet preferentially enriches the yolk, whereas Se-yeast results in higher concentrations in the albumen (Li et al., 2024), a distinction crucial for targeted product labelling. These studies did not only report enrichment of eggs but also Se deposition in layer tissues and meat, increasing the market value of spent hens after their egg production cycle. This has also been demonstrated with Se-yeast at 0.3 mg Se-yeast/kg feed, in which Se concentrations in hen breast and egg were 1036.73% and 2127.93% higher than those of the control (Lu et al., 2019). Moreover, Yuan et al. (2011) showed that the retention of Se in broiler breeder eggs, developing embryos, and 1-day old chicks was progressively greater with organic Se-yeast and SeMet than in NaSeIII, underlining the crucial role of organic Se for vertical transfer to progeny.

The protective role of organic Se during PUFA-enriched egg storage represents an important and emerging area of research; as an example, Se-yeast demonstrated higher efficacy (39.6% and 2-fold elevation in whole egg and albumin, respectively) than NaSeIII, as well as preserved Haugh unit quality and reduced yolk TBARS during 30-day storage at 22°C (P < 0.05) (Zou et al., 2025). Further, Zhang et al. (2025b) characterized molecular modifications within the enriched oxidative stability, reporting upregulation for hepatic Nrf2, HO-1, CAT, and NQO1 antioxidant-related genes with the downregulation for Keap1 (P ≤ 0.05); however, under extreme high dose (6 mg/kg), elevations in relevant liver function enzyme (alanine aminotransferase, alkaline phosphatase and aspartate aminotransferase) and hepatocyte degeneration were observed, reinforcing the Se-yeast safety threshold in layer diets. However, it is important to note that the strategic combination of organic Se with micronutrients can further boost the functional value of eggs. According to Bahrami et al. (2020), the combination of organic Se, Vit E, Se, and marine oil not only enhanced yolk oxidative stability but also improved n-3 FAs while decreasing cholesterol and triglycerides.

The superior efficacy of other novel organic Se forms was also demonstrated. Mohammadsadeghi et al. (2024) demonstrated that selenized glucose (Se-Glu) can act as a highly alternative organic source, as a 0.6 mg/kg inclusion level elevated yolk and albumin Se contents, with decreased malondialdehyde levels in egg yolk (P ≤ 0.05), thereby enhancing oxidative stability of enriched eggs. Zhou et al. (2025) recently reported that Se-enriched lactobacilli represented a promising alternative to Se-yeast, in which both forms at 0.3 mg/kg feed enriched egg Se content and antioxidant networks of laying hens, although the lactobacilli form further increased the illeal lactobacilli abundance, pointing to a potential dual probiotic-Se functional benefit. In another study, Qiu et al. (2021) demonstrated the positive effect of Se conjugated to insect protein (SeCIP) on the production of Se-enriched eggs (P ≤ 0.05). Within this study, a linear dose-response (at 1-10 mg SeCIP/kg feed) has been observed. However, it is important to understand that the study of SeCIP did not rely on the exact Se supplementation level, which is likely a limitation since the exact Se inclusion level was unknown. Across identified records, few studies investigated the efficacy of dietary Nano-Se in enriching egg Se content, with these studies indicating lower effectiveness compared to organic sources. For example, dietary Nano-Se (0.5–1.1 mg/kg diet) had an insignificant effect on egg Se content compared to dietary Se-yeast, which significantly increased Se in yolk, albumen, and whole egg (Qin et al., 2019). Furthermore, at high inclusion rates of 2 mg/kg feed, SeMet showed greater efficacy (P ≤ 0.01) than Nano-Se and Se-yeast in Lohmann pink-shell eggs (Chen et al., 2024a).

Dietary Se yeast efficacy regarding eggs is not limited to layer hens; rather, it appears invasive across different poultry species. For instance, Se-yeast at 0.05-0.25 mg/kg produced a linear increase in Se in the yolk, albumen, and whole egg (P ≤ 0.05), with 0.25 mg/kg identified as the optimal inclusion level for Se-enriched duck eggs (Zhang et al., 2020a). In addition, according to Islam et al. (2024), Se-yeast at 1.5, 2.5, and 3.5 mg/kg feed resulted in higher Se levels in both yolk and albumen of quail eggs (P ≤ 0.05). Despite these outcomes indicating birds can benefit from organic Se forms, it is necessary to understand that efficacy may vary across species, breeds, genders, and ages.

***The above-reviewed studies*** involved Se supplementation in diets to improve the Se content in eggs, with varying efficiencies depending on the source. Consistent with findings in meat, organic sources generally provided a higher enrichment level than did inorganic NaSeIII, typically emerging in a dose dependent manner. Unlike the pattern observed in meat, Nano-Se depicted low efficacy. A few recent studies (e.g., Zhang et al. (2025b), Chen et al. (2024a), Qiu et al. (2021) and Bennett and Cheng (2010) employed inclusion levels (2-6 mg/kg complete feed) substantially exceeding the applicable regulatory maxima (0.2 and 0.3 mg/kg complete feed in the European Union and the United States, respectively). Although these studies reported enhanced Se deposition, the lack of comprehensive toxicological and environmental assessments at such supra-regulatory doses represents a challenge for regulatory approval and practical implementation, especially at the regional regulation level. Addressing this gap in part, though 1.5 mg/kg feed did not compromise the performance, egg quality, or health attributes over 12 weeks of feeding (Lu et al., 2019), it is yet unknown if such supra-regulatory doses are safe over the long term and across generations. Thus, until sufficient data support the safety of these doses, practical approaches must be in line with the established limits, which have been consistently proved effective for commercial Se-enriched egg production.

##### 3.1.2.3 Effect of dietary selenium on the milk selenium concentration

Most dairy farms have focused on producing fresh bovine milk with functional or health benefits (Han et al., 2021). Numerous studies in this area have determined Se in blood, which may indirectly reflect potential elevation in milk. However, in this section, we focused only on studies that directly determined Se concentration in milk.

Organic forms generally demonstrated superior efficacy concerning milk Se enrichment in the reviewed studies, although ruminant physiology represents unique challenges compared to monogastric animals, and the number of direct comparative studies in dairy species remains limited. In dairy cows, 0.2 and 0.3 mg/kg of Se-yeast supplementation has been observed to significantly increase (P ≤ 0.05) milk Se content (Kuusela et al., 2023; Ling et al., 2017; Gong et al., 2014; Zhang et al., 2018a), whereas 0.3 mg/kg of Nano-Se for 60 days boosted (P ≤ 0.05) both Holstein cow milk Se content and glutathione peroxidase level more than NaSeIII at equivalent doses (Han et al., 2021). The Se transfer rate into milk from dietary Se-yeast was previously characterized by Stockdale et al. (2011), who reported that for each 1 mg of dietary Se, 5.0 μg/kg milk Se was produced (R² = 0.97). Findings of these studies, specifically the distinct advantage of organic Se transfer into the milk, corroborate those of Stockdale and Gill (2011) in bovine milk (whose study included 5 dairy farms with different herd sizes and feeding systems) and confirm that the non-specific incorporation mechanism effectively bypasses the strict homeostatic regulation that limits inorganic Se secretion. Doyle et al. (2011) corroborated these findings by efficiently producing milk with uniformly high Se concentrations on six commercial dairy herds, demonstrating that the Se intake-milk relationship was predictable and attainable. Séboussi et al. (2016) explored an alternative approach relying on using Se-enriched forages, showing a relatively similar enrichment for milk Se level to that from dietary Se-yeast. This finding suggests that agricultural biofortification of fodder may serve as a supplementary technique for milk Se enrichment. In small ruminants, similar outcomes have been reported concerning dietary Nano-Se. In lactating Barki ewes, when bio-Nano-Se at 1.2 mg/kg feed was compared to NaSeIII, the novel form demonstrated better digestibility, milk yield and antioxidant status, alongside improvements in lamb growth performance (Khalil et al., 2023), reinforcing the strong efficacy concept of Nano-Se over inorganic Se in lactating ewes.

The efficacy of Se in ruminants is modulated by ruminal microbiome activity, which typically reduces unprotected organic Se forms into insoluble inorganic selenides (Hendawy et al., 2021), representing a limitation to their effectiveness. To counter this, OH-SeMet is regarded as an optimal source due to its resistance to rumen degradation and thermal stability during feed processing, notably pelleting/extrusion (Alkan & Murz, 2025). This form of Se appears to be more bioavailable than Se-yeast, wherein 205% and 171% increments were recorded in the milk of dairy cows supplemented with 0.3 mg OH-SeMet/kg DM and 0.3 mg Se-yeast/kg DM, respectively (Hachemi et al., 2023). Complementing these findings, Azorín et al. (2020) had earlier shown that the level of Se in milk and related dairy products (cheese and yogurt) elevated markedly (by 29.7%) when NaSeIII was partially replaced with Se-yeast (60:40 ratio of inorganic/organic at 0.240 mg Se/kg DM) in dairy cow diets, without any compromise in production. Hence, even moderate inclusion levels of organic Se are most likely capable of greatly enhancing the dairy product chain. Notably, the enrichment of milk and cheese had been reported earlier by Ling et al. (2017), demonstrating that dietary NaSeIII+ Se-yeast increased Se in milk (from 17.1 to 51.8 μg/kg) and in Edam-type cheese made therefrom (from 146 to 361 μg/kg).

Moreover, Se interacts synergistically with iodine (I), as demonstrated by Azorín et al. (2024) in bovine milk (P ≤ 0.05), upon both dietary Se-yeast (from *Saccharomyces cerevisiae)* and I supplementation. This study reported 3.29 µg Se/100 g of milk without altering overall milk quality, highlighting the feasibility of multi-nutrient fortification strategies. This multi-nutrient strategy was broadened to dairy goats by Azorín et al. (2025), who reported that dietary supplementation with both organic Se and iodine enriched their levels in milk (1.73-fold more), cheese (2-fold more), and yogurt (4-fold more), as well as maintained animal health and productivity. This strategy concept has been earlier supported by Zanetti et al. (2022), who reported organic Se, Vit E and sunflower oil enhanced Se and tocopherol concentrations, thus improving the nutritional profile without compromising the animal productivity.

In ***summary***, bypassing ruminal degradation is the determinant factor for successful enrichment, in which OH-SeMet and Nano-Se have shown promising outcomes. In addition, the Se+ I synergy emphasizes the necessity of considering the broader mineral matrix. However, as observed in meat and egg studies, high-dose strategies (e.g., Azorín et al. (2024); Zhang et al. (2018a)) lack comprehensive safety assessments. Future research must also investigate whether milk Se enrichment translates proportionally to Se-fortified dairy derivatives (e.g., cheese and yoghurt), thereby broadening the functional food market. In this context, the preliminary findings regarding Se retention in cheese and yogurt from Ling et al. (2017) and Azorín et al. (2025, 2020) are promising. A summary of the approaches used to improve Se content and their outcomes is presented in Table 1, which offers valuable insights into dairy farming practices.

**Table 1:** Summary of selenium sources, inclusion rates and levels, specific effects, and targeted animal products.

**Source**	**Levels of application and inclusion rates**	**Targeted animal product**	**Functional value effect on animal product**	**Authors**
NaSeIII Se-yeast	1 level: 0.3 mg/kg DM 1 level: 0.3 mg/kg DM	Beef/veal	Se-yeast increases Se content in the *Longissimus thorasis* muscle (P ≤ 0.01)	(Cozzi et al., 2011)
NaSeIII Se-Met	1 level: 0.3 mg/kg DM 1 level: 0.3 mg/kg DM	Broiler chicken breast and liver	SeMet supplementation markedly increased Se deposition (P ≤ 0.01).	(Wang et al., 2011)
NaSeIII Se-yeast SeMet	2 level: 0.15 and 0.3 mg/kg feed 2 level: 0.15 and 0.3 mg/kg feed 2 level: 0.15 and 0.3 mg/kg feed	Broiler chicken breast and liver	organic Se markedly increased Se deposition (P ≤ 0.01).	(Yuan et al., 2011)
NaSeIII Se-yeast	1 level: 0.3 mg/kg feed I level: 0.2 mg/kg feed	Pork	Both Se forms, especially Se-yeast, increased hepatic and muscle Se concentration (P ≤ 0.05), with no effect on meat quality	(Kawecka et al., 2013)
Se-yeast	2 levels: 0.1 and 1.0 mg/kg feed	Broiler chicken breast	High Se dose increased muscle Se levels (P ≤ 0.0001). also, it marginally increased n-3 EPA, and meat fat %	(Nyquist et al., 2013)
NaSeIII Se-yeast Nano-Se	1 level: 0.3 mg/kg feed 1 level: 0.3 mg/kg feed 1 level: 0.3 mg/kg feed	Broiler Chicken breast	Se-yeast induced higher Se deposition than NaSeIII (P ≤ 0.05); however, Nano-Se depicted comparable Se deposition to Se-yeast	(Liu et al., 2015a)
NaSeIII	1 level: 0.6 mg/kg DM	Goat meat/chevon	NaSeIII 0.6 mg/kg caused an increase Se in the *Longissimus lumborum* muscle (P ≤ 0.05)	(Aghwan et al., 2016)
Se-yeast	1 level: 0.3 mg/kg feed	Goose meat and liver	0.3 Se+ 100 mg Vit. E diet markedly increased Se content, 1.88-2.25-fold higher in meat and 1.68-fold greater in the liver	(Łukaszewicz et al., 2016)
Se-yeast+ Vit E	1 level: 0.3 mg Se-yeast+ 100 mg Vit E/kg feed			
NaSeIII Se-yeast	1 level: 0.3 mg NaSeIII+ 1.5% SO/kg feed 2 levels: 0.3 or 0.5 mg Se-yeast+ 1.5% of SO or LO/kg feed	Pork meat, liver, kidney and loin	Se-Yeast with LO increased Se deposition and n-3 FAs	(Jiang et al., 2017)
Se-yeast	1 level: 0.46 mg/kg feed	Rabbit muscles	2-fold increase in Se in hind leg and *Longissimus* meat	(Matics et al., 2017)
Se-yeast	3 levels: 0.1, 0.5, and 2.5 mg/kg feed	Rabbit muscles	dietary supplementation increased tissue Se content dose-dependently (P ≤ 0 .001). Also, it improved PUFA:SFA ratio. 0.5 mg/kg optimal for oxidative stability	(Papadomichelakis et al., 2017)
NaSeIII+ I+ probiotics	1 level: 0.35 mg NaSeIII+ 0.2 mg I/kg feed	Pork	more efficient Se/I accumulation in liver, heart, and meat vs. minerals without probiotic	(Zabashta et al., 2018)
NaSeIII SeMet Se-yeast Nano-Se	1 level: 0.1 and 0.3 mg/kg feed for each Se form	Broiler chicken breast, tight, liver and kidney	Nano-Se showed equal retention with the organic sources; however, both organic and nano forms exhibited greater retention compared to the inorganic NaSeIII.	(Bakhshalinejad et al., 2019)
SeMet NaSeIII SeMet+ NaSeIII	4 levels: 0.3, 0.4, 0.5 and 0.6 mg/kg feed 2 levels: 0.3 and 0.6 mg/kg feed 2 levels: SeMet 0.15 mg/kg+ NaSeIII 0.15 mg/kg, and NaSeIII 0.3 mg/kg+ SeMet 0.3 mg/kg feed	pork	SeMet at 0.4 ppm caused Se deposition in muscle (P ≤ 0 .05) NaSeIII 0.3 and 0.6 ppm caused Se deposition in the liver (P ≤ 0 .05)	(Silva et al., 2019)
NaSeIII Se-yeast	2 levels: 0.2 and 0.4 mg/kg feed 2 levels: 0.2 and 0.4 mg/kg feed	Broiler chicken meat, liver and heart	dietary Se-yeast resulted in greater Se accumulation (P ≤ 0.05). Se bio-availabilities of Se-yeast relative to NaSeIII (100%) were 111%–394% (p ≤ .05)	(Liu et al., 2020)
OH-SeMet+ Vit E	1 level: 0.5 mg+ 100 IU/kg feed	Mutton	increased muscle Se (P ≤ 0.0001). Se supplement lowered lipid peroxidation and improved microbial quality	(Bezerra et al., 2020)
NaSeIII Se-yeast	3 levels: 0.3, 0.9 and 2.7 mg/kg DM 3 levels: 0.3, 0.9 and 2.7 mg/kg DM	Beef	Se-yeast cause an increase in Se the muscles of cattle (P ≤ 0.001). 2.7 mg/kg produced meat with 372.7 μg Se/kg	(Silva et al., 2020)
NaSeIII Se-unicellular microorganism	1 level: 0.3 mg/kg feed 3 diets: 0.3 mg *Enterobacter cloacae Selenium*/kg feed, 0.3 mg *Klebsiella pneumoniae-Selenium*/kg feed, 0.3 mg *Stenotrophomonas maltophilia-Selenium*/kg feed	Broiler chicken breast	bacterial selenoprotein sources showed high Se deposition in the breast meat compared to NaSeIII (P ≤ 0.05). *Enterobacter cloacae* Selenium was associated with the highest breast meat Se content (P ≤ 0.05) *Klebsiella pneumoniae* and *Stenotrophomonas maltophilia* were not significantly different (P ≤ 0.05)	(Mohamed et al., 2020)
Se (not specified)+ Vit E	1 level: 2.5 mg/kg DM+ 500 IU vit E/kg DM+ 3% canola oil at feed	Beef	increased Se in meat, improved carcass fat deposition and tenderness	(Corrêa et al., 2021)
NaSeIII Se-yeast	1 level: 0.3 mg/kg feed 1 level: 0.3 mg/kg feed	Broiler chicken	Se-yeast improved oxidative stability and Se absorption vs. inorganic (P ≤ 0.01). this effect was enhanced with Phyto-biotic addition	(Konkol et al., 2021)
NaSeIII Se-yeast	1 level: 0.3 mg/kg feed 1 level: 0.3 mg/kg feed	Goose meat and liver	higher Se content in meat/liver with organic Se. No effect on chemical traits or fatty acid profile	(Nemati et al., 2021)
OH-SeMet	4 levels: 0.2, 0.4, 0.6, and 0.8 mg/kg feed	Breast of yellow feather broilers	OH-SeMet in diet linearly increased Se contents in the breast muscles (P ≤ 0.001)	(Tang et al., 2021)
NaSeIII Nano-Se	1 level: 0.15 mg/kg 3 level: 0.075, 0.15, and 0.3 mg/kg feed	Broiler chicken breast and thigh	Se increased significantly with dietary 0.3 mg Nano-Se/kg feed supplementation (P ≤ 0.05).	(Khajeh Bami et al., 2022)
NaSeIII Se-yeast Nano-Se	2 levels: 0.3 and 0.5 mg/kg feed 1 level: 0.5 mg/kg feed 1 level: 0.5 mg/kg feed	Broiler chicken breast and liver	Both Se-yeast and Nano-Se showed high bioavailability, and Nano-Se exhibited lower toxicity risk compared to other forms	(Bień et al., 2022)
NaSeIII Se-enriched plant	1 level: 0.5 mg/kg feed 2 level: 0.2 and 0.5 mg Se-enriched *Cardamine violifolia*/kg feed	Broiler chicken breast	Breast Se content significantly (P ≤ 0.05 increased by 14.4-127% in chicken fed Se-yeast	(Zhao et al., 2022)
NaSeIII SeMet	1 level: 0.2 mg/kg feed 1 level: 0.2 mg/kg feed	Quail meat	SeMet most effective for tissue Se. Feather Se correlated with muscle Se (R² = 0.714–0.756)	(Chantiratikul et al., 2021)
NaSeIII+ Se-yeast NaSeIII+ Nano-Se	1 level: 0.2 mg NaSeIII+ 0.2 mg Se-yeast/kg feed 1 level: 0.2 mg NaSeIII+ 0.2 mg Se-yeast/kg feed	Broiler chicken breast	both organic and nano forms increased breast Se content (P ≤ 0.05). Moreover, Nano-Se supplementation decreased MDA while improved n-3 FA profile	(Giamouri et al., 2023)
Se-yeast	3 levels: 0.1, 0.2, 0.4 mg/kg feed	Broiler chicken meat	quadratic Se deposition in tissues was observed	(Liu et al., 2023a)
NaSeIII SeMet Se-yeast Nano-Se	1 level: 0.5 mg/kg feed 1 level: 0.5 mg/kg feed 1 level: 0.5 mg/kg feed 1 level: 0.5 mg/kg feed	Broiler chicken meat	S-yeast best for Se deposition in liver and pectoral muscle; SM affected meat colour; all sources increased antioxidant capacity	(Chen et al., 2024a)
Se-yeast	3 levels: 1.5, 2.5, and 3.5 mg/kg feed	Japanese quail muscles, liver, kidney and heart	Higher Se in breast muscle, liver, kidney, heart (P ≤ 0.05).	(Islam et al., 2024)
NaSeIII Se-yeast	1 level: 0.3 mg/kg 3 levels: 1.0, 2.4, and 5.1 mg/kg diet	Egg	linear increase in egg Se content with dietary Se-yeast levels. No effect on production up to 5.1 μg/g	(Bennett & Cheng, 2010)
NaSeIII Se-yeast	2 levels: 0.55 and 0.75/kg feed 2 levels: 0.55 and 0.75/kg feed	Egg	organic Se more efficient for yolk Se deposition	(Scheideler et al., 2010)
NaSeIII SeMet Se-yeast	3 levels: 0.1, 0.3, and 0.5 mg/kg feed 3 levels: 0.1, 0.3, and 0.5 mg/kg feed 3 levels: 0.1, 0.3, and 0.5 mg/kg feed	Egg	organic Se remarkably increased Se deposition in egg compared to inorganic Se. SeMet showed the highest Se transfer to eggs, and organic sources more bioavailable	(Delezie et al., 2014)
NsSeIII Se-yeast SeMet	2 levels: 0.2 and 0.6 mg/kg feed 2 levels: 0.2 and 0.6 mg/kg feed 2 levels: 0.2 and 0.6 mg/kg feed	Egg	SeMet was most effective for egg Se deposition (P ≤ 0.01)	(Asadi et al., 2017)
Se-yeast NaSeIII+ Se-yeast	1 level: 0.3 mg/kg feed 1 level: 0.15 mg NaSeIII+ 0.15 mg Se-yeast/kg feed	Egg	Se concentration in egg (white albumen+ yolk) was significantly increased (P ≤ 0.05)	(Han et al., 2017)
NaSeIII Se-yeast	1 level: 0.3 mg/kg feed 1 level: 0.3 mg/kg feed	Egg	Se-yeast resulted in faster egg Se deposition vs. NaSe. Higher bioavailability recorded for organic Se.	(Lu et al., 2018)
Se-yeast	3 levels: 0.3, 0.6, and 1.2 mg Se/kg feed	Egg	increased egg white, yolk, and whole egg Se. The Se increase was dose dependent	(Lv et al., 2019)
Se-yeast	3 levels: 0.3, 1.5, or 3.0 mg/kg feed	Egg	Highest inclusion rate had 1036.73% egg Se content compared to control.	(Lu et al., 2019)
Se-yeast Nano-Se	3 levels: 0.5, 0.8, or 1.1 mg/kg 3 levels: 0.5, 0.8, or 1.1 mg/kg	Egg	Se yeast increased Se content in egg yolk, albumin and whole egg compared to Nano-Se and control groups. Nano-se effect was insignificant	(Qin et al., 2019)
NaSeIII Se-yeast	1 level: 0.3 mg/kg feed 4 levels: 0.1, 0.2, 0.3, and 0.4 mg/kg feed	Egg	Se-yeast markedly increased Se deposition efficiency (P ≤ 0.05)	(Lu et al., 2020)
Se yeast	3 levels: 0.05, 0.15, 0.25 mg/kg	Duck egg	Linear increase in Se in yolk, albumen, and whole egg (P ≤ 0.05). 0.25 mg/kg optimal for Se-enriched eggs	Zhang et al. (2020a)
SeCIP	4 levels: 1, 2, 5, or 10 mg SeCIP/kg feed	Egg	Se concentration in the egg was linear with the increase of dietary inclusion of SeCIP (P ≤ 0.05)	(Qiu et al., 2021)
Se-yeast SeMet Nano-Se	1 level: 2 mg/kg feed 1 level: 2 mg/kg feed 1 level: 2 mg/kg feed	Egg	egg Se content was highest in SeMet supplemented diet (P ≤ 0.01)	(Chen et al., 2024b)
NaSeIII Se-yeast Se-Met	2 levels: 0.15 and 0.30 mg/kg feed 2 levels: 0.15 and 0.30 mg/kg feed 2 levels: 0.15 and 0.30 mg/kg feed	Egg	overall, 0.30 mg Se/kg increase Se content compared to the 0.15 mg Se/kg in eggs (P ≤ 0.001) Se-Met supplemented diet had higher Se concentration in the yolk (P ≤ 0.05	(Li et al., 2024)
NaSeIII Se-Glu	2 levels: 0.3 and 0.6 mg/kg feed 2 levels: 0.3 and 0.6 mg/kg feed	Egg	0.6 mg Se-Glu/kg significantly enhanced the concentration of Se in yolk and egg albumen (P ≤ 0.05)	(Mohammadsadeghi et al., 2024)
Se-yeast	3 levels: 1.5, 2.5, and 3.5 mg/kg feed	Quail egg	higher Se in yolk and albumen (P ≤ 0.05)	(Islam et al., 2024)
Se-yeast	3 levels: 0.3, 0.5, and 0.9 mg/kg feed	Egg	increased egg Se: 0.32-0.977 mg/kg (P ≤ 0.01). Enhanced antioxidant capacity	(Wang et al., 2025)
Se-yeast	3 levels: 0.3, 1.5, and 6.0 mg/kg feed	Egg	dose dependent egg Se increase. 1.5 mg/kg optimal	(Zhang et al., 2025b)
Se-enrich bacteria Se-yeast	3 levels: 1.5, 3.0, and 6.0 mg/kg feed 3 levels: 1.5, 3.0, and 6.0 mg/kg feed	Egg	Both Se forms improved egg Se content; however, Se-enriched bacteria provided further probiotic benefits	(Zhou et al., 2025)
NaSeIII Se-yeast	1 level: 0.25 mg/kg feed 1 level: 0.25 mg/kg feed	DHA-enriched eggs	Se-yeast was more effective for egg Se deposition, recording about 40% and 2.4-fold increment in the whole egg and albumin, respectively	(Zou et al., 2025)
Se-yeast	2 levels: 35 and 51 mg/cow/day	Bovine milk	produced milk protein concentrate with 5.4 mg Se/kg	(Doyle et al., 2011)
Se-yeast	3 levels: 30, 40, or 60 mg Se/animal/day for 2 weeks 4 levels: 20, 30, 40, 60 mg Se/animal/day for 6 weeks	Bovine milk	a significant relationship was found between milk Se content and Se intake, in which Se level increased by 4.5 μg of Se/kg of milk for each mg of Se intake per day	(Stockdale & Gill, 2011)
Se-yeast (in pellets)	2 levels: 16 and 32 mg Se/cow/day	Bovine milk	milk Se responded quickly, which was markedly increased (P ≤ 0.05). Linear relationship: for each 1 mg dietary Se → 5.0 μg/kg milk Se (R^2^ = 0.97)	(Stockdale et al., 2011)
NaSeIII Se-yeast	1 level: 0.3 mg/kg DM 1 level: 0.3 mg/kg DM	Bovine milk	Se in milk was higher with organic Se-yeast inclusion than NaSeIII (P ≤ 0.05)	(Gong et al., 2014)
Se-fertilized forages NaSeIII Se-yeast	1 level: 0.79 mg/kg feed 1 level: 0.8 mg/kg feed 1 level: 0.7 mg/kg feed	Bovine milk	Se-fertilized forage: 11% more milk Se vs. Se-yeast (P ≤ 0.001) NaSe increased milk Se but It was significantly lower compared to Se-yeast and enriched forages (P ≤ 0.001)	(Séboussi et al., 2016)
NaSeIII+ Se-yeast	1 level: 0.2 mg NaSeIII+ 0.2 mg Se-yeast/kg TMR	Bovine milk and cheese	organic Se increased Se content in milk (from 17.1 to 51.8 μg/kg) and Edam-type cheese made there from (from 146 to 361 μg/kg)	(Ling et al., 2017)
NaSeIII Se-yeast NaSeIII+ Se-yeast	2 levels: 0.2 mg/kg and 0.4 mg/kg feed 2 levels: 0.2 mg/kg and 0.4 mg/kg feed 1 level: 0.2 mg/kg+ 0.2 mg Se-yeast/kg feed	Guanzhong dairy goat milk	all inclusion rates linearly increased the Se content in the goat milk; however, Se-yeast at 0.4 mg/kg diet increased Se in goat milk significantly (P ≤ 0.05)	(Zhang et al., 2018a)
NaSeIII+ Se-yeast	0.144 mg NaSeIII+ 0.096 mg Se-yeast/kg DM	Bovine milk and cheese	milk Se increased 29.7%, whereas cheese Se increased 38.2%	(Azorín et al., 2020)
NaSeIII Nano-Se	1 level: 0.3 mg/kg DM 1 level: 0.3 mg/kg DM	Bovine milk	Se and glutathione peroxidase activities were higher in groups supplemented with Nano-Se (P ≤ 0.05)	(Han et al., 2021)
OH-SeMet Se-yeast	1 level: 0.3 mg/kg DM 1 level: 0.3 mg/kg DM	Bovine milk	OH-SeMet resulted in 205% increment in Se in the milk, while 171% increment of Se in the milk from Se-yeast	(Hachemi et al., 2023)
NaSeIII Nano-Se	1 level: 1.2 mg/kg DM 1 level: 1.2 mg/kg DM	Ewe milk	Both dietary Se sources significantly increased milk Se; however, the Nano-Se depicted the highest milk Se content (P ≤ 0.01)	(Khalil et al., 2023)
NaSeIII Se-yeast	1 level: 0.2 mg/kg DM 1 level: 0.2 mg/kg DM	Bovine milk	Se-yeast supplementation doubled milk Se vs. NaSeIII	(Kuusela et al., 2023)
NaSeIII+ I NaSeIII+ I+ Se-yeast	For both: 0.57 mg/kg DM 0.34 mg NaSeIII+ 5.68 mg I+ 0.23 mg Se-yeast/kg DM	Bovine milk, cheese and yoghurt	levels of Se and I in milk were affected by diet and source (p ≤ 0.01). Se-yeast resulted in double Se deposition in milk (1.95 → 3.29). Moreover, cheese and yoghurt Se increased significantly	(Azorín et al., 2024)
NaSeIII+ Se-yeast+ I	0.23 mg NaSeIII+ 0.23 mg Se-yeast+ 5.68 mg I/kg DM	Goat milk, cheese, yogurt	1.73-fold more Se in milk, 2-fold more Se in cheese, and > 4-fold more I	Azorín et al. (2025)

Abbreviations: DM, dry matter; FA, fatty acids; I, iodine; Nano-Se, selenium nanoparticles; NaSeIII, sodium selenite; OH-SeMet, hydroxy selenomethionine; SeCIP, selenium conjugated insect protein; Se-Glu, selenized glucose; SeMet, selenomethionine; SO, soybean oil; Se-yeast, selenium yeast (mostly from *Saccharomyces cerevisiae*); Vit E, vitamin E.

#### 3.1.3 Key considerations and limitations in selenium supplementation strategies

**Fig. 5:**
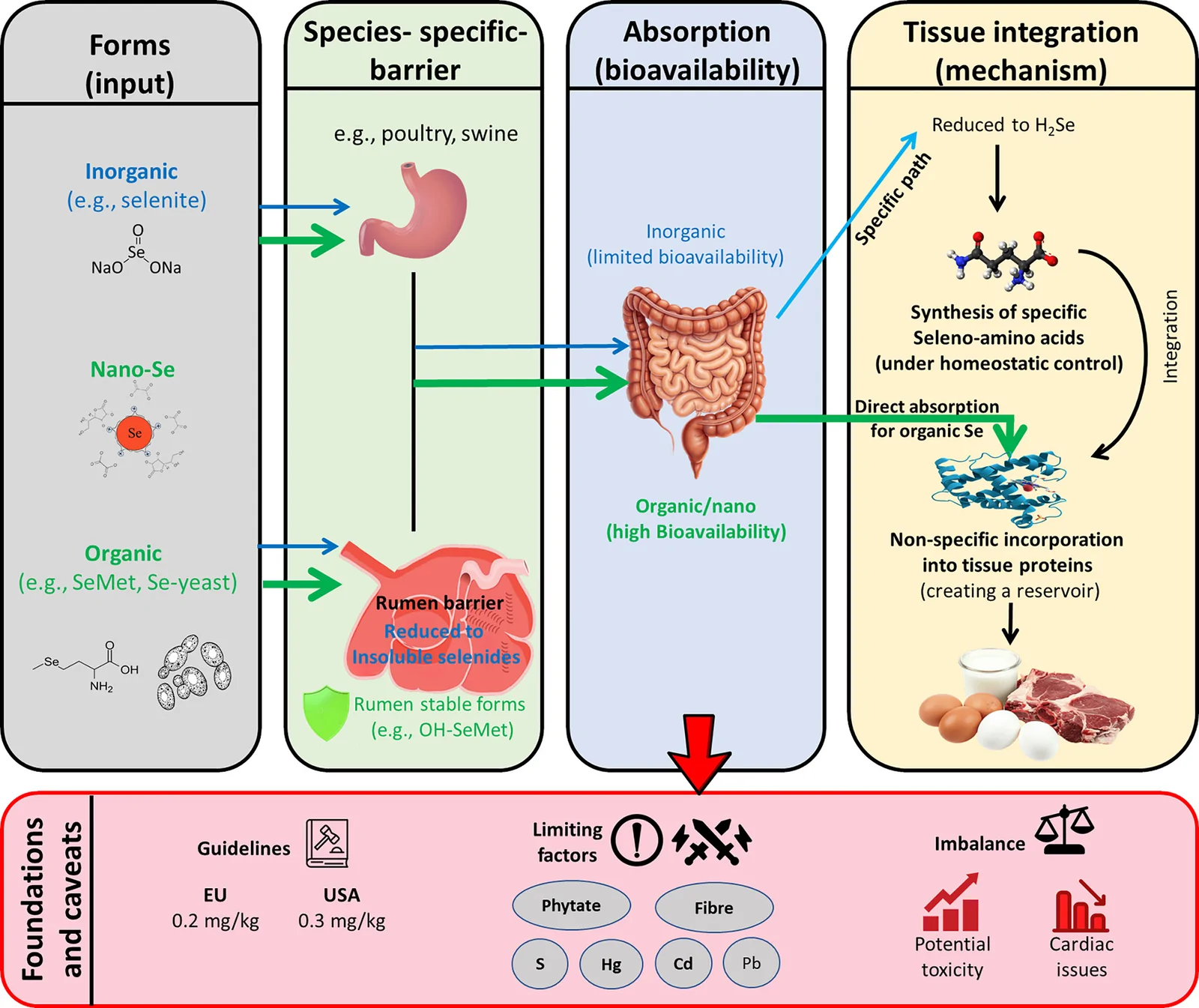
The selenium (Se) enrichment pathway and its strategic considerations.

The aforementioned recent literature corroborated earlier findings reported before 2010 (reviewed by Surai (2006)), manifesting that those organic sources (but more expensive sources) are more efficient at elevating Se levels in animal products than inorganic sources are. However, while the pattern of organic > inorganic is clear, several other ***factors*** are involved in the regulation of this pattern, including the inclusion level, supplementation period, animal species, target tissue and diet compositional matrix. These factors are likely critical for the success of developmental fortification strategies (see Fig. 5).

##### 3.1.3.1 Strategy efficacy and species-specific metabolism

Regarding approach efficacy, recent evidence has encouraged the use of various Se forms, in which mixed forms (organic+ inorganic) enhance antioxidant-related gene expression in reproductive tissues (Carr et al., 2022), pointing to the potential metabolic benefits of various Se supplements. Importantly, although Nano-Se (an encapsulated form) has shown more promising bio-accessibility than other inorganic Se forms do (as has been demonstrated in preliminary studies across animal species, as exemplified in poultry breast muscle (Łukaszewicz et al., 2016)), the limited number of studies, small sample sizes, and absence of long-term safety data warrant ***further investigation*** before practical applications, especially at high supplementation levels approaching or exceeding 0.5 mg/kg diet. For instance, the high surface area to mass ratio and unique quantum properties that amplified cellular uptake also pose theoretical disputes regarding uncontrolled intracellular reactivity and potential disruption of nontarget redox pathways. At the current time, long-term, multi-generational studies assessing potential nanotoxicity, tissue accumulation, and immunological trade-offs at pharmacological doses are severely lacking. Hence, future studies must focus on testing the Nano-Se’s efficacy, safety and environmental burden against organic sources like Se-yeast and OH-SeMet in large-scale and long-term trials. These studies/trials are of absolute importance for the regulatory limit revision. Accordingly, despite Nano-Se portraying a promising frontier, its commercial application development necessitates toxicological validation beyond standard Se safety thresholds to ensure regulatory conformity and consumer safety.

Apparently, ruminants pose a unique obstacle to nutritional strategies due to their ruminal microbial activity, which can degrade organic forms, although these sources are considerably more bioavailable than inorganic sources because of the high incorporation rate into proteins of ruminal microbes (about 4-fold more) and the biological value of these proteins (Hachemi & Sherlock, 2024; Spears, 2003; Surai et al., 2019). This attribute makes rumen-stable sources like OH-SeMet (Alkan & Murz, 2025; Hachemi et al., 2023) and Nano-Se (Hachemi et al., 2023) particularly advantageous for dairy and beef production. Furthermore, the ruminant metabolism of Se intersects uniquely with lipid metabolism, in which Czauderna et al. (2012) reported that inorganic NaSeVI can actively upregulate ∆9-desaturation pathways in lambs, increasing CLA deposition in adipose tissue when co-supplemented with linseed oil. Among OH-SeMet and nano forms, Nano-Se may further enhance ruminal function, as a 0.2 mg/kg inclusion level has been reported to augment feed degradation and fermentation, hence elevating the generation of volatile FAs (VFAs), including acetic, propionic, and butyric acids (Shakweer et al., 2023). These effects' underlying mechanisms most likely function through several simultaneous channels: (1) Se acts as a cofactor for selenoproteins within rumen microbes (notably thioredoxin reductase), supporting microbial antioxidant status and metabolic efficiency under the naturally reducing ruminal environment; (2) more effective microbial selenoprotein incorporation may be made possible by Nano-Se's large surface area and better solubility than ionic selenite, augmenting the metabolic capacity of cellulolytic bacteria; and (3) modulation of the intraruminal redox environment by nano-sized elemental Se particles may selectively favour fibrolytic bacterial populations (*Bacteroidetes* and *Ruminococcaceae*) and competitively suppress hydrogenotrophic methanogenic archaea by reducing available hydrogen substrate. All of these processes would account for the corresponding decline in methanogenic archaea and the apparent increases in Bacteroidetes and Firmicutes populations (Rabee et al., 2023), though direct experimental evidence for each pathway remains to be established and represents an important priority for future research.

The mechanism by which Nano-Se augments ruminal fermentation likely involves Se-dependent enhancement of microbial selenoprotein synthesis, optimization of the intraruminal redox environment favouring cellulolytic bacteria, and direct modulation of hydrogenotrophic methanogen activity, although the specific mechanisms have yet to be confirmed experimentally. Hence, there is a lack of knowledge in this area of Se effects on ruminal microbiota and fermentation, in which future studies need to uncover the Se effect and its mechanism of action on the ruminal microbiome to optimize Se delivery for ruminant-specific benefits.

***Species-specific metabolism*** appeared to affect Se efficacy and, consequently, product enrichment outcomes. Ruminants demonstrate lower net dietary Se bioavailability owing to microbial sequestration within their foregut systems, where sulfate-reducing bacteria partially convert inorganic Se to biologically unavailable insoluble selenide complexes (Se^2-^) and, accordingly, reduce the fraction available for intestinal absorption. Despite this ruminal transformation, the superiority of organic form is an absolute fact and shown across studies (e.g., Stockdale and Gill, 2011), as the non-specific incorporation pathway allows the organic form to bypass systemic homeostatic bottlenecks that limit inorganic Se secretion. Nevertheless, paradoxically, monogastric animals have higher recommended dietary Se levels (0.2–0.3 mg Se/kg diet for swine (FDA, 2003) and 0.15–0.5 mg Se/kg feed for poultry (Wang et al., 2022)) than those normally sufficient for ruminants under equivalent productivity conditions. These higher requirements for monogastric animals are mainly attributed to heightened selenoprotein synthesis and turnover (e.g., glutathione peroxidases GPx1/GPx4, thioredoxin reductase TrxR1, selenoprotein P), rapid metabolic and growth rates (Li et al., 2023a; Surai & Kochish, 2019), high feed throughput, and their reduced gastrointestinal retention times (especially in avian species). In essence, the higher supplementation needs of swine and poultry are driven by metabolic demand rather than poor intestinal absorption efficiency per se. The organic-Se and monogastric lipid metabolism interaction is of importance, as Jiang et al. (2017) showed that the combination of Se-yeast and linseed oil synergistically improved pork quality by drastically reducing drip loss (58–74%), an affect not seen with inorganic-Se or conventional vegetable oils, underscoring a species-specific metabolic synergy. Furthermore, Saleh et al. (2013) showed the combination of organic Se and linseed oil in a growing rabbit diet improved growth performance and glutathione activity, whereas abdominal fat and total cholesterol were decreased. However, to avoid potential health consequences like white muscle disease (in calves and lambs) and alkali disease (in multiple species), Se supplementation is advised not to exceed 0.3 (organic forms) or 0.5 (inorganic sources) mg/kg of diet under normal physiological health statuses, complying with legislations.

##### 3.1.3.2 Dietary matrix and antagonists

The bioavailability and function of dietary Se are affected by various dietary combinations, with Se acting as both a therapeutic agent and a nutrient prone to antagonistic elements. Synergistic ***interactions*** between antioxidant vitamins (C+ E) improve oxidative defence systems (Liu et al., 2008); however, it is worth noting that under certain conditions (like supraphysiological supplementation doses, in the presence of redox-active transition metals (e.g., Fe²⁺, Cu²⁺), or at very high oxidative loads where the tocopheroxyl radical cannot be efficiently regenerated), these vitamins may act as prooxidants rather than antioxidants (Abudu et al., 2004; Chen et al., 2009), which underscores the importance of tailoring supplemental strategies. Typically, the limits of Se’s protective role are exposed under high unsaturation levels, as Se-yeast alone at 0.25 mg/kg feed failed to counteract the pro-oxidant effects of a 4% fish oil diet in rabbits (Danuta et al., 2020), elevating malondialdehyde levels and rancidity rates, thereby underscoring the importance of antioxidant mixture supplementation (like Se+ vitamins) in high unsaturated matrices. Reciprocally, appropriate combinations of Se and vegetable oils (e.g., Se-yeast and linseed oil) result in synergistic benefits, as demonstrated by Jiang et al. (2017) regarding the pork water-holding capacity, and, as shown by Yin et al. (2025), the combined dietary SeMet+ fish oil supplementation markedly reduced lipid peroxidation. However, the specific interaction between Se and other antioxidants is highly form-dependent, as demonstrated in chicken muscles after the consumption of a diet enriched in Vit E and inorganic NaSeVI, wherein they acted as pro-oxidants compared to Se-yeast (Rozbicka-Wieczorek et al., 2014), highlighting that inorganic Se forms carry inherent oxidative risks that demand strict regulatory oversight. This points out that Se can antagonize the oxidative balance in a highly unsaturated diet, making the Se form selection a very critical step toward the maintenance of the antioxidant network. In addition, Se with other dietary macronutrients has been observed through studies to interact synergistically, as Mohammadsadeghi et al. (2024) showed that Se-Glu not only increased egg Se content but also significantly reduced yolk malondialdehydes, while the co-supplementation of antioxidant vitamins (e.g., Vit E) increased Se deposition in goose muscle (Łukaszewicz et al., 2016). In a similar manner, Se and I provided positive synergism in enriching both elements in bovine milk (Azorín et al., 2024). Moreover, Se can positively modulate the FA profile (Del Puerto et al., 2017), an effect beyond antioxidant protection, leading to healthier attributes in products.

On the other hand, sulfur and organic Se (especially Se amino acids like methionine and cysteine) interact competitively, in which both elements share similar transporters (e.g., the sodium-dependent neutral amino acid transporter (B⁰ system) (Wolffram et al., 1989)), whereas in ruminants, excessive sulfur is metabolized by ruminal sulfate-reducing bacteria to sulfide (S²⁻) (Coleman, 1960), which reacts with Se to form insoluble selenide complexes (e.g., SeS or Se-S minerals) that reduce Se bioavailability (Arshad et al., 2021; Ivancic & Weiss, 2001; Van Ryssen et al., 1998). These elements have similar metabolic pathways, allowing Se to substitute sulfur in amino acids, which likely alters the stability and functionality of selenoproteins (Schlegel et al., 2016). The antinutritional component phytate has the capacity to decrease the bioavailability of minerals like Se, Zn, iron and manganese (Schlegel et al., 2016). Diets high in phytates (e.g., based on rapeseed and soybean meals) have been shown to reduce Se deposition in animals by forming insoluble Se-phytate-ion complexes. This pattern is likely more restricted to inorganic sources and more frequently occurs in monogastric animals than in ruminants, as the ruminal microflora metabolizes phytate (Spears, 2003; Takahashi et al., 2017). Thus, phytase addition to monogastric diets is of great importance to enhance Se bioaccessibility. ***Cyanogenic glycosides*** in legumes further alter Se deposition and excretion (Gu & Gao, 2022). Although the underlying direct mechanisms remain unclear, several plausible pathways may be operative: cyanogenic glycosides are enzymatically hydrolyzed to hydrogen cyanide (HCN) and thiocyanate (SCN⁻) in the gastrointestinal tract; thiocyanate is a structural analogue of selenocyanate (SeCN⁻) and may competitively inhibit Se absorption at shared anion transport sites (notably the NaSi-1/SLC13A1 transporter for inorganic Se). Additionally, cyanide may react with selenide intermediates in the metabolic pathway of inorganic Se, potentially redirecting Se towards alternative excretory routes. Furthermore, thiocyanate-induced perturbation of thyroid function (via competitive inhibition of iodide transport) may indirectly alter selenoprotein expression profiles in thyroid tissue. Elucidating these mechanisms through controlled *in vivo* studies in relevant livestock species represents a worthwhile future research priority, particularly given the prevalence of legume-based feedstuffs in ruminant and monogastric diets. It is also necessary to mention that heavy metals (e.g., mercury (Hg²⁺), cadmium (Cd²⁺), and lead (Pb²⁺)) can also form insoluble complexes with Se, reducing Se absorption but concurrently lowering heavy metal bioavailability from the intestinal canal, highlighting Se’s potential detoxification role in contaminated environments/regions (Food and Drug Administration (FDA), 2025; Hejna et al., 2018). Therefore, these aforementioned interactions necessitate precision in diet formulation to balance Se’s dual role as a nutrient and mitigator of dietary antagonists.

##### 3.1.3.3 Potential selenium toxicity

Although Se is an essential element in human nutrition, daily Se intake levels (above 400 µg) are recommended to avoid ***potential toxicity***. In relation to animals, potential toxicity can be exhibited, resulting in the establishment of legislation. In this regard, several pieces of legislation are available, which vary considerably between jurisdictions, with further distinctions driven by the concept of regulatory maxima (restrictions that are enforceable by law) and nutritional recommendations (non-binding dietary recommendations for the best possible health and performance of animals). As an illustration, within the European Union, 0.2 mg of organic Se source (e.g., Se-yeast and SeMet)/kg and 0.5 mg of inorganic Se source (like NaSeIII and NaSeVI)/kg are the current maximum total Se levels in complete feed, on an as-fed basis, assuming 88% dry matter (DM) content (Bampidis et al., 2019, 2021, 2024). These established limits focus on the total sum of dietary Se in complete feed, which comprises both the naturally occurring Se in feed and the supplemented forms. Of note, the relatively low maxima of organic Se have been driven by their higher bioavailability, and, consequently, the greater toxicity potential of organic forms relative to inorganic sources. In the United States, a relatively higher maximum inclusion rate of 0.3 mg/kg of complete feed has been established for all livestock species (Food and Drug Administration (FDA), 2025); however, it is important to understand this limit applies only based to dietary supplements and does not account naturally occurring Se in feed, thereby the total dietary Se level may exceed 0.3 mg/kg. A further marked distinction from the European Union jurisdictions is that the established 0.3 mg/kg in the United States does not differentiate organic and inorganic forms. It is essential to distinguish these regulatory maxima from nutritional recommendations, such as those provided by the National Research Council (NRC) for various livestock species, which typically suggest lower dietary Se inclusion levels aimed at meeting the animal’s requirements for selenoprotein synthesis and optimal immune function. For example, NRC guidelines for swine and poultry generally recommend 0.15-0.3 mg Se/kg diet (NRC, 2026, 2012), depending on the species and production stage, which falls at or below the applicable regulatory maxima.

Within the literature investigated, several studies employed inclusion levels that substantially exceed the applicable regulatory maxima. For instance, Zhang et al. (2025b) clearly demonstrated the toxicity in layer hens fed up to 6 mg Se-yeast/kg feed, which is 30 times above the European established maxima. Bennett and Cheng (2010) also fed hens a diet with a relatively similar Se inclusion level: 5.1 mg/kg feed. Chen et al. (2024b) used SeMet at 2 mg/kg feed, representing a 10-fold increase over the European Union maximum for organic Se (0.2 mg/kg complete feed) and approximately a 6.7-fold increase over the FDA supplemental maximum (0.3 mg/kg complete feed). Silva et al. (2020) also used inclusion levels (2.7 mg of NaSeIII or Se-yeast/kg DM) exceeding the regulatory maxima by 5.4- and 13.5-fold for NaSeIII and Se-yeast, respectively. Nyquist et al. (2013) and Lv et al. (2019) employed 1 and 1.2 mg Se-yeast/kg diet, which is 5 and 6-fold the established European Union maxima for organic Se source, respectively. Qiu et al. (2021) employed SeCIP at up to 10 mg/kg feed, although the exact Se contribution from this source was not quantified. Even though these studies (except for Zhang et al., 2025b) reported enhanced Se deposition and, in some cases, additional beneficial modulations, neither a comprehensive toxicological assessment (e.g., tissue pathology, reproductive parameters, multi-generational effects) nor an environmental assessment (e.g., Se excretion in manure and soil accumulation) was conducted at these supra-regulatory doses. The Oregon Department of Agriculture mandates the labelling of Se-supplemented feedstuffs to prevent mishandling and potential toxicity (Filley et al., 2014). Until such data are available, practical approaches must remain within established regulatory limits, which have been consistently shown to be effective for commercial Se-enriched product production.

Concerning health aspects, future investigations are needed to ensure the Se additive regulatory limits, especially for novel forms that emerge with technological advancements, as, for example, inorganic forms of Se were approved for supplementation in livestock diets as early as 1974, whereas organic Se-yeast gained approval as a feed additive in 2003 (Filley et al., 2014). Among different Se forms, the emerging nano-form is of great concern. Despite its demonstrated superior deposition in muscle tissues (Łukaszewicz et al., 2016) as well as rumen fermentation benefits compared to inorganic forms (Hachemi et al., 2023), the regulatory approval and long-term safety profiling of nano-forms remain nascent. Further theoretical concerns, like uncontrolled intracellular reactivity and potential disruption of nontarget redox pathways, rise from the high surface area to mass ratio and the unique quantum properties for enhancing cellular uptake. There are currently relatively few long-term, multigenerational investigations evaluating possible nanotoxicity, tissue accumulation ceilings, and immunological trade-offs at pharmaceutical levels. To ensure regulatory compliance and customer safety, Nano-Se's commercial application demands detailed toxicological validation above traditional Se safety standards, even though it represents a promising frontier. Moreover, Se supplementation strategies must consider animal species, dietary interactions, and the form of Se to optimize health outcomes and ensure its efficient use.

##### 3.1.3.4 Selenium section brief summary

Based on the reviewed studies, organic and nano-forms are superior to inorganic forms regarding enrichment strategies, mainly achieved via non-specific incorporation. Rumen-protected forms (like OH-SeMet) are most likely more effective for ruminants. However, a critical gap remains in understanding the long-term toxicological and environmental implications of high dose and high efficacy sources like nano-forms, alongside the human critical trials validating the health benefits of consuming Se-enriched animal foods. In addition, as demonstrated, Se-yeast could not protect against induced oxidation by 4% fish oil and the pro-oxidant risk identified with inorganic Se forms; therefore, the precise scaling of Se alongside other antioxidants and dietary unsaturated oils is urgently needed to ensure product stability and long shelf life. Moreover, exploring the synergistic effects of organic forms and the lack of synergism in inorganic forms, along with the role of organic ligands, would greatly benefit the field of multi-nutrient functional foods.

### 3.2 Zinc (Zn)

Zinc (Zn) is a fundamental trace element for the growth, development, reproduction and general health of farm animals through impacting cellular and humoral immunity, cytokine release, nucleic acid metabolism, apoptosis and signal transduction. In addition, Zn is essential for metabolism and enzymatic functions, being a catalytic or structural component for over 300 confirmed metalloenzymes in mammals (contributing to hydrolases, transferases, oxidoreductases, lyases, ligases, and isomerases (Duan et al., 2023; Maret, 2013; Vallee & Falchuk, 1993)), while, beyond catalytic roles, structural zinc-binding motifs (e.g., zinc fingers) are estimated to exist in approximately 10% of the mammalian proteome (∼2,800 proteins), reflecting Zn's pervasive involvement in gene regulation, protein folding, and signal transduction (Andreini et al., 2006b, 2006a; Maret, 2013), as well as its strong contribution to the overall antioxidant capacity that stabilizes cell membranes (Chvapil, 1973). Despite Zn’s positive roles in animal health and performance, its use in enrichment strategies is likely more challenging than for Se due to the body’s strict homeostatic regulation over Zn status, mainly regulated at the physiological level through recyclability by the pancreas, endogenous secretion, intestinal absorption, renal excretion and health status (Bechoff & Dhuique-Mayer, 2017; Li et al., 2019a; Oberleas & Harland, 2008). This is the fundamental reason why high doses are often needed for enrichment and why source bioavailability is so critical. Zn at high levels is also commonly administered as an immune booster; thus, it has anti-diarrheal effects in the early life phases of animals (Rajaei-Sharifabadi et al., 2024), a strategy in which Zn-oxide (ZnO) is initially administered as an antidiarrheal agent, followed by Zn methionine (ZnMet) to promote growth. The benefits of Zn supplementation extend beyond animal health aspects to include the consumption of Zn-enriched products.

#### 3.2.1 Zinc sources and bioavailability: a key to enrichment

**Fig. 6:**
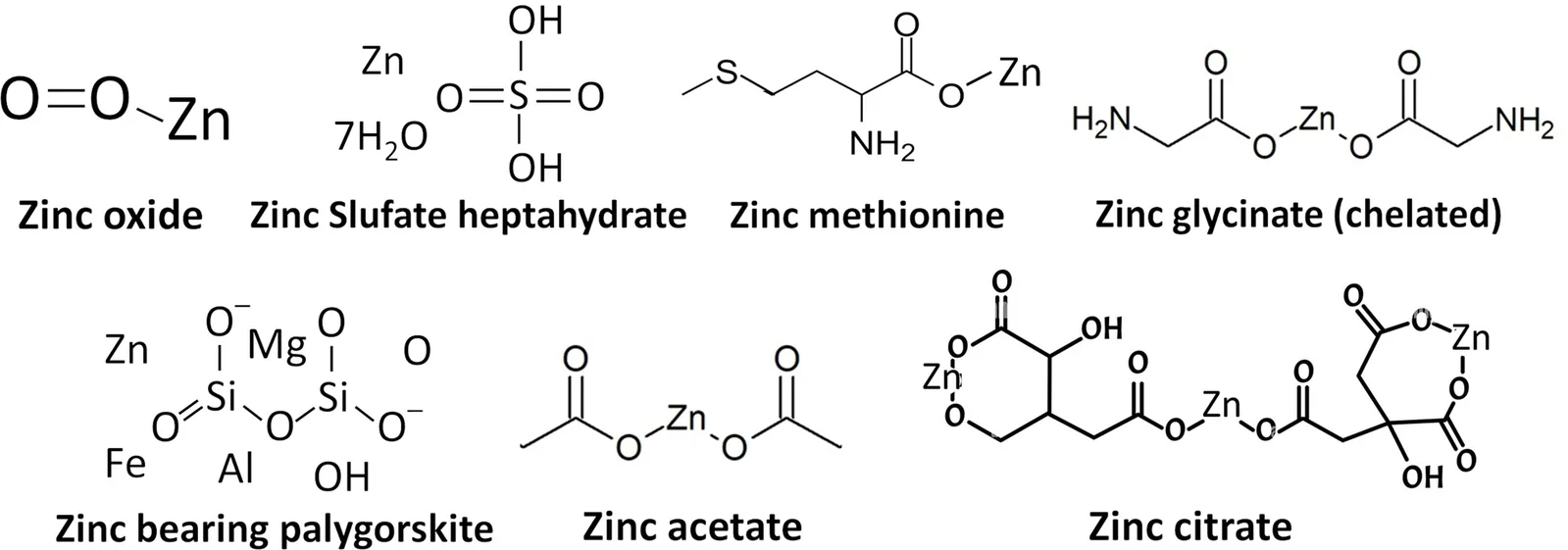
Structures of different dietary zinc sources mentioned in this review.

In nature, the Zn element can be found in different forms, most frequently as sphalerite (ZnS), as well as smithsonite (ZnCO_3_), zincite (ZnO) and hemimorphite (Zn_4_Si_2_O_7_(OH)_2_·H_2_O), which have also been identified in nature. From a nutritional relevance perspective, in practice, Zn is often administered as inorganic salts (e.g., ZnO, ZnSO_4_ and Zn-bearing palygorskite (ZnPal)) or organic sources (bound to amino acids, chelated compounds (e.g., glycinate and gluconate), acetate, or citric acid) (see Fig. 6), which has been the focus of the recent literature on animal nutrition, as illustrated in the following sections.

The critical factor for fortification is bioavailability, which is superior through organic sources, notably in monogastric animals (as discussed in 3.2.2, 3.2.3). This is because inorganic Zn²⁺ is highly susceptible to forming insoluble complexes with dietary phytates, drastically reducing its absorption (Grases et al., 2004; Philippi et al., 2023). In contrast, organic Zn sources are typically more stable (maintain solubility) in the intestinal lumen and invulnerable to phytate (demonstrating less reactivity with phytate), thus resisting precipitation as insoluble phytate complexes. Zn chelated to small peptides or amino acids may exploit peptide transporter 1 (PepT1/SLC15A1) and specific amino acid carriers for absorption, partially bypassing competition with other divalent metal ions at shared importers such as divalent metal transporter 1 (DMT1). ZIP family importers (ZIP1–4, ZIP8, ZIP14; encoded by SLC39A genes) mediate Zn²⁺ influx into enterocytes from the apical lumen and from endosomal compartments. Following cellular uptake, Zn is released from the organic ligand intracellularly and enters the common Zn pool, with basolateral export mediated by Zn transporter 1 (ZnT1: SLC30A1) into portal circulation (Cousins, 2010; Eide, 2006; Yuzbasiyan-Gurkan & Bartlett, 2006), referring, overall, to organic forms with high bioavailability, especially in high-phytate diets like corn-soybean meal. Herein, three mechanisms/events may explain this organic source superiority, such as (1) the superior resistance to luminal precipitation in the form of intractable phytate complexes; (2) partial absorption through PepT1, which bypasses DMT1 competition; and (3) intracellular dissociation from the organic ligand to join the common zinc pool after absorption (Cousins, 2010; Eide, 2006; Yuzbasiyan-Gurkan & Bartlett, 2006). Unlike Se, Zn can be directly absorbed and incorporated into animal tissues (Zn proteome, yet it is not part of the protein itself but rather attached to the side chain) as a free divalent ion, and it typically dissociates from amino acids upon uptake by the cell. While organic Zn sources typically have superior bioavailability compared with inorganic Zn sources (Devarshi et al., 2024), absorption rates (approx. 5–80% (Robles Jimenez et al., 2021)) depend on the animal body’s total Zn (Byrne & Murphy, 2022). This emphasizes the relationship between the importance of the efficacy of enrichment strategies and the Zn pool within the body, in which the digestive and renal systems play a crucial role in determining bioavailability, as discussed in section 3.2.3. However, excess Zn, to a certain limit, is typically stored in the liver and other tissues, mainly through binding to metallothionine, a protein rich in cysteine that acts as a buffer for the free Zn pool.

#### 3.2.2 Effects of dietary zinc on the meat, egg and milk zinc contents

Successful Zn enrichment requires dietary levels that surpass the animal's immediate metabolic requirements to overcome homeostatic barriers (Section 3.2.1), which is unlike the case of Se. In this regard, a Zn enrichment strategy often requires dietary levels that surpass immediate metabolic requirements to overcome these physiological barriers. Ruminants generally require ≤ 50 mg Zn/kg DM (except goats: 10–80 mg/kg DM), while swine and poultry require Zn levels > 50 mg/kg DM to support their rapid growth rate, early development, and egg production (EFSA, 2014).

In ***meat***, no study found regarding the enrichment of pork and beef, with only studies available on poultry. For example, dietary Zn from all three tested sources (ZnSO4·7H2O, Zn-amino acid chelate, and Zn proteinate) at 60, 120, and 180 mg/kg feed increased Zn content in broiler liver, breast, and thigh muscles (P ≤ 0.01), with no difference recorded among the different sources (Liu et al., 2015b). Furthermore, Zn deposition in the thigh and breast (*pectoralis major* muscle) has been shown to be dose-dependent. As an example, dietary Zn-Pal (also known as attapulgite) at 0, 20, 40, 60, and 80 mg/kg inclusion levels increased Zn deposition in broiler thigh and breast in a corresponding manner (Yang et al., 2016). In a form comparison, despite that Zn-Pal at just 20 mg/kg stimulated muscular accumulation, Zn sulfate heptahydrate (ZnSO_4_·7H_2_O) required substantially higher inclusion levels to maximize retention in breast and duck muscles. According to Zhang et al. (2018b), when ZnSO_4_·7H_2_O was offered to growing broilers at 0, 16, 32, 48, 64, 80, or 96 mg/kg feed, the highest Zn retention was observed at 80 mg Zn/kg feed (P ≤ 0.05). However, Wen et al. (2019) also studied the effects of ZnSO_4_·7H_2_O supplementation on Zn accumulation in Peking ducks, in which 0, 15, 30, 60, 120, and 240 mg Zn/kg were administered for 35 days. This study reported that Zn deposition in duck breast muscle and liver linearly increased with increasing inclusion of Zn (P ≤ 0.05), and on the basis of the linear regression model, 91.32 mg Zn/kg feed was suggested as the optimal dosage. This suggested optimal level falls within the recommended European Union regulatory limit. However, it is important to note that although these studies showed the efficacy of ZnSO_4_·7H_2_O (no adverse health outcomes reported), some inclusion levels (e.g., up to 240 mg Zn/kg in Peking duck complete feed) approach or exceed the current European Union regulatory maximum (120 mg/kg for turkeys, 100 mg/kg for other poultry (EFSA, 2014)), although no adverse health outcomes were reported in these short-term experiments. This substantial gap between experimentally effective doses and regulatory limits highlights a key challenge in Zn enrichment balance between overcoming homeostasis and maintaining compliance. In this regard, long-term studies at compliant doses with comprehensive safety assessment (entire animal tissues) are needed before these findings can be translated into practical recommendations.

With respect to Zn supplementation in laying hens to improve ***egg*** functional value, both inorganic and organic source revealed efficacy, with organic and nano-forms exhibit tangible advantages by overcoming homeostatic barriers. For example, when Bahakaim et al. (2014) examined the effects of different levels (up to 150 mg/kg feed) and sources of Zn on the eggs of Golden Montazah Egyptian laying hens, both ZnSO_4_.7H_2_O and organic ZnMet significantly (P ≤ 0.05) increased the egg Zn content without detrimental effects on egg production. The benefit of organic Zn for laying hens was validated by Yu et al. (2020), who found that dietary ZnMet or ZnO at 35 and 70 mg/kg feed remarkably increased egg Zn content (in a linear relationship for both organic and inorganic sources) and serum Cu–Zn SOD activity. Further comparison was conducted by Behjatian Esfahani et al. (2021), who assessed Zn-threonine (ZnThr, Zn-Met, and ZnO at 30, 60, and 90 mg/kg in laying hens. In comparison to ZnO, both organic chelates at relatively lower levels induced greater Zn deposition in eggs and decreased Zn excretion in faeces; moreover, Zn-Thr had the most effective relative bioavailability of the sources examined. Outcomes of this study clearly reflect the superiority of organic additives over inorganic ones. Complementing these outcomes, Kannan et al. (2022) reported that 80 mg Zn-propionate/kg feed enhanced egg quality, antioxidant indices and Zn content, while higher levels (120–160 mg/kg) were not further effective compared to Zn-propionate; accordingly, regardless of the source, Zn enrichment offers low returns when homeostatic needs are exceeded. Extending beyond chickens, duck eggs were as well responsive for dietary Zn. As an illustration, egg Zn level has been reported to increase linearly and quadratically with dietary ZnSO_4_ inclusion (0–160 mg/kg complete feed) (Zhang et al., 2020b), with an estimated 65–80 mg/kg feed (above the baseline of 27.7 mg/kg) being recommended as the optimal dietary concentration for laying performance, suggesting that duck species-specific Zn requirements for egg enrichment are broadly consistent with those of chickens, though not identical.

Novel Zn forms were investigated as well, depicting a promising enrichment tool. For example, a study by Abedini et al. (2018) revealed a dose-dependent increase trend related to dietary Zn oxide nanoparticles (ZnO-NPs) (from 50 to 150 mg/kg diet). However, Zn deposition in layer eggs was cost effective at 80 mg ZnO-NPs/kg, as no significant difference was found when compared with the maximum dietary dose, although the respective Zn contents were highest at 11.06 and 11.51 μg/g, respectively. Consequently, this study emerged with various benefits, majorly cost effective and within compliant thresholds. The role of multi-minerals inclusion was also assessed, in which Ullah et al. (2024) showed that supplementing laying hen diets with both iron and Zn may concurrently enrich egg yolks with both minerals; despite this, careful balancing of the optimal deposition levels was necessary to prevent competitive antagonism between these divalent cations. Of note, this study has employed various levels, among which the 300 mg/kg exceeds the current European Union-established limits, warranting further investigations. Moreover, balancing dietary iron and zinc levels is crucial to reducing their competitive interaction (see Section 3.2.3.1).

In dairy animals, the focus has been on organic sources; however, studies are generally limited and focused less on Zn-enriched milk, aiming for both ***milk*** enrichment and udder health. The limited literature on Zn-enriched milk likely reflects the tight homeostatic control of Zn secretion (unlike Se that undergoes non-selective incorporation into milk proteins via methionine metabolic pathways) by mammary gland-specific exporters ZnT2 (SLC30A2) and ZnT4 (SLC30A4), which regulate Zn partitioning from epithelial cytoplasm into Golgi vesicles and, ultimately, into milk, operating mostly independently of systemic Zn status. This homeostatic mechanism creates a physiological barrier to enrichment strategies that merits dedicated investigation. Nevertheless, organic Zn forms (e.g., amino acid chelates (Zn-AAs) and ZnMet) at 40-60 mg/kg approximately doubled milk Zn content, alongside reduced somatic cell counts (a key indicator of udder health and milk quality), and boosted both immunoglobulins (IgA and IgM) and antioxidant capacity (glutathione peroxidase and catalase) (Cai et al., 2021; Li et al., 2025). The superiority of these Zn sources in ruminants is likely due to their partial protection from being precipitated as insoluble sulfides or complexes in the rumen. Compared to traditional inorganic Zn, ZnO-NPs may represent a more effective source, as they may enhance mammary Zn translocation without compromising health (Cai et al., 2021). However, future studies should characterize ZnT2 and ZnT4 expression and regulatory capacity across dietary Zn sources, doses, and lactation stages as a prerequisite for designing effective Zn milk enrichment protocols.

In ***brief***, literature pointed out that Zn biofortification is a complex process. Dietary inorganic Zn forms are likely needed at high, often non-compliant, inclusion levels to force tissue deposition. In contrast, organic Zn sources (like ZnMet and ZnO-NPs) achieve relatively similar or superior deposition rates at lower, compliant doses by bypassing luminal antagonists (e.g., phytate) and utilizing alternative absorptive pathways. The physiological obstacle set by mammary ZnT transporters requires future studies to focus on characterizing transporter expression during lactation stages instead of just increasing dietary Zn levels. Ultimately, the paradigm of "more is better" is invalid for Zn, as further long-term safety, cost-benefit and environmental analyses are required, especially regarding the efficacy of novel organic and Nano-Zn sources across various species.

#### 3.2.3 Critical considerations related to zinc supplementation

**Fig. 7:**
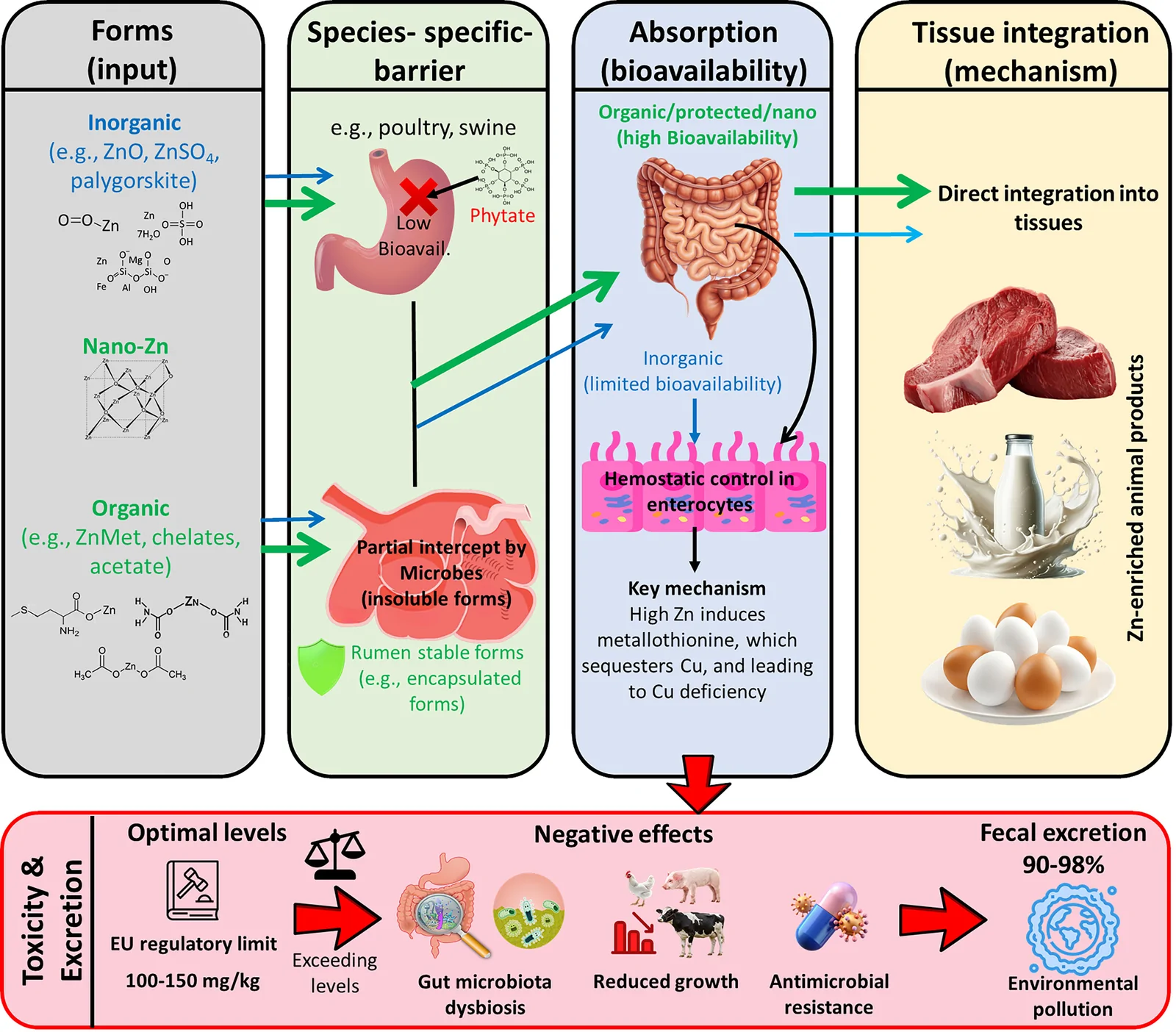
The homeostatic challenge of Zinc (Zn) enrichment in animal production.

Zn demonstrated efficacy in biofortifying animal products, with the superiority of organic sources over inorganic ones, especially in ruminants. However, the ruminal microflora can also convert Zn into insoluble complexes, making lipid-encapsulated Zn additives a preferred, yet understudied, strategy (Cai et al., 2021) (with major studies focused on health perspectives and not enrichment) to bypass microbial degradation. For improved Zn delivery, nanoencapsulation, using techniques like microencapsulation or spray freezing, has demonstrated greater encapsulation efficiency and smaller particle sizes, as well as enhanced performance and health of animals (Rao et al., 2023; Wang et al., 2009). However, further studies are needed to confirm its long-term biological and environmental safety. The difference in efficacy noticed among different sources emphasizes the necessity of species- and phase-specific Zn supplementation to optimize functional outcomes. Herein, the pursuit of Zn enrichment must also be carefully balanced against potential risks of antagonisms and toxicity (for an illustrative summary, see Fig. 7).

##### 3.2.3.1 Dietary interactions and antagonists

In a similar manner to Se, Zn absorption is affected by ***interactions*** with various dietary nutrients such as phytate, fibres, organic acids, animal protein sources and competitive minerals. Within the gastrointestinal tract, especially in non-ruminants, phytate (chemically hexaphosphates and pentaphosphates) binds Zn^2+^, forming complexes that hinder Zn uptake by intestinal enterocytes (Grases et al., 2004; Philippi et al., 2023), emphasizing the importance of maintaining a phytate/Zn ratio below 15 during formulation (Wang et al., 2022). Notably, high-fibre diets exacerbate this issue by accelerating gastrointestinal transit and often correlate with elevated phytate content (Humer et al., 2015). Compared with inorganic sources, organic sources biologically outperform inorganic sources with greater efficacy under high phytate and fibre contents (Barszcz et al., 2019), suggesting that the metabolism of a ZnMet complex differs from the metabolism of Zn from inorganic Zn sources. Relying on feedstuff low in phytate content directly decreases total dietary phytate, while strategies based on phytase (bacterial or fungal phytases), organic acids (citric/lactic acids), or vitamin B_3_ supplementation can hydrolyze phytate, liberate bound Zn (Zarghi et al., 2022), and enhance Zn absorption and tissue uptake (Akter et al., 2017). In addition to these strategies, the fermentation process as well reduces phytate content and other antinutritional components like tannins and oxalic acid, which improve Zn bioavailability compared to unfermented grains (Ahmed et al., 2020). The dietary protein is capable of modulating Zn absorption. Zinc, for example, can be more bioavailable when consumed with milk proteins (namely casein and whey proteins) and amino acids (like methionine and cysteine), in which derived casein phosphopeptides (formed during digestion) and whey osteopontin bind divalent Zn^2+^ to form less precipitated soluble complexes absorbed via ion channel and small peptide transport, while amino acids chelate to Zn and transport across intestinal membranes via specific amino acid transporters (Christensen et al., 2025; Feng et al., 2020; Hu et al., 2024; Li et al., 2019b; Miquel & Farré, 2007). These aspects are likely to confirm the bio-efficacy of Zn from organic sources. Minerals’ interactions further modulate Zn bioavailability: copper, iron, calcium, magnesium, and manganese compete via regulating metallothionein production in enterocytes (Cousins & Liuzzi, 2018; Gajula et al., 2011; Mendoza et al., 2004) and/or through binding to mineral transporters such as DMT1 and Zrt-/Irt-like protein (ZIP) transporters. These interactions are more than exclusively hypothetical; they are major challenges to precision enrichment. Within the ZIP family, ZIP3-5, ZIP8, ZIP11 and ZIP14 regulate Zn uptake and are predominantly found in apical membranes but also exist in intracellular membranes (Cao et al., 2025; Rolić et al., 2025; Wu et al., 2022; Zhang et al., 2019). For example, regarding the differential patterns, ZIP4 (SLC39A4) serves as the main intestinal Zn importer at the apical membrane, while ZIP8 (SLC39A8) and ZIP14 (SLC39A14) function at basolateral membranes and are involved in systemic Zn distribution; in contrast, ZIP3, ZIP5, and ZIP11 operate predominantly at intracellular membranes to regulate organellar Zn homeostasis. In a contrasting manner, Zn transporters (like ZnT1 and ZnT10, encoded by SLC30A genes) reduce cytoplasmic Zn by exporting it into the extracellular space or sequestering it in intracellular vesicles. Notably, ZnT1 is known as the primary basolateral exporter in enterocytes. Elevated dietary Zn may indirectly reduce iron absorption through competitive substrate sharing at ZIP4 (predominantly) and at DMT1, as well as through induction of metallothionein, which sequesters both metals in enterocytes, reducing their basolateral transfer (Cousins & Liuzzi, 2018). DMT1 is primarily located in apical membranes of duodenal enterocytes, where it acts as a transporter for divalent metal ions (Fe²⁺, Zn²⁺, Mn²⁺, and Cu²⁺) and is primarily regulated by Fe²⁺ status via iron-regulatory proteins (IRP1/IRP2) and hypoxia-inducible factor HIF-2α (Gunshin et al., 1997; Mastrogiannaki et al., 2009). These interactions underscore the importance of monitoring Cu²⁺ and Fe²⁺ status when implementing high-Zn supplementation strategies. Within this context, balancing minerals’ levels in the diet is critical for optimal Zn absorption and overall health, which calls for species- and stage-specific dietary strategies to optimize Zn utilization and mitigate antagonisms. Nevertheless, the impact of minerals on reducing Zn bioavailability is not fully comprehended, according to the aforementioned scientific evidence. Thus, future studies on these interactions are of importance.

Even though organic sources of Zn are recognized to have high bioavailability, their mechanisms and relative effectiveness, especially as related to health, are ***not yet fully understood***. As an example, the increased growth observed with ZnMet may be partly due to the availability of methionine (which is an essential amino acid), making it challenging to derive unambiguously concerning how efficiently Zn serves and warranting additional investigations.

##### 3.2.3.2 Potential health risks, regulatory guidelines and environmental impact

Zn is an essential trace element involved in numerous biological functions in livestock, including enzyme activity, the immune response, and growth. However, excessive Zn intake may still cause trace element imbalances by interfering with absorption (competitive bioavailability), emphasizing the necessity of monitored, species-specific Zn strategies. Zn is widely used as an anti-stress element and immune booster (Baumhover et al., 2025). However, ***excessive Zn supplementation***, which is often used for growth promotion or disease prophylaxis, can compromise animal performance, manifesting as reduced growth rates and defective feed conversion efficiency. Mechanistically, it has also been reported that the non-specific binding of Zn to peptides may trigger uncontrolled biological effects (enzyme inhibition (e.g., dipeptidyl peptidase III (DPP III) and pharmacokinetic modulation) that may further compromise animal metrics (Brugger & Windisch, 2017; Tomić et al., 2021).

Studies over the past decade have highlighted several ***risks associated*** with high levels of Zn supplementation in species like poultry, pigs, and cattle. In pigs, prolonged high-dose ZnO supplementation has been linked to gut microbiota disruption (Vahjen et al., 2011), drives the development of antimicrobial resistance (Hölzel et al., 2012), and causes Zn accumulation in various tissues (Sales, 2013). For poultry, excess Zn can impair mineral balance, reduce feed efficiency, cause the development of pathological modifications within organs (Hill & Shannon, 2019), and alter the gut microbiome in a manner that potentially compromises immunocompetence (Broom & Kogut, 2018). In ruminants, excessive Zn intake negatively interferes (competitive relationship) with copper absorption, resulting in deficiencies and potential immune dysfunction (Hill & Shannon, 2019). From another perspective, in humans, maternal Zn intake is positively correlated with breast milk Zn content. Nevertheless, excessive Zn intake (from supplements or heavily fortified products) can impair copper absorption in infants, culminating in reduced immune function and development of neurological disorders (Aumeistere et al., 2018). Therefore, it is important and critical to monitor/determine high doses of Zn in animal-derived products and human health, with the aim of delivering a balanced diet and promoting safety and sustainable practices. Moreover, collectively, these findings underscore the necessity for balanced Zn supplementation to optimize benefits while mitigating risks across species.

Reducing Zn supplementation to physiologically ***appropriate levels*** and adopting organic/chelated Zn sources are key strategies to mitigate the risks of toxicity and environmental consequences. In this regard, the latest European Union legislation (EFSA, 2014) has substantially restricted the previously permissible high Zn doses in complete livestock diets by establishing newly proposed limits of 150 mg/kg (piglets, sows, rabbits, salmonids, cats, and dogs); 120 mg/kg (turkeys for fattening); and 100 mg/kg (all other species and categories). Within this context, it is notable that some of the hereinabove-reviewed studies employed dietary doses exceeding these regulatory limits (e.g., > 100 mg/kg in duck complete feed) without reporting adverse effects, although potential long-term risks remain unassessed. Pharmacological Zn doses (e.g., 2000–3000 mg/kg feed ZnO, equivalent to 100 mg/kg body weight in swine) have been used transiently for diarrhoea control in some regions (Duffy et al., 2023; Liu et al., 2023b; Pei et al., 2019; Poulsen, 1995). Regarding this issue, Zn acts as a supporter for intestinal barrier function (tight junction), a booster for immune response (innate and adaptive immunity), an accelerator for the mucosal healing process (proliferation and repair), and a mitigator of inflammatory responses. However, the European Union’s legislation banned such excessive ZnO concentrations in veterinary medications as of 2022 (EMA, 2022), citing related ecological and public health concerns.

Regarding ***environmental impact***, the Zn effect largely relies on excretion patterns: 2–10% of Zn is eliminated renally, while 90–98% is excreted fecally, posing ecological risks (contributing to soil/water contamination) and raising concerns about sustainable livestock production and the environment (Hölzel et al., 2012). Resealed Zn can modify the soil microbial profile and enzymes’ activities, consequently modulating the decomposition of biomass and the cycling of nutrients (Du et al., 2011; Ge et al., 2011; Rajput et al., 2018). The environmental burden represents the primary homeostatic mechanism for Zn regulation, which remains operational as long as Zn intake is sustained (EFSA, 2014; Mir et al., 2020). Hence, the urgency of adhering to established guidelines (EFSA, 2014) is aimed at reducing Zn’s ecological footprint by about 20%, while coincidentally enhancing animal performance and consumer health, as well as curbing the progress of antibiotic resistance linked to excessive Zn use. This situation creates a critical paradox for enrichment, in which enriching an animal diet with Zn to overcome homeostatic barriers for product fortification directly conflicts with environmental sustainability mandates, making high-bioavailability/low-dose organic forms the only viable path forward.

Beyond Zn enrichment in tissues, particular attention has to be devoted to the environmental sustainability of functional feed supplements. The fortification of products with supplement(s) (like Se, Zn and n-3 PUFAs) is commonly known to improve human health; however, it inherently shifts resource use and excretion patterns. For example, the reliance on marine oils (from fish or algae sources) for elevating C22:6n3 (docosahexaenoic acid, or DHA) concentration in products would most likely introduce a resource trade-off, as overreliance on oils derived from fish depletes marine ecosystems and competes with oceanic biodiversity, whereas large-scale marine algae cultivation requires substantial inputs (e.g., energy, water and land) for fermentation or photobioreactors. In addition, high-dose mineral strategies to overcome homeostatic barriers (specifically in Zn and unprotected Se cases) typically lead to about 70-95% of ingested minerals being excreted in manure, consequently contributing to soil heavy accumulation, flushing into aquatic systems and distortion of soils’ microbiomes. Considering supplement form variability, precision strategies utilize highly bioavailable organic or rumen-protected forms to reduce this footprint, mainly by achieving target tissue deposition at supplementation levels, thereby diminishing the environmental excretion burden, which is a vital consideration for sustainable livestock farming.

##### 3.2.3.3 Effects of excessive levels on the gut microbiota and mineral balance

Dietary Zn supplementation has historically been employed in animal nutrition, especially in swine and fowl, for its growth-enhancing and antimicrobial effects. Nevertheless, emerging research outcomes raise ***concerns*** regarding its effects on the gastrointestinal microbiota composition, nutrient absorption, and long-term mineral homeostasis. These potential ramifications of Zn on gastrointestinal microbiota have already been reported primarily in livestock species like swine, poultry, and cattle, as well as they extend to horses, which exhibit heightened sensitivity to elevated Zn exposure.

In ***pigs***, high Zn levels induce intestinal microbiota dysbiosis, favouring the proliferation of Zn-tolerant pathogens (e.g., *Escherichia coli*) whilst diminishing beneficial taxa (like *Lactobacillus*) (Bednorz et al., 2013; Vahjen et al., 2011). Furthermore, prolonged Zn supplementation disrupts mineral absorption kinetics in pigs through competitive interactions with intestinal transporter sites, notably reducing copper and iron uptake (Cousins & Liuzzi, 2018). For ***poultry***, elevated Zn levels (> 100 mg/kg diet) exert both immunomodulatory and growth-promoting effects (Star et al., 2012) but risk destabilizing the intestinal microbial ecosystem, accordingly impairing overall performance whenever the microbial balance is compromised (Broom & Kogut, 2018; Skalny et al., 2021). Excessive Zn supplementation also disrupts phosphorus and calcium metabolism, potentially hindering bone development in broilers (Hejna et al., 2018). In ***dairy cows***, the focus has been more on the bioavailability and structure/form of Zn rather than pharmacological doses. Organic Zn sources, under no excessive supplement, positively influence rumen microbial activity and improve immune responses without the adverse effects of high-dose inorganic Zn (Wang et al., 2013). The exact impact of Zn on the rumen microbiota during the early life phase has not been fully defined, and this uncertainty calls for further studies to optimize strategies for optimal rumen function and overall animal development (Hejna et al., 2018).

While ***high Zn supplementation*** offers short-term benefits like immediate growth-promoting and therapeutic effects, especially in young pigs and poultry, it coincidentally destabilizes gut microbial homeostasis and disrupts systemic trace mineral balance. To maximize efficacy while minimizing metabolic and microbial risks, Zn optimization must prioritize species-specific physiology, dosage precision, and Zn-source selection. For example, organic Zn, with its superior bioavailability, achieves desired zootechnical and health outcomes at notably lower dietary doses (40–100 mg/kg), thereby reducing the environmental impact of excess excretion. Consequently, the pursuit of Zn biofortification must abandon the outdated paradigm of pharmacological overdosing. Regardless, keeping research priorities is critical, especially considering how different Zn sources specifically influence the gut microbiome in various species.

##### 3.2.3.4 Zinc section key points summary

The homeostatic regulation is a critical factor challenging Zn biofortification, with organic sources and nano-forms being more favourable due to their strong potential in overcoming intestinal barriers. However, the excessive Zn supplementation, especially inorganic forms, is strongly not recommended, as it can disrupt the ruminal microbial ecosystem, induce systemic mineral antagonism, and raise serious environmental implications. The literature main gap is observed in the insufficient mechanism data related to ZnT transporter regulation to bypass the physiological ceiling in milk enrichment. In addition, further investigations are recommended, especially those related to long-term safety and microbial impact of novel forms, aimed at establishing a commercial scale. Moreover, the environmental burden of fecal Zn excretion demands life-cycle assessments for all enrichment strategies.

### 3.3 Vitamin E (Vit E)

Vit E, which exists in multiple forms (tocopherols, tocotrienols), is one of the primary fat-soluble vitamins and is not endogenously synthesized in livestock animals. Its major biological relevance is commonly recognized for its potent antioxidant features, portraying a fundamental chain-breaking antioxidant that is not replicated by other endogenous antioxidants, which protects cellular membranes and biomolecules (especially lipids like PUFAs) from free radicals. In this respect, unlike enzyme-based systems (e.g., glutathione peroxidase), the tocopherol structure of Vit E is the central defender against lipid peroxidation in membranes, where it intercepts lipid peroxyl radicals (LOO•) by donating a hydrogen atom, thereby generating a relatively stable tocopheroxyl radical (α-Toc•) and terminating the propagation chain, ultimately halting the detrimental chain reaction of lipid peroxidation, which ends up in rancidity and cell damage. However, the tocopheroxyl radical must be regenerated to active tocopherol, principally by ascorbate (Vit C) at the membrane-water interface or through glutathione-dependent systems supported by Se, to avoid its own pro-oxidant activity under conditions of high oxidative load (Sies & Stahl, 1995; Traber & Stevens, 2011). Ascorbate is itself regenerated by the glutathione (GSH)/glutathione reductase system. Concurrently, Se-dependent glutathione peroxidase (GPx) reduces the hydroperoxide product (LOOH) to non-reactive alcohols, preventing secondary radical generation that would otherwise deplete Vit E. This mechanistic interdependence is the biochemical rationale for the synergistic supplementation of Vit E with Se and Vit C. In addition, Vit E is an imperative component for metabolism and is involved in immunomodulation, embryogenesis, nucleic acid metabolism, and maintenance of the structure and function of tissues (Shastak et al., 2023). On these bases, Vit E supplementation has demonstrated diverse benefits (demonstrating its critical importance) across various livestock sectors, including dairy, swine and poultry sectors. For instance, Vit E has been reported to mitigate the adverse effects of subacute ruminal acidosis (SARA), resulting in increased rumen fermentation, high milk yield and decreased mastitis incidence in dairy cows (Chandra et al., 2013). In swine, this vitamin has been shown to enhance meat quality, reduce stress, and improve growth rates (Lu et al., 2014a, 2014b). For poultry, it has been demonstrated to boost egg production, the hatchability rate, and overall growth (Surai et al., 2016). On the contrary, Vit E deficiency is commonly associated with various health implications, such as immunosuppression, lower fertility and a high embryo mortality rate.

#### 3.3.1 Major vitamin E forms and their bioavailability

**Fig. 8:**
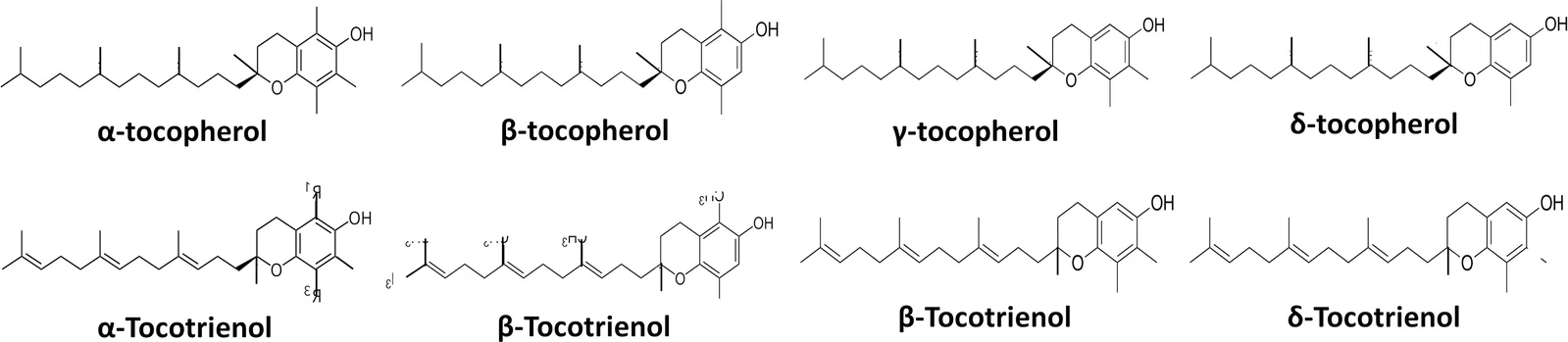
Structure of vitamin E isomers.

Vit E is mainly found in two forms: tocopherols (saturated phytyl tail) and tocotrienols (unsaturated isoprenoid tail with three double bonds) (see Fig. 8), with each form existing in 4 isomers (α, β, γ, and δ) and 8 stereoisomers, including both 2R and 2S forms (e.g., RRR, RRS, RSS, SSS, RSR, SRS, SRR and SSR-αtocopherol) (Fu et al., 2017; Razali et al., 2025). The following figure (Fig. 8) illustrates the structure of Vit E isomers.

Unlike Se (which, among the inorganic forms, must first be integrated into an amino acid) or Zn (which binds to proteins as a cofactor), Vit E is a fat-soluble molecule that remains chemically independent as it moves through the body. However, dietary Vit E absorption (approx. 10–70%, contingent upon dietary fat content, bile salt availability, esterase activity (for esterified forms), and mucosal health status) and effectiveness are greatly influenced by its source together with dietary matrix. Tocopherols have high biological activity, with the RRR configuration (*all-rac-*) of α-tocopherol (or D-α-tocopherol) being the most predominant form in animal tissues and their derived products (Borel et al., 2013; Brigelius-Flohé, 2006; Kiyose, 2021; Zingg, 2007), explaining why it is a research focus compared to other forms. The synthetic *all-rac*-α-tocopherol is an equimolar mixture of all 8 possible stereoisomers at the three chiral centres (C-2, C-4′, and C-8′), of which only RRR-α-tocopherol (approx. 12.5% of the mixture) is structurally identical to the natural form. The remaining 7 stereoisomers are discriminated against by hepatic α-TTP and also are, with greater speed, metabolized and excreted through the ω-oxidation/β-oxidation route mediated by cytochrome P450, resulting in the approximately 50% lower biopotency of synthetic relative to natural α-tocopherol (Dersjant-Li & Peisker, 2009; Leal et al., 2019). This pattern is an outcome of the fact that the hepatic α-tocopherol transfer protein (α-TTP) discriminatorily integrates the natural form into lipoproteins (very low-density lipoproteins) before distributing them to tissues, demonstrating a selective retention mechanism. Outstandingly, α-TTP binds to α-tocopherol with a 100% affinity, β-tocopherol with a 38% affinity, γ-tocopherol with a 9% affinity, and δ-tocopherol with a 2% affinity (Hosomi et al., 1997; Szewczyk et al., 2021). Among tocopherols, α-tocopherol stereoisomers are the most often utilized forms (as powerful antioxidants even at lower doses) by farm animals, ranking as the second-most important vitamin in terms of quantity consumed globally (Scheideler et al., 2010; Sobotka et al., 2012). On the other hand, α-tocotrienol efficacy over α-tocopherol has been reported regarding antioxidant properties and neuroprotection (Peh et al., 2016; Rafique et al., 2024), but they are less abundant naturally and have lower bioavailability, although efficient deposition has been observed in the brain, liver and skin (Razali et al., 2025). Therefore, despite tocotrienols also being recognized for their positive impact on animal health, their use as animal feed supplements is less well-recognized. In contrast, the transfer of dietary α-tocopherol to animal tissues/products is well-acknowledged (it stems from the hepatic selection of this active form) (Idamokoro et al., 2020); therefore, it has been commonly used by animal nutritionists to stimulate the production of antioxidant-rich animal products such as Vit E-enriched meat (Chauhan et al., 2014), milk (Fauteux et al., 2016), and eggs (Scheideler et al., 2010).

#### 3.3.2 Effects of dietary vitamin E on meat, egg and milk α-tocopherol levels

Dietary Vit E is effectively transferred to meat, eggs, and milk, with deposition levels being largely dose-dependent, with efficacy diverging sharply between monogastrics (direct intestinal absorption) and ruminants (risk of ruminal degradation). It is also noted that the majority of available studies within the last 15 years have focused on the poultry sector, while there are a few studies on swine and ruminants.

Across livestock species, high doses (50–200 mg/kg feed for ***swine and poultry***, while 300 IU/kg for ruminants) typically elevate α-tocopherol concentration in tissues. Concerning meat from monogastric animals, specifically from swine and broilers, dietary Vit E supplementation (100–200 mg/kg diet for pigs; 200 mg/kg feed for broilers; and 40–80 mg/kg feed for geese) significantly (P ≤ 0.05 and P ≤ 0.01, respectively) elevated tissue α-tocopherol concentrations in pig’s *longissimus dorsi* muscle (Szterk et al., 2016; Sobotka et al., 2012), broiler chicken breast and leg muscles (Zdanowska-Sąsiadek et al., 2016), and goose breast (in linear relationship) (Yang et al., 2022) by more than 2-fold (see Table 2), with relatively similar accumulation rates across pigs and broilers. Moreover, Sztrek et al. (2016) observed higher Vit E deposition in hybrid swine breeds compared to other breeds (P ≤ 0.05). The literature also confirmed the biopotency hierarchy, in which the deposition from natural D-α-tocopherol and natural RRR-α-tocopherol was found to be superior to that from synthetic DL-α-tocopherol acetate and all-rac-α-tocopherol (though all forms increased tocopherols in tissues), as demonstrated in broiler chicken breast muscle (P ≤ 0.05) by Voljč et al. (2011), Arshad et al. (2013) and Cheng et al. (2016). These studies highlight the importance of source bioavailability in enrichment strategies, as well as the hepatic α-TTP selective retention mechanism. The stereoisomer-selective transfer of α-tocopherol from diet to animal products is a well-known event. A discrimination similarly demonstrated by Peisker et al. (2014) in egg yolk and hatched chick tissue, reporting direct stereoisomer-level evidence that tissue stereoisomer composition closely reflected the dietary composition (also confirmed in broiler breast and thigh by Tomažin et al. (2013)), with 2R α-tocopherol isomers from all-rac-α-tocopheryl acetate being markedly more retained than 2S forms, depicting dose-dependent and tissue-specific patterns (brain most selective). Of note, this study also reported that this discrimination was more pronounced in swine and ruminants compared to laying hens, indicating α‑TTP is less selective for eggs than other tissues. However, Pitargue et al. (2019) reported that regardless of the sources of Vit E, increasing the dosage or supplementation level of Vit E in broiler diets had a corresponding linear effect (P ≤ 0.01) on the α-tocopherol content in breast meat. Crucially, the ultimate functional value of this deposition is most apparent when highly unsaturated diets are offered to animals. According to the study of Bernardi et al. (2022), a high degree of unsaturated diet (supplemented with 3% linseed oil) increased tissues’ unsaturation levels and compromised oxidative stability, whereas the concurrent supplementation of Vit E at 200 mg/kg diet resolved the issue of backfat oxidative stability and sensory degradation during storage, outperforming natural plant extracts. With respect to monogastrics, specifically pigs, Vit E efficacy is likely dose-dependent, improving n-3 FA profiles (Huang et al., 2020). Comparable findings were reported in ***ruminants***, specifically fattening lambs, wherein 1000 mg Vit E alongside sunflower seeds markedly increased Vit E content in the *longissimus lumborum* muscle and decreased lipid oxidation, confirming the importance of Vit E in oxidative stabilization (De Almeida et al., 2015). At the transcriptomic level, González-Calvo et al. (2017) demonstrated that 500 mg dl-α-tocopheryl acetate/kg feed altered the expression in the lamb *longissimus thoracis* (suppressed genes related to intracellular signalling cascades) and subcutaneous fat (upregulated genes involved in lipid biosynthesis, cholesterol and sterol biosynthesis, while downregulated stress-response genes), providing the first genome‑wide evidence that Vit E regulates ruminant meat lipids transcriptionally, not just via antioxidation. In beef, this protection extends to colour stability, as when the meat redness decreased due to enriched extruded linseed, 2500 IU Vit E co-supplementation protected the subcutaneous lipids from oxidation and preserved meat colour (Morittu et al., 2021). Interestingly, the oil matrix itself can modulate Vit E deposition, as such effects have been reported in quail fed Echium oil (Salahi Kojur et al., 2026), wherein concentrations of Vit E and carotenoids were remarkably increased.

**Table 2:** A summary of zinc and vitamin E sources, inclusion rates in various studies, improved animal products, and effects achieved.

**Sources**	**Levels of application and inclusion rates**	**Targeted animal product**	**Functional value effect on animal product**	**Authors**
ZnSO_4_.7H_2_O Zn-amino acid chelate Zn proteinate	3 levels: 60, 120, and 180 mg/kg feed 3 levels: 60, 120, and 180 mg ZnSO_4_/kg feed 3 levels: 60, 120, and 180 mg ZnSO_4_/kg feed	Broiler chicken breast, thigh and liver	dietary Zn sources increased Zn content in liver, breast, and thigh muscles (P ≤ 0.01). No difference recorded among the different sources.	(Liu et al., 2015b)
ZnPal	5 levels: 0, 20, 40, 60, and 80 mg/kg feed	Broiler chicken breast muscle	Zn concentration was linearly increased in the *pectoralis major muscle* (P = 0.05). 20 mg Zn/kg feed was statistically sufficient to stimulate Zn accumulation in the muscles.	(Yang et al., 2016)
ZnSO_4_·7H_2_O	6 levels: 0, 15, 30, 60, 120, 240 mg/kg feed	Duck breast muscle and liver	linearly increased with increasing inclusion of Zn (P ≤ 0.05). Based on the linear regression model, 91.32 mg Zn/kg feed was suggested to be the optimal dosage	(Wen et al., 2019)
ZnSO_4_·7H_2_O	levels for phase 1 (1 to 21 days): 0, 20, 40, 60, 80, 100, or 120 mg/kg feed levels for phase 2: (22 to 42 days): 0, 16, 32, 48, 64, 80, or 96 mg/kg feed	Broiler chicken breast muscle	highest Zn retention occurred in the breast muscle of the growers with 80 mg Zn/kg (P ≤ 0.05)	(Zhang et al., 2018b)
ZnSO_4_.7H_2_O ZnMet	4 levels: of 0, 50, 100 and 150 mg Zn/kg feed 4 levels: of 0, 50, 100 and 150 mg Zn/kg feed	Egg	increasing levels of inorganic or organic Zn significantly (P ≤ 0.05) enhanced egg Zn	(Bahakaim et al., 2014)
ZnO-NPs	3 levels: 40, 80, and 120 mg Zn/kg feed	Egg	linear increase of Zn in egg according to the inclusion levels	(Abedini et al., 2018)
ZnSO_4_.7H_2_O Zn amino acid complex	2 levels: 35 and 70 mg/kg feed 2 levels: 35 and 70 mg/kg feed	Egg	Zn content in eggs increased linearly with supplementary Zn: R2 = 0.363 with P = 0.008 for organic Zn, while R2 = 0.366 with P = 0.008 for inorganic Zn	(Yu et al., 2020)
ZnSO_4_.7H_2_O	5 levels: 10, 20, 40, 80, and 160 mg/kg feed	Duck egg	dietary additive increased Zn deposition in egg yolk (quadratic relationship). Optimal Zn supplementation was estimated between ∼65–95 mg/kg	(Zhang et al., 2020b)
ZnO ZnMet ZnThr	2 levels: 30, 60 and 90 mg/kg feed 2 levels: 30, 60 and 90 mg/kg feed 2 levels: 30, 60 and 90 mg/kg feed	Egg	Low organic Zn sources showed greater efficacy in maintaining performance and improving egg quality, as well as increasing bioavailability compared to dietary ZnO (P ≤ 0.05)	(Behjatian Esfahani et al., 2021)
Zn proteinate	4 levels: 40, 80, 120, and 160 mg/kg feed	Egg	Zn supplementation increased egg Zn content (P ≤ 0.05)	Kannan et al., (2022)
ZnO+ Fe	6 levels: 100, 200, 300 mg (1:1 ratio)/kg feed	Egg	significant increase in Zn and Fe content in egg yolk at 300 mg/kg. The cost per egg produced in response to different levels of Fe and Zn supplementation was not affected.	(Ullah et al., 2024)
ZnO-NPs ZeMet	1 level: 40 mg/kg DM I level: 40 mg/kg DM	Bovine milk	Zn content of milk was markedly elevated with 0.15 to 0.5% additional transfer rate	(Cai et al., 2021)
**Studies focus on Vit E**				
DL-α-tocopheryl acetate	20 vs 200 mg/kg diet	Broiler chicken meat	high inclusion rate increased α-tocopherol in muscle (P ≤ 0.001), reduced lipid peroxidation, and improved performance under stress	(Gao et al., 2010)
natural RRR-rac-α-tocopherol all-rac-α-tocopherol	1 level: 85 IU /kg feed 2 levels: 85 IU and 200 IU/kg feed	Broiler chicken breast	both forms increased tissue α-tocopherol levels. Bioactivity ratio of RRR:all-rac ≈ 1.39	(Voljč et al., 2011)
DL-α-tocopherol acetate	100 mg/kg feed	Pork	α-tocopherol concentration significantly (P ≤ 0.05) increased by more than 2-fold	(Sobotka et al., 2012)
natural D-α-tocopherol DL-α-tocopherol acetate	1 level: 200 mg/kg feed 1 level: 200 mg/kg feed	Broiler chicken breast and thigh	both additive increased α-tocopherol deposition in meat, with the natural form showing higher efficacy (P ≤ 0.05). Supplementation improved antioxidant status and lipid stability	(Arshad et al., 2013)
RRR-α-tocopherol γ-tocopherol; Natural α-toc.+ γ-toc.	1 level: 67 mg/kg feed 1 level: 67 mg/kg feed 1 level: 35.5 mg α-toc+ 35.5 γ-toc/kg feed	Broiler chicken breast and thigh	Dietary isomers markedly increased their related isomer in breast and thigh (P ≤ 0.001), as well as improved oxidative stability	(Tomažin et al., 2013)
DL-α-tocopherol acetate	1 level: 1000 mg Vit E/kg DM	Mutton	dietary Vit E increased (P ≤ 0.05) the level Vit E in the meat while decreasing lipid oxidation.	(De Almeida et al., 2015)
DL-α-tocopherol acetate	1 level: 500 mg/kg concentrate (≈500 mg/kg feed)	Mutton (*Longissimus thoracis*)	muscle α tocopherol significantly (P ≤ 0.001) reached 0.61-0.90 mg/kg fresh meat	(González Calvo et al., 2015)
DL-α-tocopherol acetate natural D-α-tocopherol	1 level: 20 IU /kg feed 1 level: 20 IU/kg feed	Broiler chicken breast and thigh	α-tocopherol concentration was enhanced in both tissues by Vit E supplementation, whereas this effect was significant in the natural D-α-tocopherol group (p ≤ 0.05)	(Cheng et al., 2016)
D-α-tocopherol	2 levels: 100 and 200 mg/kg feed (α tocopherol)	Pork	Vit E levels increased from 1.7 to 4.9 mg/kg meat. Hybrid breed showed higher deposition (P ≤ 0.05)	(Szterk et al., 2016)
DL-α-tocopherol acetate	1 level: 200 mg/kg feed	Broiler chicken breast	200 mg/kg increased α-tocopherol level (3-fold) in breast muscles with (P ≤ 0.01)	(Zdanowska-Sąsiadek et al., 2016)
DL-α-tocopherol acetate	500 mg/kg concentrate	Mutton (*Longissimus thoracis*)	significantly (P ≤ 0.001) increased muscle α tocopherol content; down regulated genes related to stress response; improved meat quality	(González-Calvo et al., 2017)
D-α-tocopherol with or without Se and Vit C	200 IU/kg feed	Broiler chicken breast	increased α-tocopherol concentration in breast muscle (P ≤ 0.0001). Improved oxidative stability of fresh, frozen, and cooked meat. Co-supplementation with Vit C and Se prevented meat oxidation but tocopherol levels in meat were indifferent.	(Leskovec et al., 2019)
DL-α-tocopherol acetate	2 levels: 40, and 80 mg/kg feed	Goose breast	40-80 mg/kg increased α-tocopherol retention in breast (P ≤ 0.05). In addition, it reduced PUFA oxidation and increased n-3 PUFAs	(Yang et al., 2022)
D-α-tocopherol BHT	3 levels: 50, 100, 150 IU/kg feed 3 levels: 50, 100, 150 IU/kg feed	n-3-enriched eggs	eggs from flax+ α tocopherol had 4.5- to 12- fold higher α tocopherol. Regarding oxidative stability, Vit E was more effective than BHT at day 0 of storage, but none of them was effective with time	(Hayat et al., 2010)
natural D-α-tocopherol	3 levels: 50, 100, or 150 IU/kg feed	Egg	α-tocopherol in the yolk increased linearly in significant amounts for all dosages (P ≤ 0.05)	(Scheideler et al., 2010)
DL-α-tocopherol acetate	1 level: 200 mg/kg feed	n-3 PUFA-enriched eggs	significantly increased α-tocopherol content in eggs (P ≤ 0.05). It also reduced lipid hydroperoxides	(Botsoglou et al., 2013)
DL-α-tocopherol acetate	2 levels: 150 or 300 IU /kg feed	Egg	Vit E deposition increased from 12.78 to 42.28 IU/100g yolk. Results also showed higher egg production	(Gjorgovska et al., 2013)
natural RRR-Rac-α-tocopherol acetate all-rac-α-tocopherol acetate	2 levels: 30 and 60 mg/kg feed 2 levels: 30 and 60 mg/kg feed	Egg	both forms markedly increased average α-tocopherol content in yolk, with further substantial efficacy for all-rac form (P ≤ 0.05). 2R stereoisomers preferentially transferred to yolk; brain most selective for RRR isomer	(Peisker et al., 2014)
Synthetic (assumed)	1 level: 60 mg/kg diet	Egg	markedly increased Vit E content in eggs, and reduced egg cholesterol (P ≤ 0.05)	(Santoso et al., 2017)
natural D-α-tocopherol	4 levels: 0, 25, 50, 75, and 100 mg/kg DM	Egg	α-tocopherol in the egg yolk increased quadratically and linearly with increasing levels of natural D-α-tocopherol (P ≤ 0.05) 100 mg/kg inclusion level gave the best outcome	(Zhao et al., 2021a)
all-rac-alpha-tocopheryl acetate	2 levels: 25.2 and 125.2 mg/kg feed (part of multi nutrient enrichment)	Egg	Vit E content was 2.74-fold higher in enriched eggs vs. control (P ≤ 0.001). Se and lutein also increased	(Kralik et al., 2023)
natural D-α-tocopherol	2 levels: 100 or 300 mg/kg feed (+ fish oil)	PUFA-enriched egg	Vit E content in yolk increased to 3.6-fold (100 mg/kg feed) and 14.5-fold (300 mg/kg feed)	(Nadia et al., 2023)
Vit premix	4 levels: 100%, 75%, 50%, and 0% of Vit. premix at 0.1% feed	Egg	α-tocopherol concentration in eggs was significantly higher in control (100% premix) vs. 0% group	(Choi et al., 2025)
DL-α-tocopherol acetate	1 level: 1600 mg/day	Bovine milk	dietary supplementation increased milk α-tocopherol from 0.77 to 1.05 mg/kg, while no effect found on fatty acids	(Höjer et al., 2012)
DL-α-tocopherol acetate	300 IU Vit E/kg DM	Bovine milk	α-tocopherol concentration in milk and its fat content significantly increased (P ≤ 0.05)	(Fauteux et al., 2016)
Vit E (non-specified)	300 IU Vit E/kg feed (+ flaxseed meal)	Bovine milk	milk tocopherol increased 3.4-fold vs. control; however, oxidative stability not improved significantly	(Rico et al., 2021)

Abbreviations: BHT, butylated hydroxytoluene; DM, dry matter; Fe, iron; n-, omega-3 fatty acids; PUFA, polyunsaturated fatty acids; ZnMet, zinc methionine; ZnO, zinc oxide; ZnO-NPs, zinc oxide nanoparticles; ZnPal, Zn-bearing palygorskite; ZnSO_4_.7H_2_O, zinc sulfate heptahydrate; ZnThr, zinc threonine.

It appears that one of the most key findings of Vit E enrichment strategies is its synergistic dependency during the storage period. This has been demonstrated in the breast meat of Ross 308 broilers, in which the co-supplementation of Vit E (200 mg/kg) with Se (0.2 mg/kg) and/or Vit C (250 mg/kg) substantially maintained its high (P ≤ 0.05) α-tocopherol level during chilling and freezing processes compared to the meat from the group offered Vit E alone (Pečjak et al., 2022). Hence, this study underscores the gained value of using the dietary antioxidant mixtures (like Vit E, Vit C, and Se) in enhancing the antioxidant value of broiler breast meat, a functional synergy further demonstrated by Leskovec et al. (2018, 2019) in broilers fed an n-3-enriched diet, where the combination of α-tocopherol, ascorbic acid, and Se increased blood GPx activity and prevented meat (fresh, cooked and frozen) from oxidation during storage, as compared to α-tocopherol alone, although both groups were significantly indifferent regarding α- and γ-tocopherol levels in meat. In addition, neither sole Vit C nor Se altered meat quality attributes, proposing that, at supranational levels, Vit E is the primary driver of oxidative stability in broiler meat. Nevertheless, its co-supplementation with Vit E, and Se helps in maintaining its level, an effect described as the sparing effect. At the biological level, this “sparing effect” is majorly driven by Se-dependent glutathione peroxidase and Vit C reducing the tocopheroxyl radicals, thereby recycling active Vit E. With respect to this point, an extensive discussion has been provided in Section 3.3.3.2.

All available ***egg*** studies consistently demonstrated that Vit E supplementation, regardless of form (natural D-α-tocopherol, RRR-α-tocopherol, DL-α-tocopherol acetate, synthetic, or vitamin premix) and regardless of egg type (regular or n-3/PUFA-enriched), increased α-tocopherol content in eggs (e.g., Choi et al., 2025; Nadia et al., 2023; Peisker et al., 2014; Santoso et al., 2017). Furthermore, the linear relationship between supplemented dietary natural D-α-tocopherol levels (0, 25, 50, 75, or 100 mg/kg DM) and deposited α-tocopherol concentrations in eggs’ yolks was demonstrated, whereby the highest inclusion rate is associated with the best deposition outcome (Zhao et al., 2021a). A dose-dependent relationship was likewise reported earlier by Gjorgovska et al. (2013), who found that α-tocopherol deposition in yolk (12.78, 21.34, and 42.28 IU/100 g yolk) elevated linearly with dietary Vit E (0, 150, and 300 mg/kg feed, respectively); however, with declining marginal benefits at the maximum inclusion level, suggesting a practical threshold for yolk enrichment. The potential synergistic benefit provided through the dietary Vit E and Se combination has been investigated. When both Vit E (50, 100, or 150 IU/kg) and Se (0.55 or 0.75 mg/kg diet) were supplemented concurrently in the layer diet, Se and α-tocopherol levels were markedly higher in egg yolk from groups fed on diets with the highest inclusion rates (Scheideler et al., 2010). Moreover, while the synergistic relationship occurred at the deposition and functional level in meat, egg demonstrated a somewhat similar relationship at both levels, showing high α-tocopherol level and low lipid hydroperoxides in n-3 PUFA-enriched eggs (Botsoglou et al., 2013; Sobotka et al., 2012). However, it is crucial to distinguish between Vit E protective role and its impacts on lipid metabolism and deposition, wherein Vit E is most likely acting as a stabilizer rather than a modulator of lipid synthesis. For instance, Vit E supplementation (up to 300 mg/kg feed) to CLA-enriched layer diets successfully prevented the oxidation of highly unsaturated FAs; however, the egg yolk FA calculated indices remained unaltered (Franczyk-Żarów et al., 2019), confirming its role as a stabilizer. In addition, Hayat et al. (2010) showed comparable stabilization in n‑3 eggs, in which 50-150 IU/kg diet enhanced antioxidant capacity during storage, with egg α-tocopherol level markedly increased from dietary Vit E content compared to BHT, highlighting the dual nutritional and functional role of Vit E compared to synthetic antioxidants. The power of the combined multi-antioxidants has further been illustrated by Kralik et al. (2023), who demonstrated that the combined approach of soybean and linseed or marine oils, together with Se, Vit E and lutein, significantly increased PUFA levels, as well as Vit E (+ 2.74-fold) and lutein (+ 8.94-fold) contents compared to conventional eggs.

Dairy animals also demonstrated the transfer of dietary Vit E to their ***milk*** yield. For example, DL-α-tocopherol acetate at 1600 mg/day increased milk α-tocopherol from 0.77 to 1.05 mg/kg (Höjer et al., 2012). In addition, when lactating Holstein cows (224 ± 18 d in milk) were fed a soybean-based diet (CTR), a soybean+ 300 IU Vit E diet, or an alfalfa-based diet, the concentration of Vit E in milk and its fat content significantly increased compared to those in the CTR group, but this level was relatively similar to that recorded in the group fed alfalfa (Fauteux et al., 2016). However, the protective capacity of Vit E in milk is typically challenged with the increment of dietary highly unsaturated oil, such as the marine lipid challenge. Within this context, it is important to emphasize that the basal dietary Vit E level must be adequate against oxidation in moderate n-3 enriched products (Rico et al., 2021); however, a strong PUFA enrichment strategy likely represents a challenge, requiring high dietary Vit E inclusion levels. According to Bragaglio et al. (2015), dietary 2000 IU Vit E alongside DHA-rich microalgae remarkably boosted DHA levels in the milk of dairy cows as well as immune response; however, sensorial modifications were significant, as the DHA-enriched milk could still be discriminated from control milk via triangle testing.

In ***summary***, Vit E enrichment in animal products is largely predictable from a dose dependence perspective, while its efficacy or value is more economical rather than purely additive. However, dietary Vit E alone is biologically not very efficient and economically wasteful, especially for dietary strategies targeting meat products. The combined supplementation with Vit C and/or Se is likely advisable, preventing tocopheroxyl radicals from acting as pro-oxidants, which maximizes oxidative stability and, consequently, tissue preservation. In this respect, it is also necessary to scale Vit E with dietary unsaturation level, aiming at avoiding rapid oxidation and sensory rejection. On these bases, feeding strategies must formulate around the antioxidant network and the specific oxidative load, leveraging their effective synergistic function (Rooke et al., 2004). A summary of the above studies involving Vit E and other synergistic elements can be found in Table 2, offering health benefits to consumers and valuable insights for animal nutrition strategies.

#### 3.3.3 Key nutritional considerations concerning vitamin E supplementation

**Fig. 9:**
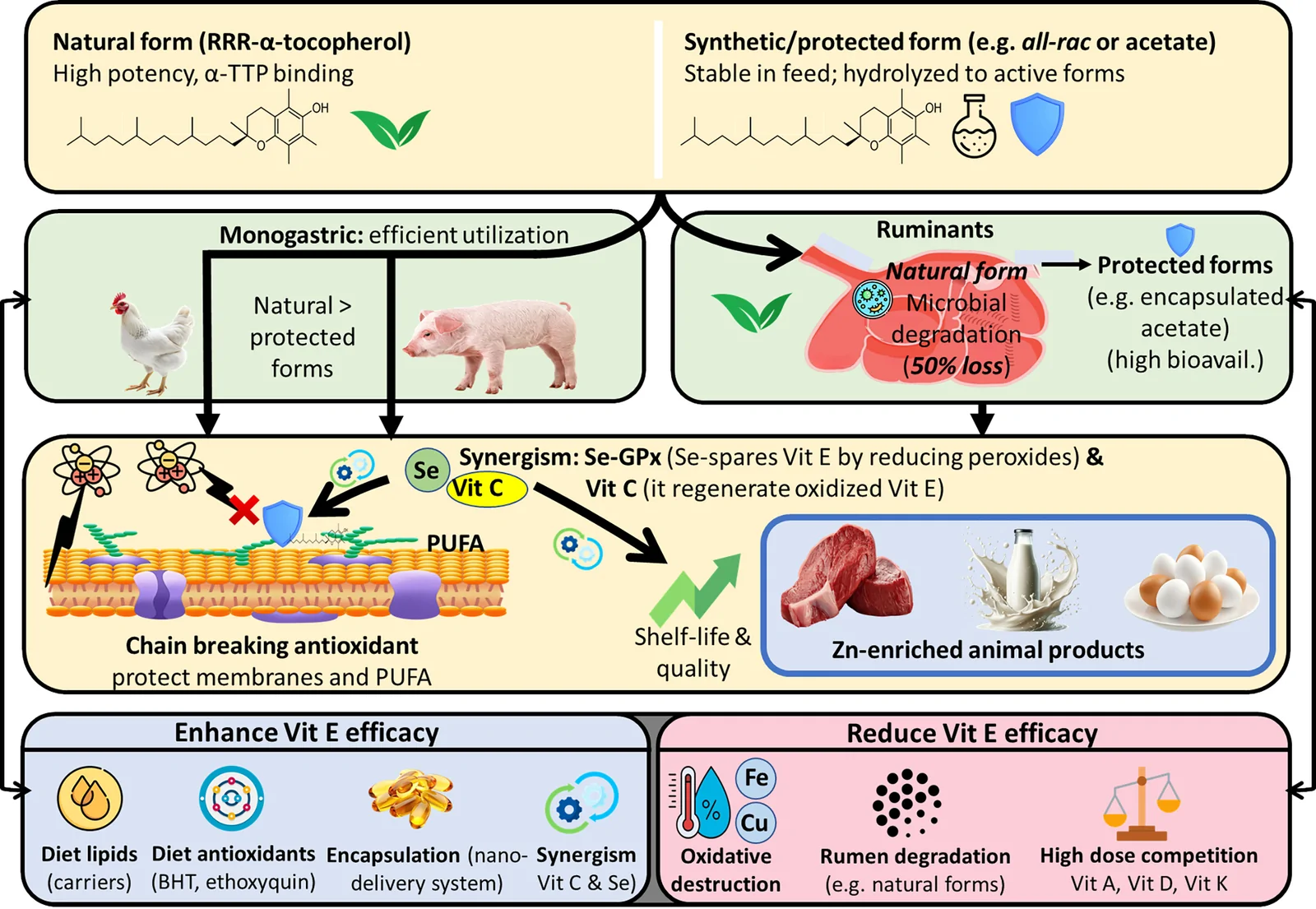
The fundamental role and interactive network of Vitamin E (Vit E) in animal nutrition and their product enrichment strategies.

Despite Vit E being shown to be beneficial for animals and their derived products, several factors, like chemical structure, interactions within the dietary matrix and digestive physiology particular to species, can influence its efficacy in animal nutrition. All of these factors will be thoroughly examined in the sections that follow (illustratively summarized in Fig. 9).

##### 3.3.3.1 Practical approaches related to efficacy

Vit E supplementation typically promotes the health benefits and quality of animal-based products; its ***efficacy*** depends on multiple factors. Natural forms like RRR-α-tocopherol and d-α-tocopherol exhibit high bioavailability and biopotency compared to synthetic and complexed Vit E forms (e.g., all-rac-α-tocopherol, dl-α-tocopherol, and α-tocopherol acetate) (Cheng et al., 2016; Dersjant-Li & Peisker, 2009; Leal et al., 2019), enabling reduced supplementation doses. However, species-specific digestion and metabolism remarkably influence Vit E uptake and measurement effects, wherein ruminants are more challenging species than monogastric animals. As an illustration, ruminal microbiota degrades unprotected Vit E, reducing its bioavailability by about 30–50% in ruminants (Alderson et al., 1971; Hidiroglou et al., 1992; Masoero et al., 1997). Hence, rumen-protected forms (e.g., encapsulated α-tocopheryl acetate) are more beneficial for ruminants due to their enhanced stability attributes (Chikunya et al., 2004; Hymøller & Jensen, 2010), resulting in high Vit E content within the small intestinal tract, where absorption takes place. High doses of Vit E are typically needed to overcome the oxidation of PUFA-enriched meat, as demonstrated in ruminants (De Almeida et al., 2015). Vit E supplementation is likely necessary for the ruminal microbiome profile, as it influences both liquid- and solid-associated bacteria as well as protozoa in a manner to enhance dry matter degradation and fermentation (Belanche et al., 2016, 2017; Hernández-Mendo et al., 2017). On the other hand, unprotected Vit E forms remain more effective in swine and poultry. These monogastric species particularly benefit from Vit E supplementation (with no ruminal degradation), which reduces their high oxidative stress susceptibility associated with rapid growth rates. Free/unprotected Vit E is highly susceptible to oxidative degradation during feed manufacturing, pelleting, and storage, which is a consequence of exposure to high heat, moisture, and pro-oxidant elements (e.g., iron and copper). In this respect, the active hydrogen-donating site, the phenolic hydroxyl group at C-6 of the chroman ring, is primarily targeted due to its reactivity with molecular oxygen and redox-active metals such as iron and copper (Masoero et al., 1997), markedly reducing Vit E bioavailability and bioactivity in animals. Among Vit E forms used in feed, free/unesterified forms such as RRR-α-tocopherol are susceptible to oxidative degradation during feed processing, pelleting, and storage due to the free phenolic hydroxyl group at C-6. On the contrary, α-tocopheryl acetate, in which this hydroxyl is esterified, is substantially more stable and is consequently the predominant commercial feed supplement form; however, it is biologically inert until intestinal hydrolysis by esterases (primarily involving intestinal brush-border lipases and pancreatic esterase) to release the biologically active α-tocopherol prior to absorption. Notwithstanding, this hydrolyzation process typically introduces an additional source of variability in Vit E bioavailability, notably in newborn animals whose pancreatic enzymes have not fully developed yet and in ruminants where the environment in the rumen may break down certain of the ester link. However, to mitigate the potential of oxidative issues, incorporating antioxidants (e.g., ethoxyquin, butylated hydroxytoluene (BHT), and butylated hydroxyanisole (BHA)) and performing encapsulation techniques (e.g., nanochannels or nanoliposomes) and/or innovative delivery systems (e.g., nanoemulsions and liposomes) can prevent the oxidative destruction of vitamins A and E, as well as carotenoids like xanthophylls (Amiri et al., 2018; Barros de Medeiros et al., 2022; Błaszczyk et al., 2013; Kobayashi et al., 2025). Considerably, as per unit concentration, synthetic antioxidants (like BHT and ethoxyquin) outperform natural antioxidants (e.g., rosemary extract (carnosic acid/carnosol), mixed tocopherols and ascorbyl palmitate); however, due to concerns of health risks linked to synthetic antioxidants, natural ones have become increasingly common in feed formulation (Mesloub & Yurdugül, 2024). Although plant extracts are receiving massive attention and gaining favour, their efficacy compared to pure Vit E must be cautiously managed; for example, despite that grape seed extract, tilapia hydrolysate, and olive leaf exhibited mild antioxidant potential in pork (Bernardi et al., 2022) and egg yolk (Botsoglou et al., 2013), traditional dietary Vit E was more effective during storage for pork and decreased yolk MDA levels during iron-induced oxidative challenge, underscoring the challenge associated with natural alternatives fully replacing Vit E in highly unsaturated feeds. Notwithstanding, providing appropriate storage conditions is likely one of the most critical aspects needed to maintain Vit E content in feed. Within this context, further research is required, especially on optimizing delivery systems for different animal species and production stages.

##### 3.3.3.2 The critical role of nutrient interactions

The ***interaction*** between dietary compounds and Vit E is critical for optimizing its efficacy. Dietary lipids/fats significantly affect Vit E bioavailability, serving as a medium/carrier for fat-soluble vitamins (A, D, E, and K) during absorption (Goncalves et al., 2015). These vitamins typically share similar transporters, like scavenger receptor class B type 1 (SR-B1) and Niemann-Pick C1-Like 1 (NPC1L1) transporters, potentially competing (antagonistic interaction) and reducing Vit E uptake when consumed together in very high pharmacological doses (Borel et al., 2013; Yamanashi et al., 2017). Accordingly, the competition for absorption transporters is minimal at standard enrichment levels. Under excessive intake, vitamins E and K, in particular, typically compete during distal intestinal absorption (Goncalves et al., 2015), and vitamin K may interfere with the activity of α-TTP binding in the liver (Eichinger, 2016). Vitamins A and E have the potential to complement or counteract one another. Vit E absorption is enhanced by Vit A, though bioavailability may be reduced at high levels. With regard to vitamins D and E, a synergistic interaction within cellular processes has been proposed, likely engaging the coregulation of nuclear receptor-mediated transcription (in which both 1,25-dihydroxyvitamin D₃ (calcitriol) and α-tocopherol have been reported to modulate gene expression through vitamin D receptor (VDR) and through Nrf2-mediated antioxidant response elements (ARE), respectively), as well as evidence of cross-talk between these pathways in bone and immune cells. However, the mechanism is not yet fully understood in livestock species (Guilland, 2011), which underscores the incomplete picture of understanding fat-soluble vitamin interaction biology relevant to feed formulation and enrichment strategy design. Phytate and soluble fibre (e.g., pectin and β-glucans) also have the potential to impair Vit E efficacy, mainly through chelating synergistic minerals (Zn, mg, and Se) (Pires et al., 2023) and reducing micelle formation via bile acid binding (Beane et al., 2021), respectively. From a biological activity point of view, Vit E acts as a protective agent, decelerating the lipid-peroxidation process in feeds rich in unsaturated FAs (like those rich in vegetable oil or fish oil), thereby preserving feed properties over time and enhancing lipid metabolism and product outcomes. However, the relationship between Vit E and dietary lipid is strictly stoichiometric, in which Vit E preserves the lipid profile (it prevents FA oxidation) but does not alter the composition (Franczyk-Żarów et al., 2019). This protective interaction was further demonstrated by de Almeida et al. (2015), who showed that the functional value of PUFA enrichment in ruminant meat is contingent upon adequate Vit E protection. Synergistic (bioavailability and mechanistic) interactions between Se and Vit E are also well-documented (Dalia et al., 2018; Hussain et al., 2024; Kotit et al., 2025; Pečjak et al., 2022; Scheideler et al., 2010). Se, an essential element of glutathione peroxidase, simultaneously reduces lipid hydroperoxides (LOOH) to harmless alcohols; thus, it prevents the propagation of lipid peroxidation chains that consume Vit E. This "sparing effect" means that adequate Se status is requisite for leveraging the efficacy of Vit E. This is powerfully demonstrated in eggs (Scheideler et al., 2010), where the combination of Vit E and Se led to superior enrichment of both nutrients compared to either alone. Furthermore, these nutrients collaborate to neutralize oxidative stress, consequently enhancing immune responses, as evidenced by enhanced neutrophil activity, lymphocyte proliferation, and cell-mediated immunity in livestock (Hossain et al., 2025). In light of these findings, Vit E deficiency can be partially offset by adequate Se intake, and vice versa. Further nutrient synergies exist between Vit E and Zn, magnesium (Ahmed & Nafea, 2024; De Grande et al., 2022; Liao et al., 2022) or Vit C (which acts as a regenerator of oxidized Vit E) (Goncalves et al., 2015; Pečjak et al., 2022), although Vit C interactions may be tissue-specific (Sato et al., 2022). Despite these synergisms, antioxidant protection is known to have a functional upper limit; for example, high doses of Vit E (e.g., 2000 IU) did not stop the deterioration of sensorial properties in milk from cows fed a highly DHA-rich microalgae diet (Bragaglio et al., 2015), pointing out that severe ruminal or post-absorptive lipid oxidation in ruminants can possibly surpass Vit E protective capacity. Vit C can chemically "recycle" the tocopheroxyl radical back to active tocopherol at the membrane-water interface, augmenting the antioxidant capacity of Vit E. Similarly, the combination of Vit E and citric acid has been reported to boost the activity of antioxidant enzymes and increase the polyunsaturation level in juvenile cobia (Xu et al., 2020), indicating a potential beneficial interaction. Although this positive interplay is not yet proven to be a direct or indirect consequence, it probably can be attributed to the role of citrate in improving the digestion environment (low pH, high solubility of certain compounds, and high digestive enzyme activities (Liu & Skibsted, 2023; Yang et al., 2015)), thereby facilitating micelle formation and absorption. These interdependencies highlight the complementary roles of these nutrients in maintaining health, preventing disease, and enhancing the quality of animal products. These complexities indicate that there must be an appropriate strategy to balance dietary formulation and optimize nutrient bioavailability and metabolic utilization. Herein, future research must focus on elucidating the optimal ratios of Vit E, Se, Vit C, and other phytonutrients in complex diets to maximize antioxidant protection and product enrichment.

##### 3.3.3.3 Additional limitations and future research priorities

Despite substantial progress in Vit E research (which focuses on optimizing inclusion levels, comprehending interactions with other nutrients, and measuring their combined impacts on gene expression and cellular signalling and communication), ***further directions*** can be suggested. As of today, enormous efforts have been made in the area of the potential replacement of dietary polyphenols with dietary Vit E antioxidant functionalities; however, available data derived from *in vivo* models do not support this proposal (at least as a complete replacer) due to their low bioavailability (DSM-firmenich, 2025). This limitation is practically evidenced by Bernardi et al. (2022), where natural antioxidants like grape seed extract and tilapia hydrolysate only exhibited mild antioxidant effects in pork compared to the robust efficacy of 200 mg Vit E/kg feed. Concerning this, Vit E is a lipid-phase chain-breaking antioxidant within membrane phospholipid bilayers, making it mechanistically distinct from major aqueous-phase radical scavenging and enzyme-modulating activities of most polyphenols. These polyphenols’ characteristics typically limit the access to the site of lipid peroxidation initiation. In addition, polyphenols have no equivalent in the hepatic α-TTP retention mechanism that concentrates RRR-α-tocopherol in tissues, which limits their tissue accumulation in comparison to Vit E. These mechanistic distinctions refer to the fact that the fundamental roles of Vit E are somewhat unique and not entirely replicable by polyphenols, which must be understood and taken into consideration during feed formulation, especially when designing PUFA-enriched functional food strategies. A further marked gap in Vit E investigations can be seen in the biochemical interaction with Vit K (Traber, 2008), especially in terms of blood clotting and bleeding. Understanding this complex bio-interaction and its impact on other metabolic events would be beneficial for the health of both livestock animals and humans, as well as for the developing of enrichment strategies and intake levels of Vit E to target consumers.

##### 3.3.3.4 Vitamin E section brief summary

The enrichment of Vit E is applicable and dose-dependent, while its efficacy is maximized when other antioxidants (e.g., Vit C and Se) are incorporated. Vit E inclusion level must scale dietary PUFA load; while it may effectively stabilize linseed-enriched pork and sunflower seed-enriched lamb meat, it may be inadequate to prevent sensory defects in dairy products from cows fed on high marine inclusion rates, as well as be able protect but not alter deposited FA profiles. The primary gap is in thoroughly understanding the antagonistic relationship between Vit E and K at high levels, as well as validating whether plant extracts can practically substitute Vit E lipid-phase chain-breaking function in PUFA-rich feeds, for which current evidence does not endorse equivalent efficacy.

### 3.4 Dietary effects of oils on the fatty acid composition of animal products

Dietary supplementation with oils plays an essential role in animal feeding and nutrition. Fat or oil supplementation physically reduces feed particle dust by keeping the feed particles intact, minimizing waste. They also serve as lubricants for mixing feed and improve the palatability of the feed. They also provide energy for the optimal functioning of farm animals (Kholif & Olafadehan, 2021). However, their application or usage in the context of enriching the functional value of animal products goes beyond the roles stated above, which additionally include manipulation of the FA composition of animal diets. Thus, the dietary inclusion of specific oils can be a manipulation strategy to lower medium-chain and total SFA concentrations while increasing the concentrations of total PUFA, overall n-3 PUFA, and n-6:n-3 (Shingfield et al., 2013). Oils from oilseeds and marine sources rich in PUFAs are mostly used to improve the n-3 contents of animal products. For this particular review, linseed oil, rapeseed oil, fish oil, and microalgae will be considered.

***Linseed oil*** (LO), also known as flaxseed oil and a plant-based α-linolenic acid (C18:3n3, or ALA) source, is one of the most commonly supplemented plant-derived oils in animal nutrition, which is attributed to the high proportion of essential ALA, an essential n-3 FA that cannot be synthesized endogenously. Its supplementation in animal diets has been established to increase the proportions of ALA and its elongated n-3 metabolites in their products. Beyond animal product enrichment, linseed oil has a positive effect on growth and reproductive system performance (Akhtar et al., 2024; Więcek et al., 2010), which is likely attributed to the elevation in triiodothyronine (T3) and insulin, suggesting a broader metabolic rate.

***Rapeseed oil*** (RO), also called canola oil, is widely used as a source of energy and ALA, although its n-3 PUFA concentration is lower than that of LO (Alves et al., 2019). Its effectiveness in enriching the concentration of long-chain FAs in animal products has been documented in multiple studies (as discussed below). Moreover, it has also been shown to reduce heat stress in animals (Elbaz et al., 2023), thereby improving feed utilization and potentially lowering mortality rates.

***Fish and microalgae oils*** (FO and MO, respectively), which are available in liquid, powdered and coated forms, are marine sources that are known to be rich in C20:5n3 (eicosapentaenoic acid, or EPA) and DHA concentrations, representing appropriate strategies for enriching animal products with very long-chain n-3 PUFAs. These oils are commonly available in different compositions, primarily determined by the species from which they were extracted, as well as the method of extraction. Among these sources, microalgae are the basis of the marine food chain and subsequently accumulate in the fish body; hence, MO is a direct and sustainable source of n-3 PUFAs. This oil source typically reduces the reliance on FO and contributes to environmental preservation (Shah et al., 2018; Togarcheti & Padamati, 2021). The functional value of these oil supplements also contributes to various aspects of animal performance, including improved growth, enhanced fertility, bolstered immunity, and increased bone strength in livestock (Lee-Okada et al., 2025). In addition, from a human perspective, the consumption of animal n-3 FA-enriched animal products has been associated in observational and preclinical studies with improvements in blood lipid profile (decreased plasma triglycerides), cardiovascular markers and inflammatory indices (Kralik et al., 2020; Kralik et al., 2023; Usturoi et al., 2025); however, these associations are derived primarily from studies using isolated n-3 FA supplements or fish consumption rather than enriched animal products specifically, and robust controlled human intervention trials using these functional animal products are needed to confirm such effects. Due to these benefits, FO and MO have received considerable attention in the areas of animal nutrition and functional food production within the past 15 years.

#### 3.4.1 Core biological concepts of fatty acid enrichments

To achieve a successful fortification approach, mainly through FAs of oils, it is valuable to consider the biological fundamentals related to intestinal absorption, bioconversion, and incorporation into tissues (Bionaz et al., 2020; Oketch et al., 2023). In addition, the ruminal bioprocessing environment, the metabolic fate of ALA, and oxidative stability are likely the most crucial factors, reasoned by their remarkable contribution and the prediction and efficacy of the strategy.

##### 3.4.1.1 Bioavailability of fatty acids

The oil type and digestive system type play a crucial role in determining the bioavailability of n-3 PUFAs. For example, marine sources are considered to display a higher bioavailability rate compared to plant-based oil sources, as the latter necessitate a greater biotransformation process to obtain highly bioactive FAs (Lane et al., 2022), which is generally known to be limited in terrestrial animals. Comparing marine sources, reports on krill oil, a 30–65% phospholipid-based oil extracted from shrimp-like marine animals, revealed a higher n-3 bioavailability index compared with those that are triglyceride-based FOs (Ahn et al., 2018; Ramprasath et al., 2013; Schuchardt et al., 2011). However, this efficiency is likely to decrease over time, as Vosskötter et al. (2023) reported a similar bioavailability between krill oil and FO in long-term studies. Overall, discrepancies across marine sources likely emerge from the chemical nature of the oil type, in which, unlike triglycerides, phospholipids are partially absorbed in water (the phosphorus group), suggesting a different absorption mechanism that may increase bioavailability. In addition, oils with high free FAs (FFAs) and re-esterified triglyceride levels have higher bioavailability compared to those with a large ethyl ester (EE) proportion (Alijani et al., 2025; Dyerberg et al., 2010; Mu & Müllertz, 2015; Vosskötter et al., 2023). Hence, the type of oil may explain the potential variability in outcomes under the same inclusion levels of oils that are relatively similar in FA composition. With regard to the digestive system type, a detailed discussion has been carried out in the biohydrogenation section.

##### 3.4.1.2 Ruminal biohydrogenation of dietary fatty acids

In contrast to monogastric animals, the ruminal biohydrogenation process is a factual challenge for the enrichment protocol in ruminants, wherewith rumen microbes convert dietary UFAs like LA and ALA into saturated FAs (e.g., C18:0 (stearic acid)) (Yakubu et al., 2023). Typically, the reduction of double bonds in UFAs generates various intermediates (e.g., conjugated linoleic acids (CLAs) and *trans*-FAs) prior to the ultimate formation of saturated FA forms. Physiologically, these intermediates are known to exhibit potential importance: CLA isomers are associated with health benefits, while the trans-FAs correlate with health implications (Hoffmann et al., 2015; Honkanen et al., 2012; Song & Kennelly, 2003). However, the hydrogenation process drastically reduces the flow of essential FAs and bioactive PUFAs to the intestine for absorption, as well as maximizes the loss in *cis*-FAs by forming more *trans*-FAs. Thus, consequences of biohydrogenation include health implications and poor nutritional values of animal-based products. A further indirect implication of hydrogeneration is related to the environment, in which generated hydrogen can be employed in the production of methane (CH_4_), a potent greenhouse gas, and its bioproduction compromises the up-taken/preserved energy of animals. Generally, several nutritional strategies have shown effectiveness in mitigating/reducing the ruminal hydrogenation process. Among these strategies, the use of rumen-protected or encapsulated oils, calcium-salified lipid technologies, or whole oilseeds where the seed coat provides partial protection (as seen with crushed rapeseed (Hoffmann et al., 2016)) is of great recognition, especially in relation to enrichment efficacy in ruminants. Within the context of environmental issues, the manipulation of the ruminal microbial profile via probiotics and microbial interventions has been demonstrated to effectively decrease CH_4_ production and emission (Lan & Yang, 2019; Tanaka et al., 2024).

##### 3.4.1.3 The ALA conversion pathway and its limitations

The main intent of animal product fortification is to improve the content of essential n-3 FAs, such as ALA, EPA and DHA, as well as to decrease the n-6:n-3 FAs ratio in animal products (see Table 3). Through the process of lipid metabolism, these bioactive FAs are transferred (in the same structure or upon conversion into other PUFA structures (e.g., ALA → EPA and DHA), catalyzed by elongase and desaturase enzymes) into the tissues of animals, thereby improving the functional value of animal products, such as meat, eggs, and milk. However, the efficiency of ALA bioconversion to EPA and DHA is limited in most terrestrial species by multiple enzymatic steps: the rate-limiting Δ6-desaturase (D6D, encoded by *FADS2*) acts twice in DHA synthesis, while ELOVL5 elongase, Δ5-desaturase (*FADS1*), ELOVL2, and peroxisomal β-oxidation are also required for the complete ALA → EPA → DPA → DHA pathway (Sprecher, 2000). Within this context, D6D is needed twice in the full DHA synthesis pathway. Moreover, the n-3 and n-6 PUFA elongation/desaturation pathways share D6D and D5D as common enzymes; high dietary n-6 FA (C18:2n6, which is known as linoleic acid or LA) exercises competitive substrate pressure, suppressing further n-3 FA bioconversion in typical grain-based livestock diets. With great emphasis, poultry exhibit low bioconversion efficacy of ALA (e.g., from RO or LO) to EPA/DHA (Carragher et al., 2016), in which the combination of these multi-mechanistic steps (notwithstanding the low D6D activity linked to *FADS2* gene polymorphisms (Zhu et al., 2014a), the gene encodes D6D activity) severely restricts DHA yield from ALA precursors, necessitating direct DHA supplementation from marine sources like FO or MO (Glencross et al., 2025). However, this event may vary across poultry breeds, as expressions of FADS1, ELOVL5, FATP1 and very low-density lipoprotein receptor (VLDLR) have been reported to be higher in Dwarf layer compared to White leghorn (Jiang et al., 2024), allowing for more ALA biotransformation into DHA.

**Table 3:** Proportion ranges of selected fatty acids in oils mentioned in the review.

**Fatty acid**	**Linseed oil**	**Rapeseed oil**	**Fish oil**	**Microalgae oil**
C16:0 (PA)	3.80 – 6.80%	2.18 – 7.91%	6.50 – 24.6%	8.40 – 41.6%
C18:0 (SA)	2.20 – 5.90%	1.15 – 14.9%	ND – 7.50%	1.00 – 3.70%
C18:1n9 (OA)	15.6 – 35.7%	56.3 – 67.7%	2.30 – 47.0%	ND – 29.2%
C18:2n6 (LA)	9.70 – 18.7%	10.5 – 20.9%	ND – 15.0%	0.60 – 14.1%
C18:3n3 (ALA)	42.4 – 61.3%	7.12 – 11.3%	ND – 6.00%	0.40 – 20.0%
C20:5n3 (EPA)	ND	ND	2.00 – 28.0%	0.70 – 35.5%
C22:5n3 (DPA)	ND	ND	ND – 4.00%	ND – 1.20%
C22:6n3 (DHA)	ND	ND	3.00 – 42.5%	ND – 8.10%
EPA+DHA	ND	ND	5.00 – 52.5%	3.00 – 35.5%

Abbreviations: ALA, α-linolenic acid; DHA, docosahexaenoic acid; EPA, eicosapentaenoic acid; LA, linoleic acid; ND, not detected/determined; OA, oleic acid; PA, palmitic acid; SA, stearic acid. The data were adapted from Ahmed & Nafea (2024), FAO-Codex Alimentarius Commission (2024), Harwood (2019), Mikołajczak (2018), and Silska & Walkowiak (2019).

#### 3.4.2 Dietary oils and fatty acid composition of animal products

Generally, the literature confirms the applicability of lipid enrichment strategies, with variation in the metabolic efficacy, as dietary ALA (abundant in plant sources) conversion to EPA/DHA in tissues is limited, emphasizing the need for marine oil sources for their biofortification. Moreover, it also highlights species-specific efficacy, wherein pig and poultry efficiently deposited PUFAs, while ruminants typically benefit more from protected oils. Studies on the effects of LO, FO and MO on the composition and quality of animal products are abundant in the literature. On the other hand, compared to LO, fewer studies have focused on RO in the past one and a half decades, probably because of its relatively lower content of ALA. It is important to understand that the following subtopics within this section only discuss key findings across various livestock species.

##### 3.4.2.1 Enrichment of meat products

In monogastric animals, specifically ***poultry and swine***, the choice of oil dictates the final n-3 FA profile in meat, as plant-based oils (like LO and RO) are effective for increasing ALA (linear relationship), total PUFA (typically increasing the PUFA:SFA ratio) and, to a lesser extent, EPA, C22:5n3 (docosapentaenoic acid, or DPA) and DHA, consequently reducing the n-6:n-3 ratio. This has been demonstrated in broiler thigh and breast muscles (with 33-100% of LO replacing soybean oil (SO) at 2% and 3% in the diet (P ≤ 0.05); 2–5% LO inclusion level in the diet (P ≤ 0.05); 3% RO at feed; or 25–100% of RO replacing palm oil (PO) at 1.5%, 3%, and 4.5% in the feed (P ≤ 0.001)) (Ali et al., 2023; Chinnasamy et al., 2022; Kralik et al., 2025; Mech et al., 2021; Marzec et al., 2020; Milanković et al., 2019; Panda et al., 2015; Szymczyk & Szczurek, 2016; Starčević et al. 2014), meat of local chicken breeds (with 2.5–4% Lo at feed) (Farahiyaha et al., 2025), Chinese crested white ducks’ muscles (with 2% of dietary LO inclusion level fed from 28 to 56 days of age; or 10% RO inclusion rate) (Shahid et al., 2019; Zhang et al., 2023), turkey meat (Jankowski et al., 2012), ostrich meat (with 4% and 8% of LO at feed, or 5% and 10% of RO inclusion level) (Polawska et al., 2013), and pig muscles (with 25 or 50 g LO/kg feed (P ≤ 0.05); 1–5% LO in diet; 2–4% RO in feed (P ≤ 0.05); and 0.15% SO+ 2.85% LO in diet (providing n-6:n-3 ratio of 3:1)) (Bernardi et al., 2022; Huang et al., 2020; Kralik et al., 2010; Leikus et al., 2018; Nong et al., 2020; Okrouhlá et al., 2018; Vehovský et al., 2019). These studies also highlighted that LO induces ALA deposition more than RO, whereas dietary RO drastically increased the C18:1n9 proportion. Furthermore, the duration of dietary oil supplementation is likely a determinant factor in the enrichment strategy outcomes. Moreover, 20 days of 10% RO supplementation was described as an optimal period for ducks, as supplementation for 30 days did not reveal marginal additional enrichment, providing an effective species-specific recommendation for waterfowl enrichment programs. Except for the finding of Bernardi et al. (2022), these lipid modifications were not associated with trade-off sensorial properties, at least at dietary inclusion levels below 4% for poultry and 5% for pigs (Ali et al., 2023; Huang et al., 2020; Panda et al., 2015; Vehovský et al., 2019), nor did they increase the triglycerides and cholesterol concentration in subcutaneous fat (Nong et al., 2020), marking potential positive health attributes. Herein, dietary antioxidants (e.g., Vit E) play a beneficial role in preventing sensorial degradation through boosting oxidative stability, as demonstrated with 0.2 g Vit E/kg feed of pig, outperforming natural plant extracts like grape pomace (Bernardi et al., 2022). Furthermore, 0.3 mg SeMet/kg diet boosted oxidative stability in pig muscles enriched by 3% dietary LO and FO, wherein the proportions of ALA and total n-3 FAs were significantly high (Yin et al., 2025).

However, as highlighted earlier, the reduced conversion efficiency of ALA to DHA in monogastric species, especially birds, is attributed to their limited activity of D6D; achieving substantial DHA enrichment generally requires direct DHA supplementation, as endogenous conversion contributes only modestly to tissue DHA pools in most terrestrial species. For instance, 1% or 2% of MO supplementation substantially elevated EPA and DHA contents in broiler breast and thigh (P ≤ 0.05), as well as lowered n-6:n-3 ratio without the sensory defects associated with FO (Long et al., 2018). Konieczka et al. (2017a) further detailed that broiler supplementation with the combination of 1% FO and 6% flaxseed or 5.8% rapeseed for just two weeks prior to slaughter was sufficient to label the meat as "high in n-3", providing 33% of the recommended daily EPA/DHA intake per 100 g of breast meat. Though FO has been shown to be effective for depositing EPA and DHA directly (e.g., Ibrahim et al. (2018), Muhammed et al. (2015), Aghaei et al. (2012), Maroufyan et al. (2011), Narciso-Gaytán et al. (2011), Poureslami et al. (2010) and Saleh et al. (2010) used 1-6% dietary FO or FO replacing SO at 25% and 50% to increase EPA and DHA in broiler muscles and liver), high dietary inclusion levels (> 2 or 3.5%) can impair broiler growth rate and deteriorate carcass qualities (Aghaei et al., 2012; Royan et al., 2013)). Moreover, n-3 PUFA enrichment through dietary FO comes at the cost of lower sensory scores due to fishy flavours, highlighting a key trade-off. For example, the earlier study of Saleh et al. (2010) showed the dietary FO-induced elevations in broiler muscle EPA and DHA were concurrent with high TBARS concentrations during storage. Additionally, in a swine model, when gilts were fed diets supplemented with different oils (1% PO, 1% SO, 1% LO, 1% FO, and 1% Echium oil), both EPA and DHA contents increased with FO supplementation, but the aroma and flavour (fishy properties) of the meat from this group differed, in which sensorial qualities were slightly lower (P = 0.022) (van Wyngaard et al., 2023). However, the meat cooking properties remained unchanged regardless of the treatments, which is plausibly attainable under low FO supplementation (1%). These findings raise concerns and highlight a key practical limitation of using FO in monogastric diets, making MO the sensorially superior choice for monogastric meat enrichment. Combinations of CLA and FO have also been explored, demonstrating that CLA alone increased less desirable SFA in broiler meat, and with FO or LO co-supplementation, it restored the favourable n-6:n-3 ratio (Shin et al., 2011a). This study also found that the highest DHA content was associated with FO supplementation (P ≤ 0.05), while, in a relatively similar study by Shin et al. (2011b), the greatest ALA deposition was achieved through LO feeding (P ≤ 0.05). These findings confirm the necessity of including marine oils in monogastric diets. This superior DHA deposition may not be limited to rich dietary DHA levels; it may emerge as a consequence of downregulation of hepatic gene expression that regulates their oxidation (Tao et al., 2018). This likely reflects DHA-mediated activation of PPAR nuclear receptors and suppression of hepatic β-oxidation gene networks (including ACOX1 acyl-CoA oxidase 1 (ACOX1) and the α-subunit of mitochondrial trifunctional protein (HADHA)), creating a DHA-sparing effect that is favouring DHA’s esterification into tissues over catabolism. In addition, DHA-enriched membrane phospholipids can substantially influence the fluidity and trafficking of FA-binding proteins (FABP), directing long-chain PUFAs toward esterification pathways. These mechanisms typically improve DHA retention in tissues and its transfer to muscle, justifying superior accumulation with MO vs. LO; however, the direct evidence in broiler liver remains limited. Notably, within the literature, a downstream consumer model (using male Wistar rats) demonstrated the benefits of consuming the n-3 PUFAs, Vit E and Se-enriched chicken meat. According to Konieczka et al. (2017b), 8 weeks of feeding on enriched meat markedly increased DHA content in the rat brain and liver, especially with dietary Vit E supplementation, thereby providing preliminary quantifiable supporting data on the passing benefit to consumers from animal products produced on the basis of dietary enrichment strategies.

For ***ruminant*** meat, the challenge of biohydrogenation must be overcome; however, unprotected oil can still provide partial enrichment. For example, plant oils like LO (100 and 200 g/day at feed DM, or 6% LO at feed) and RO (3% inclusion rate) in beef or lamb diets substantially improved n-3 FAs, PUFA and n-3:n-6 ratio (P ≤ 0.05 – 0.001) without compromising performance, water holding capacity or carcass qualities (Quiñones et al., 2019; Suksombat et al., 2016a; Noci et al., 2011). Moreover, LO appeared to provide dual benefits by increasing n-3 FA levels and reducing CH_4_ emission by 19% in bulls, as shown at 12.8% supplementation per DM diet by Roskam et al. (2025). In addition, Bayat et al. (2018) demonstrated CH_4_ reduction with a 5% inclusion rate, using LO or RO in the diet of Nordic Red cows. However, this high LO or RO incorporation, in a prolonged feeding term, most possibly compromises performance and meat quality attributes; for instance, in a beef model, 8% of dietary LO reduced meat colour stability (Morittu et al., 2021). With respect to this event, the concurrent supplementation with 2500 IU of Vit E protects subcutaneous lipids from oxidation and preserves meat colour. However, the choice of dietary antioxidant type may influence efficacy, as demonstrated via inorganic Se co-supplementation that upregulated Δ9-desaturation and selectively boosted beneficial *cis*-9, *trans*-11 CLA isomers (Czauderna et al., 2012).

Remaining with ruminants, studies on protected forms of FO and MO have also shown to positively contribute to products’ nutritional values, as these sources bypass the rumen and provide long-chain PUFAs for direct absorption in the small intestine. However, this does not neglect the partial efficiency of unprotected marine oils in bypassing hydrogenation to increase CLA and n-3 levels. As an illustration, regarding beef, Byrne et al. (2021), Corino et al. (2022) and Zakariapour Bahnamiri et al. (2019) successfully used FO (2.1% supplementation) and protected LO (140 g/animal/day) supplements to lower the n-6:n-3 ratio in brisket and *longissimus lumborum* muscles without affecting carcass traits (P ≤ 0.05, P ≤ 0.001, and P ≤ 0.01, respectively), though Corino et al. (2022) noted a slight increase in malondialdehyde that remained within acceptable thresholds. Relatively similar trends to Byrne et al. (2021) and Zakariapour Bahnamiri et al. (2019) have been reported in lambs (fed 1-3% FO-enriched feed (Annett et al., 2011; De Marzo et al., 2023; Jaworska et al., 2016; Parvar et al., 2017)) and goats (offered 2.5% FO at feed (Sondakh et al., 2025)), depicting the highest EPA and DHA concentrations (P ≤ 0.01 and P ≤ 0.05, respectively) in muscles/carcass, without compromising animal performance or meat characteristics. Díaz et al. (2017) also observed similar findings (3.1% and 4.48% further increments for DHA and total n-3 levels, respectively) in the intramuscular fat of lambs supplemented with 2% dietary MO rich in DHA on the FA composition. Nevertheless, the study of de la Fuente-Vázquez (2014) highlighted a critical trade-off in lambs related to growth performance and lipid oxidation when compared to meat from lambs fed on extruded linseed. Hence, in order to avoid trade-off implications, it is highly recommended to avoid high dietary inclusion levels of FO, to use MO as an alternative, and/or to implement a multi-additives strategy comprising an adequate level of antioxidants.

Meat enrichment through oils has been investigated in ***other species***, aiming mainly to boost health-promoting attributes. For example, 1.5% Optomega-50 FO (Optivite International Ltd., Barcelona, Spain), 3% and 9% LO inclusion rates, or 2.5-3% of LO+ 0.3-0.46 mg organic Se/kg feed significantly improved FA profiles of rabbit muscles and perirenal fat (increased n-3 PUFAs while decreasing SFA and n-6:n-3 (P ≤ 0.005 - 0.001) (ca. 2.20 *vs*. 7.29), alongside enhanced growth performance (Rodríguez et al., 2019; Matics et al., 2017; Saleh, 2013; Trebušak et al., 2011). These studies emerged with further findings and conclusions, like LO effective enrichment is influenced by the duration (more than 4 weeks did not provide marginal enrichment; however, further investigation under commercial conditions is needed), and the oxidation is the key constraint for the enrichment strategy (especially LO-based approaches), requiring co-supplementation with antioxidants. However, the oxidative vulnerability of this enrichment is species-pervasive, as Danuta et al. (2020) provided critical evidence that while 4% of dietary FO increased PUFA content and longissimus muscle mass in rabbits, it resulted in the highest lipid oxidation and adverse rancid sensory notes; crucially, Se-yeast alone was insufficient to counteract this pro-oxidant effect, highlighting the potential benefit achieved from Vit E in high-PUFA marine diets. Similarly, Dal Bosco et al. (2018) found that 3% of dietary LO successfully modified the PUFA profile of rabbit loins but also significantly increased TBARS values during retail display, necessitating optimized packaging (e.g., modified atmosphere or vacuum) to preserve lipid stability.

From the above, it can be concluded that n-3 PUFA enrichment strategies are dichotomous, wherein plant-based oils are cost effective tools to increase ALA, EPA and DPA, while marine oils are advisable for maximizing DHA proportion; however, the FO sensory trade-off in monogastrics restricts its use, making MO an optimal alternative additive. In ruminants, protected oil forms are beneficial to avoid the hydrogenation process. Moreover, high inclusion rates of dietary oils can compromise oxidative stability (> 8% LO) and reduce CH_4_ emission (> 12.5% LO). Therefore, lipid protection is non-negotiable for PUFA enrichment, shifting the formulation focus from simply "which oil" to "which delivery matrix". Herein, excessive PUFA enrichment without antioxidant protection consistently fails, underscoring the importance of antioxidants (with organic Se alone being insufficient to prevent marine oil-induced oxidation) and package interventions.

##### 3.4.2.2 Enrichment of eggs

Hens do not only deposit dietary FAs in their muscles, but they also incorporate them into the egg yolk. Among poultry species, the majority of the literature focused on chicken (*Gallus domesticus*), as its product is known to be the most consumed type of eggs globally. In this regard, supplemental LO has been used for the production of n-3-rich eggs for the functional food market (Ahmad et al., 2017). Among the literature, plant-based oils, especially LO, have been highly investigated, and both LO and RO at a 3-5% dietary inclusion levels demonstrated efficacy in ALA enrichment (4- to 5-fold more) in layer egg yolk (P ≤ 0.05 - P ≤ 0.0001), which further reduced the n-6:n-3 ratio in a dose-dependent manner (Altaçli et al., 2022; Ceylan et al., 2011; Hudečková et al., 2012; Omidi et al., 2015; Oliveira et al., 2010; Petrović et al., 2012), while this ratio stabilized after 5 weeks without affecting yolk fat and cholesterol level (Petrović et al. 2012, used 1–4% LO at feed). These patterns are likely evident across poultry species, as 3% LO in a quail diet positively improved the egg n-3 level and its ratio to n-6 level, from 0.05 (control) to 0.18 (Citil et al., 2011). In layer hens, Herkeľ et al. (2016) and Batkowska et al. (2021) confirmed that 2.5–3% dietary LO safely enriches eggs with n-3 ALA and total PUFAs (P ≤ 0.01) and without negatively impacting cholesterol levels or general egg quality. In relatively similar LO inclusion levels (2%, 3% and 4%), elevation of n-3 FAs has been observed in a dose-dependent manner during hot summer, with a 4% inclusion rate compromising feed intake under heat stress (Ahmad et al., 2022), pointing out the interaction between environmental stressors and oil supplementation levels. However, the physical form of dietary lipid is of paramount importance, in which extracted LO is twice as efficient at depositing ALA into the yolk compared to milled flaxseed at the same dietary inclusion levels, from 0.5 to 5% (Ehr et al., 2017). From an economical stand point, in a recent study published after 2025, Hazarika et al. (2026) identified 2.5% LO as the optimal threshold for maximizing n-3 enrichment while managing production costs. With respect to LO, alternative plant-based sources, such as hemp seed oil and chia seed flour, have been reported to elevate n-3 FAs in egg yolk (Coorey et al., 2015; Kanbur et al., 2022).

Notably, when high levels (> 3%) of LO were used in the layer diet, the levels of ALA and DHA in egg yolk were significantly greater (P ≤ 0.05) (Duan et al., 2021); however, this finding warrants cautious interpretation given the well-established low (≤ 1%) ALA-to-DHA bioconversion efficiency in poultry, mimicking the observed pattern of meat (Section 3.4.2.1). Therefore, this low bioconversion is a major constraint, wherein LO depicted 6% enrichment efficacy for n-3 very long-chain FAs in yolk, compared to 33-55% efficacy for marine oils (Lemahieu et al., 2015). Furthermore, Neijat et al. (2016) showed that hens fed preformed DHA from algal sources deposited 3-fold more DHA into the yolk than those fed ALA from flaxseed oil, with maximal n-3 deposition attained by week 2 of supplementation. In a direct comparison, Li et al., 2023b systematically demonstrated this benefit, in which DHA deposition from dietary marine oil occurred more efficiently than ALA deposition from linseed, with DHA further boosting the immune response, creating a nutritional preference for marine oil when nutritive value and immunomodulation are targeted. Zhao et al. (2021b) further demonstrated that DHA-enriched phospholipids were more efficiently deposited into egg yolk than DHA in triglyceride form; thereby, careful consideration of the lipid carrier structure is need for the optimization of enrichment strategy. It is recommended to not employ high inclusion levels LO/marine oil, aiming at avoiding implications related to oxidative stability during storage (quadratic relationship with time (Lee et al., 2021)). A relatively similar negative impact has also been observed under a high dietary RO inclusion rate (> 6%), which compromised feed intake, egg production, and oxidative stability (Gao et al., 2021). Collectively, these findings emphasize the importance of dietary antioxidants, reinforcing their need in n-3 enrichment strategies. For instance, Zou et al. (2025) showed that the supplementation of dietary Se boosted the oxidative stability of DHA-enriched eggs.

The dietary n-6:n-3 ratio plays a crucial role in the biofortification strategy and overall production. According to Attia et al. (2022a), a 9.3:1 ratio optimizes laying performance and FCR, while a much lower 5.5:1 ratio is needed to maximize n-3 deposition and boost immune responses. In addition, the incorporation of CLA to enhance functional value results in a trade-off with the lipid profile, as 0.5-1% CLA in layer feed elevated the yolk total saturation while decreasing the overall monounsaturation level, a negative effect that vegetable oils could not fully offset, highlighting that Vit E protects but does not alter this trend of lipid profile shift (Franczyk-Żarów et al., 2019). Regarding oil combination, when LO+ FO mixtures were compared to RO+ FO mixtures (Kralik et al., 2020), the mixtures containing LO were more efficient in enriching n-3 FAs and decreasing the n-6:n-3 ratio of eggs. To assess the comprehensive health benefits of these enriched eggs, Kralik et al. (2024) calculated lipid quality indices, confirming that specific oil and microalgae mixtures significantly improve the atherogenicity and thrombogenic indices of the yolk (P ≤ 0.05).

For remarkable EPA and DHA-enriched eggs, both FO and MO are highly effective. For example, compared with LO and RO, dietary FO (1–5% at feed) or FO calcium salts (0.75–2.25% at feed) inclusion or significantly (P ≤ 0.01) increased the EPA and DHA content in egg yolk (Aro et al., 2011; Ceylan et al., 2011; Ghaderi-Chaparabad et al., 2025; Valavan et al., 2013; Yalçın & Înal, 2010). Similarly, Radanović et al. (2023) and Saleh (2013) reported that replacing dietary vegetable oil or soybean oil with FO at 25–75% substantially increased yolk EPA, DHA and ALA while decreasing yolk cholesterol, though 5% FO negatively impacted feed intake and egg weight (P ≤ 0.05). Sensory challenges appear to be the primary obstacle for FO strategies. In contrast to control or LO-fed hens, eggs from FO-fed hens scored lower in general acceptance and flavour, despite the fact that dietary FO enhanced eggshell quality (Attia et al., 2022b). These elevations (P ≤ 0.01) in yolk EPA (from 10.27 to 20.10 mg/100 g egg), DHA (from 105.44 to 236.87 mg/100 g egg) and n-3 FA overall (from 204.59 to 327.35 mg/100 g egg) are dose-dependent to FO inclusion levels (0.6–1.5%), as reported by Kralik et al. (2021). Yet, the sensory trade-off continues to be the determining factor. In a relatively contrasting manner, Coorey et al. (2015) noted that EPA and DHA were only detected in eggs from FO-fed hens, while chia produced the highest overall n-3 without sensory defects. In light of these limitations, MO is highly recommended for DHA enrichment in eggs without compromising sensory attributes, thus maintaining consumer acceptance. According to Feng et al. (2020), the efficiency of incorporating DHA in egg yolk was not significant between hens fed FO and MO; however, the eggs from the FO source substantially scored lower sensorial qualities (P ≤ 0.05), notably in terms of their fishy flavour and aroma. This fishy property revealed a quadratic dose response to the DHA level, confirming that the sensory advantage is a key difference between FO- and MO-based strategies. The combination of algae (> 7.5%) and LO (3%) in layer diet has been shown to be effective in enriching ALA to 499 mg/egg and EPA+ DHA to 138 mg/egg (P ≤ 0.05) (Kim et al., 2016a), which, based on calculated FA content, would theoretically provide 31-55% of commonly cited daily n-3 FA intake recommendations per egg. Similarly, 3% or 5% of defatted microalgae increased these FAs (P ≤ 0.05) (Kim et al., 2016b); however, the enrichment rate was moderate compared to the LO group. Moran et al. (2020) further demonstrated that DHA deposition in both layer hen tissues and eggs was consistently elevated following DHA-enriched microalgae fortification over a 12-week period. This DGA accumulation was dose-dependent and reached a plateau at higher inclusion levels. However, the multi-nutrient approach is a highly promising strategy, including the combination of PUFA-rich oils and antioxidants. According to Kralik et al. (2023), the replacement of SO with a FO/RO/LO blend, alongside increased organic Se (0.47 mg/kg), Vit E (125.2 mg/kg), and lutein, resulted in eggs that are greatly enriched, where Vit E content increased 2.74-fold and lutein increased 8.94-fold, alongside elevated n-3 PUFAs. These findings corroborate those published earlier by Kralik et al. (2020), in which the dietary FO and LO mixture was more efficient in depositing n-3 FAs in yolk, which significantly increased ALA, DHA and n-3:n-6 ratio, compared to eggs from hens that received no oil or diet containing both FO and RO. Protected marine forms like FO-calcium salts offer dual benefits, as demonstrated by Ghaderi-Chaparabad et al. (2025), who reported the elevation of n-3 FAs occurred alongside the decrease in lipid peroxidation. These outcomes ultimately advocate for multi-supplementation, wherewith the biological conversion efficacy is not constrained or challenged.

***Briefly***, the weight of evidence indicates that LO, RO, FO and MO increase the beneficial n-3 PUFAs and decrease the n-6:n-3 ratio, as well as improve the nutritional quality of these products. At the commercial level, for DHA-enriched egg production, direct marine oil supplementation is optimal, with MO having the advantage of not deteriorating sensorial qualities. LO or RO at a high inclusion rate is risky, likely reducing feed intake and performance, while increasing lipid oxidation. This underscores that "more is not always better" when it comes to harmonizing enrichment goals with hen productivity and product stability. Ultimately, the precision enrichment of eggs requires a multi-nutrient formulation that considers the specific bioconversion limits of the hen, the lipid quality indices of the final product, and the scale balance need for antioxidants like Vit E and Se to stabilize the highly unsaturated matrix.

##### 3.4.2.3 Enrichment of milk

The FA profile of milk is highly responsive to dietary oils, but rumen protection is often necessary. Herein, the literature depicts successful fortification of milk obtained from different ruminant species. Gheno et al. (2024), Razzaghi et al. (2022), Hoffmann et al. (2016), Suksombat et al. (2016b), Dai et al. (2011), and Côrtes et al. (2010) reported that dietary LO (250-500 g/animal/day, or 1.9 LO calcium salts at feed), RO (2.-5% per DM feed) or crushed full-fat rapeseed (4.9% per DM diet) effectively altered bovine milk FA profiles by decreasing short- and medium-chain SFAs (with carbon chain ranges from 10 to 17, except for C13:0) while increasing ALA, long-chain UFAs and CLA isomers (P ≤ 0.001 and P ≤ 0.05). Moreover, These LO-induced elevations did not negatively compromise the milk production. When compare LO to other vegetable oils (specifically regular and high oleic sunflower oils: up to 66 g/day), LO showed higher potency in improving goat milk nutritional value, substantially decreased n-6:n-3 ratio (about 70% reduction) and linearly increased CLA (approximately 298%) (Martínez Marín et al., 2012). Similar CLA patterns have been reported in ewe milk upon the intake of 3% dietary LO alongside Vit E (Gallardo et al., 2015). The mechanism through which LO induced the milk CLA content has been elucidated by Toral et al. (2022), who used ^13^C-vaccenic acid tracer in dairy sheep and goats fed LO (2% DM). These authors reported that stearoyl-CoA desaturase is not the main limiting factor for milk CLA, whereas the dietary supply of *trans*-11 C18:1 precursor through rumen biohydrogenation is a more pivotal determinant, with comparative variations in ∆9-desaturation across sheep and goats to be considered in species-specific strategies. The physical form of dietary lipid additive is also critical, as comparatively, a lower degree of saturation in milk fat was observed in cows fed crushed full-fat rapeseed than in those fed RO, possibly due to the physical protection of the seed coat in crushed rapeseed, which reduces its exposure to ruminal hydrogenation. Regardless of LO or RO, inclusion levels must not be extreme (not practically fit with standard formulations), although improvements in the milk FA profile are attainable, as demonstrated in goats fed a diet containing 20% LO (Musco et al., 2022). Moreover, unprotected oils rich in PUFAs typically carry a potential severe risk of milk fat depression (MFD). According to Altenhofer et al. (2014), and He and Armentano (2011), a 5% per DM inclusion rate of unprotected vegetable oils rich in C18:1 (e.g., RO), LA (e.g., SO, corn oil and safflower oil) or ALA (e.g., LO) markedly decreased milk fat concentration and yield compared to a control or palm oil diet, pointing out that the dietary unsaturation degree dictates the severity of MFD. Furthermore, these studies also concluded that C18:1n9 and ALA share a similar potency for MFD. Notably, advancement technologies tremendously contributed to the tackling of potential MFD; for example, bypass LO in dairy ewes (10.8 g/day) elevated milk UFA, PUFA, and n-3 FAs (P ≤ 0.05) without negatively impacting yield (Contreras-Solís et al., 2023). These findings have been further confirmed for goat milk by Moya et al. (2023), who found that flaked linseed (3.88%) or FO (2.64% at DM) in goat diets increased the n-3:n-6 ratio and CLA concentration (P ≤ 0.05) without altering the sensory profile of milk or fresh cheese (P > 0.05).

For strategies targeting very long-chain PUFAs, unprotected FO (e.g., 2% in dairy cow feed) provides modest elevations in *cis*-9, *trans*-11 CLA and n-3 FAs (Kupczyński et al., 2011); however, the combination of FO with plant oils likely targets various PUFAs. For example, Bernard et al. (2015) observed that extruded linseeds alone or mixed with FO significantly lowered SFA and elevated n-3 PUFA and CLA in goat milk, with the combination giving the most comprehensive FA profile improvement. In addition, for goat milk, Thanh et al. (2023) demonstrated that an LO+ FO mix at 4.16% synergistically elevated ALA, EPA, DHA, and CLA (4.53 and 2.94 times higher than that of the CTR and LO alone groups, respectively) far beyond LO alone (P ≤ 0.05). Likewise, Tsiplakou and Zervas (2013a, 2013b) confirmed that a moderate combination of SO and FO increased beneficial C18:1 *trans-*11, CLA, EPA, and DHA while decreasing the SFA/UFA ratio and the atherogenicity index in both goat and ewe milk, without negative effects on milk yield or composition. Moreover, Cieślak et al. (2015) demonstrated that the FO+ RO mixture significantly increased CLA and n-3 FA without adversely affecting rumen fermentation or productive parameters. These results have been broadened to dairy products by Vargas-Bello-Pérez et al. (2015), in which FO alone (2.6% of feed) or with hydrogenated palm oil (1.3% FO+ 1.3% palm oil in the diet) elevated the DHA and *trans-*11 C18:1 content and decreased the atherogenic index of both bovine milk and cheese (P ≤ 0.05) without compromising cheese sensory characteristics. While FO with regular plant-based oil demonstrated efficacy in dairy animals, protected plant oils and marine oils can also greatly contribute to the comprehensive lipidic profile. For example, Tóth et al. (2019) showed that a rumen-protected linseed-FO mix (at 800 g/day per animal for 10 weeks) favourably decreased the bovine milk n-6:n-3 ratio. Moreover, beyond lipids, synergistic strategies show promise. This has been demonstrated between FO and savoury plant (*Satureja khuzistanica*) by Golbotteh et al. (2024), in which dietary combination (2% for each additive at DM basis) shifted the rumen fermentation and significantly (P ≤ 0.05) increased bovine milk n-3 FA (EPA/DHA) by up to 97% and CLA by 62% at the expense of SFA.

The efficiency of marine oils has been further increased by technological processing; as an illustration, 1% of dietary low-temperature crystallized FO (at DM basis) significantly (P ≤ 0.05) increased C18:1 *trans*-11, CLA, ALA, EPA, and DHA in bovine milk compared to raw FO at the same inclusion level, making it a more efficient feed additive (Bodkowski et al., 2024). Likewise, dairy goats showed to benefit from encapsulation, as a low inclusion level of encapsulated FO improved milk DHA content and atherogenicity index without inducing MFD or altering sensory attributes (Núñez de González et al., 2020). Dietary MO also demonstrated great direct efficacy in increasing the milk DHA content, as well as reducing the atherogenic index and n-6:n-3, offering positive health benefits to consumers. Such findings have been reported in bovine milk by Angulo et al. (2013), Vahmani et al., (2013), and Sterk et al. (2012) (with 3.1% algae-blend at DM or 200 g MO/day), as well as in ewe milk by Pajor et al. (2019) and Manso et al. (2022) (with dietary MO inclusion levels ranging between 0.47 and 2.3% at feed). However, it is important to weigh the sensorial consequences associated with DHA-rich MO. In this respect, Bragaglio et al. (2015) reported that DHA-rich microalgae markedly increased bovine milk DHA content and boosted the cow immune-competence, but it significantly modified milk sensory properties, allowing panellists to discriminate the enriched milk from the control. Within this context, antioxidant co-supplementation may represent a workable strategy to tackle this sensory issue.

***To sum up***, according to the reviewed studies on cows, goats and sheep, it is evident that supplementation with LO, RO, FO, MO, or their protected mixtures successfully increases the concentrations of beneficial FAs like ALA, EPA, DHA, and CLA while improving the n-6:n-3 ratio without affecting the integrity of milk. The degree of rumen protection directly influences the success of these strategies, with encapsulated or calcium-soap forms yielding the greatest enrichment. In addition, strategies based on blending plant and marine oils demonstrated a remarkable efficacy in enriching a broad spectrum of n-3 FAs. Ultimately, marine sources consistently boost immunity and DHA; however, the maintenance of sensorial attributes of fluid milk remains a key formulation challenge. A summary of the above studies can be found in Table 4.

**Table 4:** A summary of oil sources, inclusion rates in various studies, improved animal products, and effects achieved.

Source	Levels of application and inclusion rates	Targeted animal product	Functional value effect on animal product	Authors
**Studies focused on linseed oil (LO)**				
LO or NaOH-treated linseed	CTR: no oil; 2 EXP diets: 6% LO, or 19.3% NaOH-treated linseed at feed	Mutton	both additives, with more efficacy for NaOH treatment, increased CLA, total n-3 PUFA, and PUFA:SFA ratio	(Noci et al., 2011)
LO	CTR: 0% LO: 1 EXP diet: 9% LO at feed	Rabbit meat	9% LO enriched n-3 FAs in meat, adipose tissue and liver without compromising growth; however, lipid peroxidation increased	(Trebušak et al., 2011)
LO, NaSeVI, or LO+ NaSeVI	CTR: 0% LO and 0.1 mg NaSeVI/kg feed Fixed levels across EXPs: 5% LO, and 2 mg NaSeVI/kg feed	Sheep muscles and subcutaneous fat	LO increased MUFA and PUFA levels, whereas NaSeVI co-supplementation magnified the effect on MUFA	(Czauderna et al., 2012)
LO, SO, or LO+ SO	CTR: beef tallow; 3 EXP diets: 2.9% SO, 4.8% LO, or 2.1% LO+ 2.7% SO at feed	Broiler chicken	in breast, the lowest n-6:n-3 ratio was found the LO and LO+SO groups	(Marzec et al., 2020)
LO with/without organic Se	CTR: 0% LO; 2 EXP diets: 2.5% LO, or 2.5% LO+ 0.3 mg organic Se/kg feed	Rabbit meat	SFAs decreased in EXP groups; meanwhile, PUFA increased (P ≤ 0.05), especially with organic Se co-supplementation. ALA was 3-fold higher in EXP groups compared to CTR.	(Saleh et al., 2013)
LO	CTR: 0% LO; 1 EXP diet: 5% at feed	Broiler chicken	LO increased n-3 PUFAs, while decreased n-6 FAs, and n-6:n-3 ratio in thigh and adipose (P ≤ 0.001)	Starčević et al. (2014)
LO	CTR: 2% and 3% SO at feed 3 EXP diets: LO replacing SO by 33, 67, and 100%	Broiler chicken	all LO levels significant increase in PUFA, n-3 FA, while reduced n-6:n-3 in breast and thigh muscle (P ≤ 0.05)	(Panda et al., 2015)
LO	CTR: 200 g PO/d at DM; 2 EXP diets: LO replacing PO by 50% and 100%	Beef	LO increased ALA, DHA, and n-3 PUFA, while decreased CLA and n-6 PUFA Longissimus and Semimembranosus muscles	(Suksombat et al., 2016a)
LO	CTR: 0%, 0.5%, 1%, or 1.5% PMO; 4 EXP diets: CTRs+ 2% LO	Broiler chicken	2% LO markedly increased ALA, DHA, total n-3 FAs, and PUFA, while decreased MUFA.	(Szymczyk & Szczurek, 2016)
LO+ Vit E	CTR: 3% SFO; 1 EXP diet: 3% LO+ 0.46 mg Vit E/kg feed	Rabbit meat	LO substantially increased ALA, sum of n-3 FAs and PUFA, while decreased n-6:n-3 ratio	(Matics et al., 2017)
LO	CTR: 3% SO; EXP diets: 3% LO at feed	Rabbit meat	feed supplementation with LO improves PUFA	(Dal Bosco et al., 2018)
LO	CTR: 10 g/kg vegetable oil; 2 EXP diets: 25 g/kg or 50 g/kg feed	Pork	LO increased n-3 FAs i.e., ALA, C20:3n3, EPA and total n-3 PUFA, with a decreased n-6:n-3 ratio (P ≤ 0.05)	(Leikus et al., 2018)
LO with/without lard	CTR: 5% lard; 2 EXP diets: 5% LO, or 2.5% LO+ 2.5% lard at feed	Broiler chicken	ALA was markedly higher in drumstick and thigh from LO-fed chicken.	(Milanković et al., 2019)
LO+ poultry fat +Vit E	2 CTR diets: no oil or fat+ 11 or 220 IU Vit E/kg feed; 6 EXP diets: 1% LO + 1, 3, or 5% poultry fat+ 11 or 220 IU Vit E/kg feed	Pork	1% LO + 1 to 5% poultry fat increased n-3 FA and reduced n-6:n-3 ratio. SFA in pork decreased linearly (P ≤ 0.012), while n-6 FA, PUFA, and C18:2n6 increased linearly (P ≤ 0.001).	(Huang et al., 2020)
LO+ SO	3 EXP diets: 2.3% SO+ 0.7% LO, 1.5% SO+ 1.5% LO, or 0.15% SO+ 2.85% LO at feed	Pork	highest LO inclusion rate increased the deposition of n-3 FAs	(Nong et al., 2020)
LO	CTR: 2% vegetable oil: 3 EXP diet: 2% LO, or 2% LO+ 0.5% curry/ginger/turmeric at feed	Broiler chicken	n-6:n-3 ratio decreased in all groups offered LO. The role of antioxidants on oxidative stability was not established.	(Mech et al., 2021)
LO+ GP, GSE, FH, or Vit E	CTR: 0% LO; fixed 3% LO in all EXP diets, with 10% GP, 0.0022% GSE, 5% FH, or 0.2 g Vit E/kg feed	Pork	LO increased n-3 FA in all EXP groups. High tocopherol levels were in Vit E, GSE and FH co-supplemented groups	(Bernardi et al., 2022)
PLO+ Vit E	CTR diet: 0% supplement; 1 EXP group: 140 g PLO/animal/day+ 100 mg Vit E/kg DM	Beef	PLO significantly increased n-3 FA and CLA (P ≤ 0.001)	(Corino et al., 2022)
LO	2 EXP diets at age 14-35 d: 3 and 4% at feed 2 EXP diet at age 21-35 d: 3 and 4% at feed	Broiler chicken	in the breast, n3 and PUFA significantly increased in all LO groups, with linear increase in those fed from 14-35. For 21-35 days, no difference detected between 3% and 4% group.	(Ali et al., 2023)
LO	CTR: 2% duck fat; 2 EXP diets: 2% LO at feed for 14, or 2% LO at feed for 28d	Chinese crested white ducks	supplementation of LO at 2%, fed for 28 days increased the levels of ALA, EPA, DPA significantly (P ≤ 0.05)	(Zhang et al., 2023)
LO	CTR: 0% LO; 3 EXP diets: 2.5%, 3%, or 4% LO at feed	Village chicken meat	LO increased ALA and total n-3 FA in breast and thigh (P ≤ 0.05)	(Farahiyaha et al., 2025)
LO	CTR: 0% LO; 1 EXP diet: 12.8% LO at DM	Beef	C18:3n3 and CLA levels were two-fold higher in LO group than CTR	(Roskam et al., 2025)
Linseed or LO calcium salts	CTR: no additive; 3 EXP diets: 4.2% linseed, 1.9% LO calcium salts, or 2.3% linseed+ 0.8% calcium salts+ at feed	Bovine milk	LO calcium salts and combined dietaries drastically increased ALA in milk compared to linseed and control.	(Côrtes et al., 2010)
LO, PO, OSO, LSO, or CO	CTR: no oil; 5 EXP diets: 5% DM as oil from LO, PO, OSO, LSO or CO	Bovine milk	PO significantly decreased milk fat content and short- and medium-chain fatty acids (C<16), whereas CO and LSO significantly decreased milk fat yield (0.98 and 0.86 vs. 1.14 kg/d). C18:1n9 and ALA are roughly equal in potency of milk fat depression.	(He & Armentano, 2011)
LO	CTR: 5% SFF; 1 EXP diet: 5% LO at TMR DM	Goat milk	LO significantly increased ALA and CLA in milk (P ≤ 0.05)	(Li et al., 2012)
LO or SO	CTR: no oil; 2 EXP diets: 30, 48, or 66 g LO or SO/d	Goat milk	CLA linearly increased with LO supplementation. LO decreased n-6:n-3 by 70% compared to control.	(Martínez Marín et al., 2012)
LO	CTR: 0% LO; 3 EXP diet: 3% LO, 3% LO+ 400 mg natural/synthetic Vit E/kg TMR	Ewe milk	LO increased ALA, vaccenic acid, CLA, and n-3 FAs, while decreased SFAs. Regardless of Vit E form, these alterations were not different, although Vit E source decreased oxidation.	(Gallardo et al., 2015)
LO	CTR: 500 g PO/d at DM; 2 EXP diets: LO replacing PO by 50% and 100%	Bovine milk	LO increased CLA, ALA, EPA, DHA, and n-3 PUFA in milk	(Suksombat et al., 2016b)
LO	CTR: 0% LO; 1 EXP diet: 20% LO at feed	Bovine milk	20% LO in the diet significantly lowered saturated FA (SFA) (P ≤ 0.001), increased proportions of PUFA, and total CLA than milk from the no supplementation group (P ≤ 0.001)	(Musco et al., 2022)
LO	CTR: 0% LO; 1 EXP diet: LO (10.8g/ewe/day)	Ewe milk	LO markedly increased n-3, n-6, and trans-FAs (P ≤ 0.001)	(Contreras-Solís et al., 2023)
LO, or LO+ FO	CTR: no oil; 3 EXP diets: 2.5% LO, 1% LO+ 1.5 FO, or 1.66% LO+ 2.5% FO	Bovine milk	oil combination increased CLA and n-3 FAs (ALA, EPA, and DHA), whereas decreased medium-chain saturated fats.	Thanh et al. (2023)
LO	CTR: no supplement; EXP diet: 400 g/day of LO	Bovine milk	LO decreased C10:0 - C17:1 (except for C13:0), while increased C18, C18:1, as well as CLA, ALA and PUFA (P ≤ 0.05)	(Gheno et al., 2024)
LO, SFO or SFF	CTR: 0% oil; 3 EXP diets: 4.1% LO, SFO, or SFF at DM	Bovine milk	SFO and SFF increased CLA and LA, while LO induced the accumulation of ALA, EPA and total n-3 FAs	
LO, SO, or SFO	CTR: 0% oil; 3 EXP diets: LO, SO, or SFO at 5% in feed	Egg	LO significantly increased ALA, DHA, total n-3 PUFAs, while decreased n-6:n-3 ratio	(Oliveira et al., 2010)
LO	CTR: 0% LO; 4 EXP diets: 0, 1.0, 2.0, and 3.0% at feed	Quail egg	LO significantly increased ALA and n-3 FA, and depicted low n6:n3 ratio	(Citil et al., 2011)
LO	CTR: 3% SO; 1 EXP diet: 3% LO at feed	Egg	LO positively affected n-3 FA (C18:3n3) (P ≤ 0.05). ALA in the CTR was 0.82 g/100 g and in EXP 5.63 g/100 g	(Hudečková et al., 2012)
LO	CTR: 0% LO; 4 EXP diets: 1%, 2%, 3%, or 4% LO at feed	Egg	LO did not alter the yolk weight. LO at > 3% inclusion rate improved ALA, EPA, while decreased n-6:n-3 ratio after 5 week (P ≤ 0.0001), and remained unaltered afterwards	(Petrović et al., 2012)
LO or PMO	CTR: no oil; 2 EXP diets: 3% of LO or PMO at feed	Egg	LO and PMO significantly increased (P ˂ 0.01) MUFAs and n-3 FA, while decreased SFAs and LA. n-6:n-3 ratio decreased in LO group compared to control (4.5 vs. 32.4).	(Herkeľ et al., 2016)
LO	CTR: no oil; 2 EXP diets: LO at 3% or 5%, algae at 7.5 or 10%, or 3% LO+ 7.5% algae at feed	Egg	Eggs from hens fed 5% LO averaged 637 mg of ALA, EPA, and DHA. The combined strategy results in eggs cover 31 to 55% of daily recommendations of these n-3 FAs	(Kim et al., 2016a)
LO	CTR: 1.5% of CO or LO; 4 EXP diets: 1.5% of LO or CO+ 3 or 5% of DFA at feed	Egg	n-3 FAs increased by LO supplementation; however, DAF further improved DFA improved (P ≤ 0.05) n-6:n-3 ratios in egg yolk and plasma from 13 to 23 and 7 to 13, respectively	(Kim et al., 2016b)
LO or milled linseed	CTR: 0% LO or milled linseed; 10 EXP diets: flaxseed oil diets or milled flaxseed diets at 0.5, 1.0, 2.0, 3.0, or 5.0% at feed	Egg	LO and milled flaxseed resulted increased ALA, EPA, and DHA in egg yolk; however, FA depositions, especially EPA and DHA, were double in LO group (P ≤ 0.01)	(Ehr et al., 2017)
LO	3 levels: 1, 3, and 5% of linseed LO at feed	Egg	ALA, and DHA, in egg yolk, were significantly higher when 3% or 5% LO (P ≤ 0.05). Optimal enrichment at 3% supplementation	(Duan et al., 2021)
LO	CTR: no oil; 4 EXP diets: 0.2, 0.4, 0.6, or 0.8% at feed	Egg	LO significantly increased (P ≤ 0.05) n-3 FA and DHA were observed, but α-linolenic acid and eicosapentaenoic acid were not altered	(Lee et al., 2021)
LO	CTR: 0% LO; 3 EXP diets: 2%, 3% or 4% LO+ 3000 or 10000 IU vitamin A/kg feed	Egg	ALA and total n-3 increased with LO administration. n-3 PUFA were increased linearly (P ≤ 0.001) in the egg-yolks	(Ahmad et al., 2022)
LO+ SO	CTR: 5% SO+ 0% LO; and 5 EXP diets: 4% SO+ 1% LO, 3% SO+ 2% LO, 2% SO+ 3% LO, 1% SO+ 4% LO, or 0% SO+ 5% LO at feed	Egg	higher LO levels linearly increased C18:3n3 content of yolk	(Altaçli et al., 2022)
LO or SO	CTR: no oil; EXP diets: LO or SO at 2.5% at feed	Egg	n-3 FA content increased in yolk from experimental groups. ALA and DHA were significantly highest in LO group.	(Batkowska et al., 2021)
LO+ CO+ FO	CTR: 0% oil; 5 EXP diets: 5% CO, 5% LO, 2% CO+ 3% LO, 3% CO+ 2% FO, or 3% LO+ 2% FO at feed	Egg	egg total lipid was not affected by oil sources, but egg triglycerides was altered.	(Attia et al., 2022b)
**Studies focused on rapeseed oil (RO)**				
RO	CTR: 0% RO; 3 EXP diet: 5% LO, 10% LO, or 15% RO at feed	Broiler chicken	RO increased ALA and C18:1n9 (P ≤ 0.05), while slightly decreased LA (P ≤ 0.01)	(Gallardo et al., 2012)
RO or LO	CTR: 2% SFO; 2 EXP diets: 2% of RO or LO at feed	Pork	both LO and RO increased n-3 FAs, whereas the most favorable ratio of n-6:n-3 FA was observed in LO group	(Kralik et al., 2010)
RO, LO SO	3 EXP diets: 0.99, 4.4, or 7.31 of n-6:n-3 ratio	Turkey meat	0.99 dietary n-6:n-3 ratio increased ALA, EPA, and total PUFA in meat (P ≤ 0.01), while decreased n-6/n-3 ratio	Jankowski et al. (2012)
RO or LO	CTR: 0% oil; 4 EXP diets: 5% RO, 10% RO, 4% LO, 8% LO at feed	Ostrich meat	dietary oils, especially LO, increased ALA and total n-3 PUFAs, while decreased n-6:n-3 ratio (P ≤ 0.01). Only LO improved PUFA/SFA	(Polawska et al., 2013)
RO	CTR: 4% SO; 1 EXP diet: 4% RO at feed	Pork	RO dietary inclusion significantly (P ≤ 0.05) increased ALA (+2.38%), while decreased n-6:n-3 (P ≤ 0.001)	(Okrouhlá et al., 2018)
RO	CTR: 0% RO; 1 EXP diets: 10% RO at feed	Duck meat	LA, EPA and DHA linearly increased while n-6:n3 ratio reduced with the supplementation duration	(Shahid et al., 2019)
RO	CTR: 0% RO; 1 EXP diet: 3% RO at feed	Mutton	3% RO in diet substantially (P ≤ 0.001) improved MUFA and PUFA profile. It also increased CLA and EPA; better n-6:n-3 ratio	(Quiñones et al., 2019)
RO	CTR: 4% SO at feed for 2, 4, and 6 weeks; 1 EXP diet: 4% RO at feed for 2, 4, and 6 weeks	Pork	RO supplementation 4 or 6 weeks before slaughter has a positive impact on the n-6:n-3 PUFA ratio and an increase in C18:3n3	(Vehovský et al., 2019)
RO	CTR: 1.5%, 3%, or 4.5% PO at feed: 3 EXP diets: RO replacing PO by 25%, 50%, and 100%	Broiler chicken	100% RO replacement increased n-3 FA concentrations, which were significantly increased in breast and thigh muscles (P ≤ 0.001)	(Chinnasamy et al., 2022)
RO, LO or SO	3 EXP diets: 5% RO, LO or SO at feed	Broiler chicken	5% LO markedly elevated ALA and total n-3 FAs (highest values P ≤ 0.001), although RO also markedly increased these components. Both LO and RO improved health indices	(Kralik et al., 2025)
RO or SO	CTR: no oil; 2 EXP diet: 0.85% RO or SO at DM	Bovine milk	both oils decreased SFAs, while increased MUFAs and PUFA, including CLA	(Altenhofer et al., 2014)
RO	CTR: 0% RO; 2 EXP diets: 4.9% crushed full-fat rapeseed, or 2.2% RO at DM	Bovine milk	reduced SFAs and increased long-chain and unsaturated FA yields respectively (P ≤ 0.001)	(Hoffmann et al., 2016)
RO, SO or PNO	CTR: no oil; 3 EXP diets: 2% RO, SO, or PNO at feed	Bovine milk	dietary oil increased CLA, C18:1n9, ALA and total MUFAs in milk. Milk yields were also increased. No marked differences in milk n-3 FAs between different dietary oils	Dai et al. (2011)
RO	CTR: 0% RO; 1 EXP diet: 5% RO at DM	Bovine milk	RO increased CLA, ALA and total PUFA (P ≤ 0.01)	(Razzaghi et al., 2022)
RO or PO	CTR: 0% RO or PO; 2 EXP diets: 8% RO or PO at DM	Goat milk	PO increased C16:0, while RO increase CLA and ALA. RO had the lowest free FAs, and, thus, it did not compromise flavor like in PO	(Inglingstad et al., 2017)
RO	CTR: 0% oil; 1 EXP diet: 3% RO at feed	Egg	elevation in ALA proportion (0.81% in the RO group to 0.17% in the CTR)	(Omidi et al., 2015)
CLA+ RO	8 EXP diets: 1% LO+ 1.4% OO or RO+ CLA at 0, 0.5, 0.75, 1.0% and SO to reach 1.54% at feed	Egg	CLA in diet significantly increased CLA in the egg yolk	(Franczyk-Żarów et al., 2019)
RO, SO, SFO, CO or HSO	5 EXP diets: each oil used separately at 3.7% inclusion rate in feed	Egg	hemp oil increased the PUFA content in the yolk, similar to SO, CO, and SFO, but unlike the latter, it also enriched the n-3 fatty acids in the yolk (P < 0.05). RO group depicted the highest MUFA level, and also increased total n-3 FA	(Kanbur et al., 2022)
LO with/without RO	CTR: 5% SO at feed; 2 EXP diets: 2% SO+ 0.5% algae+ 1.2% RO+ 1.3% LO, or 1.5% SO+ 1% algae+ 1.2% RO+ 1.3% LO at feed	Egg	in yolk, significant increases (P ≤ 0.05) in n-3 FA and DHA were observed in EXP groups. 1% algae displayed the highest DHA (2-fold) compared to 0.5% algae	(Kralik et al., 2024)
**Studies focused on fish oil (FO)**				
FO, LO or SO	CTR: 5% PO; 3 EXP diets: 5% FO, LO, or SO at feed	Broiler chicken	FO remarkably increased EPA, DPA, and DHA in breast, thigh and liver. SO increased LA (2-3 fold), while LO drastically increased ALA (4-20 fold)	(Poureslami et al., 2010)
FO	CTR: 0% FO; 3 EXP diets: 1.5%, 3%, or 6% FO at feed	Broiler chicken	FO increased ALA, EPA and DHA in breast and thigh meat	(Saleh et al., 2010)
FO, LO or CLA	3 EXP diets: 2% FO, LO or CLA	Broiler chicken	dietary FO had the highest EPA and DHA deposition (P ≤ 0.05). Dietary CLA elevated breast CLA isomers, while LO induced deposition of C18:1n9, and ALA. Lower oxidation was found in CLA group	(Narciso-Gaytán et al., 2011)
FO, CLA, LO, or their mixtures	3 EXP diets: 2% for each supplement; 2 EXP diets: 1% CLA+ 1% LO, or 1% CLA+ 1% FO at feed	Broiler chicken	CLA and FO group resulted in a lower AA concentration in both breast and thigh muscles than FO or CLA+ LO groups (P ≤ 0.05). CLA and LO showed higher C18:3n3 level compared to FO group	(Shin et al., 2011a)
CLA, LO, FO, or their combinations	5 EXP diets: 2% CLA, 2% LO, 2% FO, CLA+ LO (1:1; 2%), or CLA and FO (1:1; 2%) at feed	Broiler chicken	the n-3 and n-3 to n-6 FA ratio in both breast and thigh meat increased in the combination group compared with CLA group, while SFA content decreased (P ≤ 0.05). The group fed CLA+ FO displayed the highest DHA content	(Shin et al., 2011b)
FO	CTR: no oil; 4 levels in EXP diets: 5.5%, 4%, 2.5% or 1.5% at feed	Broiler chicken	FO increased the concentration of n-3 FA, especially EPA and DHA, in the breast	(Maroufyan et al., 2011)
FO	CTR: 0% FO; 3 EXP diet: 1%, 2%, or 3% FO at feed	Broiler chicken	n-3 FAs, especially EPA and DHA, were increased, alongside n-3:n-6 ratio. 2% FO was the most favorable by panelists	(Aghaei et al., 2012)
FO, PO, CLA, SO, FO+ SO,CLA+ SO, or CLA+ FO	CTR: 0% oil; 3 EXP diets: 7% for single fats and 3.5%+ 3.5% for dual mixed fats at feed	Broiler chicken	CLA+ FO and CLA+ SO resulted in high n-3 PUFA and CLA, respectively	(Royan et al., 2013)
LO, LO+FO, or FO	CTR: 3% palm oil; and 3 EXP diets: 3.3% LO, 10.7% LO+ 4% FO, or 3.3% FO at feed	Mutton	EPA and DHA were highest in FO group, whereas ALA was highest in LO and LO+FO groups. FO negatively affect performance and meat quality	(de la Fuente-Vázquez et al., 2014)
FO	CTR: 0% FO or 4% SO at TMR; 3 EXP diets: FO replacing SO at 6.5%, 12.5% and 18.8% rates	Mutton	FO administration significantly increased ALA, EPA and DHA (P ≤ 0.001)	(Ferreira et al., 2014)
FO, NaSeVI or Se-yeast	CTR: 4% SFO; 4 EXP diets: 12 ppm lycopene, 2% SFO+ 2% FO,0.25 mg NaSeVI or Se-yeast/kg diet	Broiler chicken	FO and Se-yeast had the highest PUFA levels, especially n-3 long-chain PUFA	(Rozbicka-Wieczorek et al., 2014)
FO	CTR: 3% RO; 2 EXP diets: 2% RO+ 1% FO, 2% RO+ 1% FO+ 0.1% CA at feed	Mutton	FO and CA inclusion increased CLA, total n-3 FAs, and n-3:n-6 ratio	(Jaworska et al., 2016)
FO+ LO or RO	CTR: no oil; 3 EXP diets: 1% FO, 1% FO+ 6% LO, 1% FO+ 5.8% RO at feed	Pork	Highest n-3 FA content and the lowest n-6:n-3 FA was in the meat from FO+LO (P ≤ 0.001); however, the lowest omega ratio in FO group	(Konieczka et al., 2017a)
FO or PNO	CTR: no oil; 4 EXP diets: 2% PNO, 1% FO, 1.5% FO, or 2% FO at feed	Broiler chicken	FO markedly increased EPA and DHA in meat and liver compared to control and PNO groups	(Muhammed et al., 2015)
FO, RO, SO, or their mixtures	CTR: no oil: 3 EXP diets: 3% FO, RO, or SO 3 EXP diets: 1.5% FO+ 1.5% RO/SO, or 1.5% RO+ 1.5% SO	Mutton	lambs fed with FO had the highest the EPA, DPA, and DHA (P ≤ 0.01). SFA and n-6:n-3 were markedly decreased by dietary FO inclusion	(Parvar et al., 2017)
FO or LO	CTR: 4-4.5% So at feed; 4 EXP diets: FO or LO replacing SO at 25% and 50% rats	Broiler chicken	breast ALA content was highest with dietary LO, while FO increased EPA, DPA and DHA (P ≤ 0.05)	(Ibrahim et al., 2018)
FO	CTR: 0.75% lard oil; 1 EXP diet: 1.5% Optomega-50	Rabbit meat	FO led to an FA profile with a lower n-6:n-3 ratio (2.20, P ≤ 0.001) in the muscle and perirenal fat against 7.29 in the CTR	(Rodríguez et al., 2019)
FO	3 EXP diets: 0, 1, or 2.1% FO at feed	Beef	FO increased CLA (P ≤ 0.01) and n-3 (P ≤ 0.01) FAs and reduced n-6:n-3 (P ≤ 0.01) more in the group fed 2.1% supplemental FO	(Zakariapour Bahnamiri et al., 2019)
FO	CTR: 4% SO; and 3 EXP diet: all received 4% FO +nothing, or 15 mg lycopene, Se-yeast, or both lycopene and Se-yeast/kg feed	Rabbit meat	all FO-fed groups showed markedly higher PUFA, while lower n6:n3 ratio	(Danuta et al., 2020)
n-3 FO/n-6 SO	2 EXP diets: n-6 PUFA SO, or n-3 FA enriched FO	Beef	FO group had greater n-3 FA level than for SO or CTL groups (P ≤ 0.05)	(Byrne et al., 2021)
Protected FO	CTR: not oil; 2 EXP diet: 1% or 3% microencapsulated-FO at feed	Mutton	ALA, EPA and DHA in *Longissimus lumborum* markedly (P ≤ 0.05) increased with microencapsulated-FO administration	(De Marzo et al., 2023)
FO or SO	CTR: 0% FO or SO; 4 EXP diets: 2.5%, or 5% FO or SO at feed	Goat meat	FO significantly increase ALA, EPA and DHA, while decreased C18:0 and C18:1n9 (P ≤ 0.05)	(Sondakh et al., 2025)
FO or LO with/without SeMet	CTR: 3% SFO; 4 EXP diets: 3% LO, 3% FO, 3% LO+ 0.3 mg SeMet/kg feed, or 3% FO+ 0.3 mg SeMet/kg feed	Pork	FO and LO significantly increased PUFA and decrease n-6:n-3 (P ≤ 0.05). Synergistic interaction between oil and SeMet elevated ALA, C20:3n3, and n3 FA	(Yin et al., 2025)
FO	2 EXP diet: 55.5 g SO or 11.1 g FO/day/animal	Goat milk	FO significantly increased CLA, EPA, and DHA in milk	(Tsiplakou & Zervas, 2013b)
FO+ SO	CTR: no supplement; 1 EXP diet: 23.6 g SO and 4.7 g FO/kg DM	Ewe milk	Mixed supplement increased CLA, EPA, DHA in milk	(Tsiplakou & Zervas, 2013a)
FO with or without extruded linseed	CTR: no additive; 2 EXP diets: 530 g linseed/d, or 340 g linseed+ 39 g FO/d	Goat milk	linseed increase C18:1 and ALA:LA ratio, while FO increased EPA and DHA, associated with depletion in milk fat content	(Bernard et al., 2015)
FO+ RO	CTR: 0% FO or RO; 1 EXP diet: 180 g FO+ 180 g RO/d	Bovine milk	oil supplement had more (P ≤ 0.05) C18:2 c9t11 and C18:2 t10c12, by 30% and 38%, respectively	(Cieślak et al., 2015)
FO, LO, SFO or their combination	CTR: no oil; 3 EXP diets: 3% LO, 1.5% SFO+ 1.5% FO, or 1% LO+ 1% SFO+ 1% FO at concentrate	Bovine milk	LO+ FO had the lowest n-6:n-3 ratio however, the LO+SFO+FO improved the related health indexes in milk	(Thanh & Suksombat, 2015)
FO and/or PO	CTR: no oil; 2 EXP diets: 2.6% FO, 1.3% FO+ 1.3% PO at feed	Bovine milk and cheese	for milk and cheese, both EXP groups showed markedly high PUFA, with higher EPA and DHA deposition rates in FO group	(Vargas-Bello-Pérez et al., 2015)
FO or SO	CTR: no oil; 2 EXP diets: 3% FO or SO at DM	Bovine milk and ice cream	SO increased LA, while CLA, ALA, EPA and DHA were increased by FO supplementation. None of oils compromised production or sensory qualities of milk and ice cream	(Vargas-Bello-pérez et al., 2019)
Encapsulate FO	CTR: 0% FO; 1 EXP diet: 1.14 g/kg concentrate	Goat milk	FO did not affect milk production, sum of PUFA, but it decreased atherogenicity index (P ≤ 0.05)	(Núñez de González et al., 2020)
FO or CFO	2 EXP diets: 1% of FO or CFO at DM	Bovine milk	CFO significantly increased C18:1 trans-11, C18:2 cis-9, trans-11, ALA, EPA, and DHA, and reduced SFAs compared to the FO group (P ≤ 0.05)	(Bodkowski et al., 2024)
FO or SO	2 EXP diets: 2% of FO or SO at DM	Bovine milk	MUFAs and PUFAs (especially DHA, EPA, C18:3n3 and CLA) were higher in FO than SO groups	(Golbotteh et al., 2024)
FO and flaked linseed	CTR: no supplement; 2 EXP diets: flaked linseed at 3.88% DM, and FO at 2.64% at DM	Goat milk	flaked linseed and FO increased PUFA levels, with CLA and C18:3n3 highest in flaked linseed, whereas EPA and DHA highest in FO group. DHA accumulation is limited (non-linear incorporation)	(Moya et al., 2023)
FO	CTR:0% LO or FO; 2 EXP diets: 1.5% FO, or 1.5% FO+ 10% linseed at feed	Egg	EPA and DHA were significantly highest with FO feeding, whereas linseed dropped these levels; yet, the control depicted the lowest levels	(Yalçın & Ünal, 2010)
FO, LO or RO	CTR: no oil; 4 EXP diets: 5% FO, LO, or RO	Egg	FO drastically increased EPA and DHA. RO was most effective in enriching C18:1, while ALA was most enriched by LO. LO resulted in the lowest n-6:n-3 ratio, followed by FO and LO	(Aro et al., 2011)
FO, LO, RO or SFO	6 EXP diets: 1.5% or 3% of FO, LO, RO, or SO at feed	Egg	FO increased DHA in yolk, while LO and RO targeted ALA (p < 0.01). FO affected sensory properties	Ceylan et al. (2011)
FO	CTR: 0% FO; 1 EXP diet: 10% FO at feed	Egg	EPA and DHA were drastically increase, which was up to 155.98-201.53%. FO did not compromise egg texture, aroma, flavor, or appearance	(Sugata et al., 2020)
FO, LO or RO	CTR: no oil; 9 EXP diets: 1%, 2%, or 3% FO, LO, RO, or their combinations	Egg	Oil decreased C16:0 and C18:0, while increased ALA, EPA, and DHA, proportional variations were responses to oil FA profiles. No sensory alteration detected in eggs	(Valavan et al., 2013)
FO	CTR: 0% supplement; 1 EXP diet: 1.5% FO at feed	Egg	EPA and DHA were only detected in FO group	(Coorey et al., 2015)
FO	CTR: 5% SFO; 4 EXP diet: 1.25% FO +3.75 % SFO, 2.5% FO+ 2.5% SFO, 3.75% FO+ 1.25% SFO, or 5% FO at feed	Egg	significant elevation (P ≤ 0.05) in ALA level in yolk by 30.5% was observed in the 3.5% fish oil group	(Saleh, 2013)
FO+ LO or RO	CTR: 5% SO; 4 EXP diets: 1.5% RO+ 3.5% FO, 3.5% LO+ 1.5% FO, 1.5% RO+ 3.5% FO, or 3.5% RO+ 1.5% FO at feed	Egg	LO+FO groups showed efficient deposition of n-3 FAs in yolk, which significantly decreased n-6:n-3 ratio, compared to control and RO+FO groups	(Kralik et al., 2020)
FO+ SO	CTR: 5% SO; 4 EXP diets: 0.3% FO+ 4.7% SO, 0.6% FO+ 4.4% SO, 0.9% FO+ 4.1% SO, 1.2% FO+ 3.8% SO, or 1.5% FO+ 3.5% SO at feed	Egg	FO significantly elevated EPA, DHA, total n-3 FAs, while decreased n-6:n 3	(Kralik et al., 2021)
FO, SO, Se-yeast, Vit E, lutein (L)	2 Exp diet: 5% SO+ 0.32 mg Se-yeast+ 25.20 mg Vit E+ 20.5 mg L/kg feed, or 1.5% FO+ 1.5% RO+ 2.0% LO+ 0.47 mg Se-yeast+ 125.2 mg Vit E+ 120.5 mg L/kg feed	Egg	concentrations of n-3 FA, Se, and Vit E significantly increased in eggs	(Kralik et al., 2023)
FO or krill oil	CTR: 0% FO and krill; 3 EXP diets: 1.5% FO, 1.5% krill oil, or 3% krill oil at feed	Egg	DHA and EPA from FO were more efficiently incorporated than krill oil. The retention efficiency of n-3 LC-PUFAs was negatively dependent on dietary levels	(Zhao et al., 2021b)
FO+ LO	CTR: 5% SO; 2 EXP diet: 3.5% LO+ 1.5% FO, or 3% LO+ 2% FO	Egg	LO supplementation target al.A level (P ≤ 0.01). FO supplementation increased n-3 PUFAs, especially DHA (P ≤ 0.001), in eggs. It reduced n-6:n-3 ratio.	(Radanović et al., 2023)
FOCS	CTR: 0% FOCS; 3 EXP diets: 0.75%, 1.5% and 2.25% at feed	Egg	in yolk, n-3 FAs increased and SFAs decreased in FOCS group (P ≤ 0.05), which were linearly responsive to FOCS inclusion rates	(Ghaderi-Chaparabad et al., 2025)
**Studies focused on microalgae oil (MO)**				
MO	CTR: 0% MO; 1 EXP diet: 2% MO at feed	Mutton	intramuscular fat of MO-fed lambs had enhanced levels of C22:6n3 (3.35%) and total n-3 FA (5.71%), as compared to that of CTR lambs (0.25% and 1.23%, respectively)	(Díaz et al., 2017)
DHA Gold or Formaldehyde-treated linseed	CTR: no additive; 2 EXP diets: inclusion rates used to each 11% DHA at concentrate	Bovine milk	treated linseed induced deposition of ALA (P ≤ 0.001), while DHA Gold induced MUFA deposition in milk (P ≤ 0.01)	(Sterk et al., 2012)
DHA-enriched algae with/without LO or SFO	CTR: 3.1% PPF at DM; 2 EXP diet: 3.1% LO-algae, or 3.1% SFO-algae	Bovine milk	milk from SFO and LO had more phospholipids. LO-algae improved FA profile, as it increased healthier CLA isomers	(Angulo et al., 2013)
MO or FO	CTR: 0% MO or FO; 2 EXP diets: 200 g MO or FO/d	Bovine milk	FO and MO reduced C16, while increased n-3 PUFAs, but increased trans C18:1 content in milk	(Vahmani et al., 2013)
DHA Gold+ Vit E	2 EXP diets: 136 g of DHA/day; 136 g of DHA+ 2000 UI of Vit E/day	Bovine milk	EXP diets elevated DHA levels (P ≤ 0.05) in milk fat. However, panelists were able to distinguish enrich milk from non-enriched samples	(Bragaglio et al., 2015)
MO	CTR: 1500 g alfalfa hay+ 600 g of concentrate 3 EXP diets: CTR+ 15 g microalgae/head/day, pasture+ 600 g concentrate, or pasture+ 600 g concentrate+ 15 g MO/head/day	Bovine milk	MO significantly (P ≤ 0.01) increased the C22:6n3 levels in milk fat in all the MO groups (0.40% in EXP 1 and 0.39% in EXP 3). Additionally, the n-6:n-3 ratio was significantly favorable in the supplemental microalgae groups (1.25 and 1.37 in EXP 1 and 3, respectively)	(Pajor et al., 2019)
MO, LO, PO or calcium soap-LO	CTR: 0% oil; 3 EXP diets: 2.3% MO, LO, PO or calcium soap LO at feed	Ewe milk	MO was the most effective additive to increase n-3 FAs	(Manso et al., 2022)
LO, MO, FO and DHA Gold	CTR: no oil; 4 EXP diets: for each 120 mg n-3 FA/100 g feed	Egg	different n-3 FA sources lead to different n-3 LC-PUFA enrichment efficiencies, with highest EPA and DHA deposition in FO and MO groups	(Lemahieu et al., 2015)
FO and MO	CTR: no FO or MO; 6 EXP diets: each oil type at 0.20%, 0.40% or 0.60% total omega-3/kg feed	Egg	n-3 PUFA in the yolk increased (P ≤ 0.0001) linearly. The MO DHA enrichment level was 3-fold greater than FO	(Neijat et al., 2016)
MO or FO	CTR: no supplement; 6 EXP diets: DHA at 1.25, 2.50, or 5.00 mg/g feed derived from MO or FO	Egg	DHA elevated in MO and FO groups, and linear increment observed in n-3 FA	(Feng et al., 2020)
MO	CTR: 0% MO; 3 EXP diets: 0.5%, 1%, or 2.5% MO at feed	Egg	DHA increased quadratically (R^2^ = 85.28, P ≤ 0.001)	(Moran et al., 2020)
n-6/n-3	3 ratios of EXP diets: 16.7:1; 9.3:1; and 5.5:1	Egg	hens fed the 9.3:1 n-6/n-3 ratio had significant enriched PUFA levels	(Attia et al., 2022a)
Algae or LO	8 EXP diets: LO or FO provided 0.2%, 0.4%, 0.6%, or 0.8% of total dietary n-3 PUFA	Egg	LO induced ALA deposition, whereas the algal increased EPA and DHA.	(Li et al., 2023b)
MO	0.25% OPAO, 0.76% OPAO, 0.23% CAO, or 0.69% CAO	Egg	algal oils enriched eggs with DHA linearly and quadratically (P ≤ 0.01)	(Maina et al., 2023)

Abbreviations: ALA, α-linolenic; CA, carnosic acid; CAO, Crude algal oil; CFO, low temperature crystallization fish oil; CO, corn oil; CTR, control diet; DFA, defatted microalgae; DHA, docosahexaenoic acid; DM, dry matter; DPA, docosapentaenoic acid; EPA, eicosapentaenoic acid; EXP, experimental diet; FA, fatty acid; FH, fish hydrolysate; FO: fish oil; FOCS, fish oil calcium salt; GSE, grape seed extract; GP, grape pomace; HSO, hemp seed oil; L, lutein; LO, linseed oil; LSO, high-linoleic safflower oil; MO, microalgae oil; MUFA, monounsaturated fatty acids; NaSeVI, sodium selenate; OO, olive oil; OPAO, OmegaPro; OSO, high-oleic safflower oil; PLO, protected linseed oil; PMO, pumpkin oil; PMR, partial mixed ration; PNO, peanut oil; PO, palm oil; PUFA, polyunsaturated fatty acid; RO, rapeseed oil; SeMet, selenomethionine; SFA, saturated fatty acid; SFF, safflower oil; SFO, sunflower oil; SO, soybean oil; TMR, total mixed ration; VFA, volatile fatty acid.

#### 3.4.3 Key considerations and future direction

**Fig. 10:**
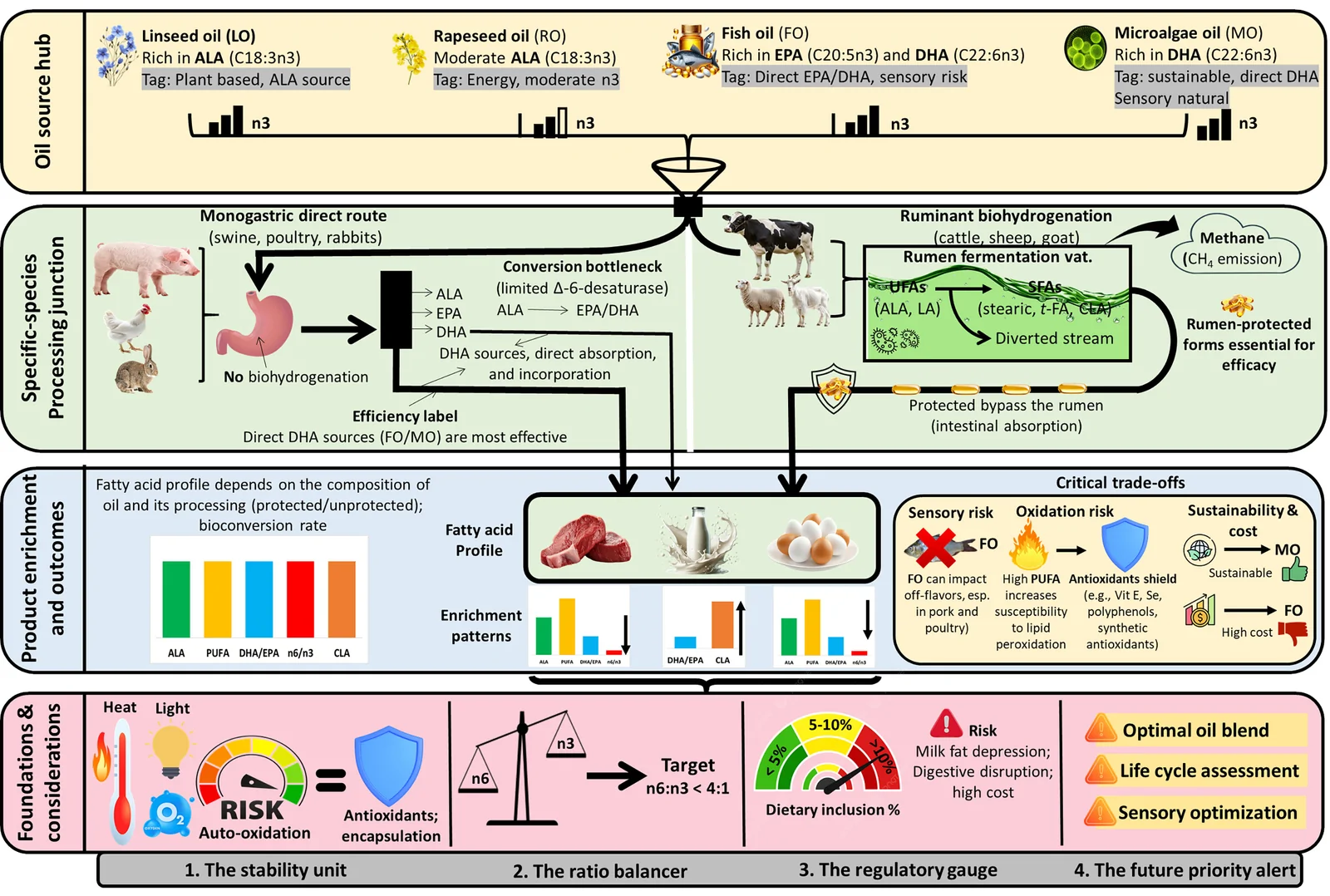
The metabolic pathway of omega-3-rich dietary oils (derived from linseed, rapeseed, fish and algal sources) for enriching animal products.

The inclusion of PUFAs, particularly n-3 PUFAs, in animal diets is increasingly prioritized for their health-promoting effects and potential to enrich functional animal-derived foods. Nevertheless, it is important to understand that both lipid requirements and metabolism vary across animal species (extensively discussed earlier in Section 3.4.1); hence, appropriate consideration of the inclusion rate is crucial (see Fig. 10).

##### 3.4.3.1 Auto-oxidation of lipids

It is well known that lipids with a high degree of unsaturation are prone to ***oxidative degradation*** (accelerated by prolonged storage, high heat, intense light, and poor packaging), jeopardizing diet quality and animal product stability through off-flavours, colour instability, and reduced shelf life (Shah et al., 2018). In feed, for instance, dietary oxidation triggers lipid rancidity and degrades vitamins, pigments and amino acids, as well as suppresses feed intake and risks nutrient deficiencies that ultimately impair metabolism and productivity. This situation underscores the technical challenge of elevating the n-3 PUFA level in the diet, which introduces a vulnerability that necessitates multi-nutrient strategies.

In poultry and pigs, FO and LO supplementation elevates the risk of post-mortem lipid oxidation (Lee et al., 2022; Sarker et al., 2020), deteriorating sensorial traits, muscle protein functionality, and overall product acceptability. This pattern has been practically demonstrated by Lee et al. (2022), who found yolk malondialdehyde level quadratically increased with soluble LO inclusion, as well as Dal Bosco et al. (2018), who noted thiobarbituric acid reactive substances (TBARS) significantly increased in rabbit loins fed LO during retail display. For dairy cows, PUFA-rich diets (e.g., high inclusion of oilseeds or marine sources) increase the susceptibility of milk fat to oxidation during storage or light exposure (Kouba & Mourot, 2011), while ruminal biohydrogenation alters PUFA bioavailability, necessitating rumen-protected forms (Jenkins & Bridges, 2007).

To ***counteract the auto-oxidation*** of lipids in both feed and animal products, several strategies have been developed. The dietary inclusion of natural antioxidants (e.g., Vit E, Se, lutein, rosemary extract, plant polyphenols, etc.) and/or synthetic antioxidants (e.g., ethoxyquin, BHT and BHA) has shown effective in reducing oxidative damage (Desbruslais & Wealleans, 2022a; Kralik et al., 2023; Mughal et al., 2021a). Importantly, Vit E requirement is not fixed but it scales with dietary and tissue PUFA content, underscoring the stoichiometric relationship between substrate and radical neutralizer. From a practical approach, the addition of 10 mg α-tocopherol per gram of supplemental n-3 PUFA has been suggested, aiming at maintaining oxidative stability in enriched products (Fritsche & Johnston, 1990). It is impossible to avoid this stoichiometric demand, as demonstrated in pork and quail meat (Bernardi et al., 2022; Salahi Kojur et al., 2026), in which improved oxidative stability and reduced TBARS were recorded. This proportional relationship underpins Pillar 3 (proposed in Section 4): scale co-antioxidants to PUFA enrichment targets, wherein a scaling approach is important rather than fixed basal levels. Encapsulation technologies and feed formulation adjustments (e.g., controlling PUFA ratios and antioxidant levels) have also revealed potential for stabilizing tissues rich in lipids (Akonjuen & Aryee, 2023). Moreover, dietary rumen-protected methionine and lysine, alone or in combination, have also been established to increase the antioxidant system and decrease the oxidative rate (Mavrommatis et al., 2021), underscoring the role of nutrients (macro- or micro-nutrients) in mitigating oxidative stress and enhancing product qualities.

##### 3.4.3.2 Sensorial, economic and environmental considerations

Consumer acceptance of n-3-enriched animal products is generally positive, driven by rising health awareness. FO, while very effective, often imparts undesirable flavours, resulting in less desirable products. These sensorial challenges, such as fishy flavour or textural changes, can hinder adoption unless oxidative stability is ensured (Ganesan et al., 2014). This is likely a persistent barrier, as demonstrated with odourless dietary FO that altered rabbit meat sensorial attributes (Danuta et al., 2020), while MO preserves egg flavour and acceptability far better than FO at equal DHA levels (Feng et al., 2020). Therefore, ***future studies*** need to focus on the effects of long-term feeding of different oil levels on n-3 levels and the sensorial qualities of the product, as well as on the development of innovative processes and strategies to mitigate undesirable sensory changes. However, regardless of the oil source, both transparent communication of health benefits and strategies involving clean-label antioxidants are critical to bolstering consumer trust and product marketability. In this context, according to Jaworska et al. (2016) and Jankowski et al. (2012), Se-yeast alone or with Vit E demonstrated efficacy in maintaining sensorial properties of lamb and turkey meats in dietary strategies based on carnosic acid and LO supplementation.

The ***environmental*** concerns associated with FO sourcing are factual barriers, leading to the assessment of alternative sources. MO and rumen-protected LO present more sustainable and sensorially neutral alternatives. Concerning MO, despite its immense contribution to nutrition, quality and the environment, a crucial consideration for market viability is the potential for long-chain PUFA enrichment to increase the price of products (Shah et al., 2018), which may compromise the market demand power. Therefore, the sustainability, safety, cost-effectiveness (e.g., large-scale microalgae production), and full life cycle assessment remain key areas for future research. It is important to note that emissions are also interconnected with environmental considerations; as an illustration, dietary LO or RO is shown to reduce CH_4_ emissions (between 7% and 19%) while simultaneously enriching n-3 FAs in meat (Bayat et al., 2018; Roskam et al., 2025), reflecting a dual-benefit climate strategy. With respect to MO, investigating optimal microalgal species and their composition for the purpose of oil production can assist in achieving these goals.

##### 3.4.3.3 Dietary fatty acids: interactions and balanced ratios

The oil source plays a substantial role in modifying the FA composition of animal products. For instance, the bioconversion efficiency of ALA to its elongated and more bioactive forms, like EPA and DHA, is limited in animals and humans, whereas the desired functional benefits for human health are largely attributed to these elongated bioactive forms. Thus, from a ***practical perspective***, mixing plant oils (LO or RO) with marine oils (MO and/or FO: high in EPA and DHA) would be beneficial for achieving high n-3 PUFA concentrations and bioactive values. Kralik et al. (2020) confirmed these findings, demonstrating that a combination of LO and FO yielded a more efficient deposition of n-3 PUFAs and a narrower n-6:n-3 ratio in eggs compared to RO and FO. However, this area of oil mixtures requires further investigation, as certain oils, such as high-oleic SO and high-oleic peanut oils, may inhibit the bioconversion of ALA to elongated forms (Elkin et al., 2018; Toomer et al., 2019), and this effect can also occur with CLA supplementation alongside vegetable oils or Vit E (Franczyk-Żarów et al., 2019). This evidence points to competitive interactions among dietary FAs within biological systems, indicating that feed additives are not just about their inclusion level but also about the overall dietary matrix. Further supporting this concept, in dairy goats, a diet rich in high C18:1n9 showed to reduce ALA and its elongated n-3 FAs in milk, whereas LO increased their contents (Martínez Marín et al., 2012), providing direct evidence that dietary C18:1n9 antagonizes ALA bioconversion. Similarly, Jiang et al. (2024) identified genetic factors, and their findings propose that beyond the dietary matrix, host genotype also impacts the result of FA interactions and enrichment efficacy.

The efficacy of FA is likely dependent on the overall FA composition of the diet (their ***interaction***); for example, the literature proposes an antagonistic relationship between C18:1n9 (oleic acid) and ALA; more specifically, high levels of dietary oleic acid may attenuate the ALA absorption rate (Elkin et al., 2018). Furthermore, 18:4n3 (stearidonic acid, an intermediary in the elongation/desaturation route of n-3 PUFA) and C20:4n6 (arachidonic acid, a long-chain n-6 FA competing for the same *FADS1/FADS2* enzymatic pathways) were observed to attenuate the ALA level in egg yolk and chicken liver (Elkin et al., 2018). A similar inverse pattern has also been reported between oleic acid and arachidonic acid in the chicken breast muscle, which is further related to ALA (Høstmark & Haug, 2014). However, these interactions are not yet concrete in the field, although they are rather controversial (Baker et al., 2016; Nogoy et al., 2020), underscoring the need for further investigations.

Regardless of the species, balancing the dietary ***n-6:n-3 ratio*** is very critical, not only in relation to interactions during absorption but also in metabolic pathways. A high ratio (> 10:1) is likely to promote inflammatory signals via enzymatic/non-enzymatic oxidation of n-6 FAs within biological systems (Ali & Szabó, 2023). In practice, grass-fed ruminants naturally maintain a low n-6:n-3 ratio, whereas monogastric diets (grain-heavy and low-fibre) benefit from n-3 PUFA supplementation to offset pro-inflammatory imbalances. Attia et al. (2022) refined the ratio for layers, showing that while a 9.3:1 n-6:n-3 ratio optimizes laying performance, a lower 5.5:1 ratio is required to maximize n-3 enrichment and boost the hens' immune response. Alterations within the dietary n-6:n-3 ratio likely affect cytokines and immunological responses, as demonstrated in broiler chickens (Ibrahim et al., 2018). Hence, these points highlight the complexity of oil addition during diet formulation to ensure efficient nutrient utilization, enhance animal health, and produce high-quality products.

##### 3.4.3.4 Excessive supplementation risk

A critical remark related to ruminants is that ***excessive dietary oil/fat*** (> 5–10% DM) in dairy animals elevates the risk of milk fat depression (MFD) and disrupts fibre digestion (e.g., neutral detergent fibre) and the ruminal microbiota profile (Palmquist & Jenkins, 2017; Rodrigues et al., 2019) and, consequently, the fermentation process. As explicitly demonstrated by He and Armentano (2011), feeding unprotected oils rich in LA or ALA markedly decreases milk fat concentration and yield, whereas PO does not, indicating that the degree of unsaturation dictates the severity of MFD. In agreement, the findings of Razzaghi et al. (2022) indicate that MFD risk is not just a consequence of oil unsaturation but likewise dose-dependent and context-specific. In addition, different dietary oils (LO, RO, and SO) differently modulated ruminal fermentation, referring to that oil type-specific ruminal effects must be considered when setting upper inclusion limits. These challenges represent important directions for future studies employing ruminant model. For non-ruminants, excessive oil (typically >5% of the diet in poultry and >10% in swine) accelerates digesta transit, reducing macro/micronutrient bioavailability and animal productivity (Neijat et al., 2016), especially at earlier stages of life due to immature pancreatic lipase secretion, low bile acid pool size and limited intestinal absorptive surface area (Hagey et al., 2010). Furthermore, high FO supplementation can directly affect performance and health, as supported by de la Fuente-Vázquez (2014) in lambs fed 3.5%, as well as Santoso et al. (2017) and Saleh et al. (2013) in chicken layers fed > 4%. In addition, in broilers fed high-fat starting diets, fat-induced slowing of gastric emptying (through cholecystokinin release) might paradoxically lower feed intake and growth rate. While soluble fibre may slow transit through increased intestinal viscosity and form gel-like substances, it reduces fat absorption and bile acid re-absorption efficacy in the terminal ileum, potentially inducing a hypocholesterolemic effect (Haider & Wilde, 2020) and reducing fat-soluble vitamin absorption. Oil inclusion levels should therefore be titrated in accordance with developmental stage, with larger levels allowed in finisher phases when digestive capacity is fully developed and lower limits applied during starter phases.

##### 3.4.3.5 Dietary oil section short summary

Lipid biofortification strategies are applicable, leading to favourable FA profiles and health attributes; however, it is necessary to distinguish between ALA deposition (via plant oils) and true EPA/DHA enrichment (which requires marine sources). Monogastrics efficiently deposit dietary PUFAs, whereas ruminants generally require rumen-protected forms for efficient enrichment, although partial fortification with unprotected forms has been documented. It is highly recommended to scale dietary oil supplementation with antioxidants (e.g., Vit E); otherwise, unpleasant organoleptic properties can develop. In furtherance of a lack of knowledge on the balanced scaling of antioxidant requirements at high PUFA inclusion levels, the main gap is the lack of scalable, ecologically viable, and sensory-neutral alternatives to FO for DHA enrichment. Though MO (do not alter sensorial qualities in meat and eggs) appears as an optimal replacer for FO, its commercial viability remains constrained by high production costs and the need for comprehensive life cycle assessments. Dietary lipid interactions likely complicate formulation, in which strategies based on plant-marine oil optimize n-6:n-3 ratio, but potential antagonistic interactions can deteriorate the final product quality. Excessive oil supplementation is not recommended, as it can disrupt ruminal fermentation and cause MFD in ruminants while reducing intake and suppressing growth of monogastric animals.

## 4 Development of a framework for precision enrichment

**Fig. 11:**
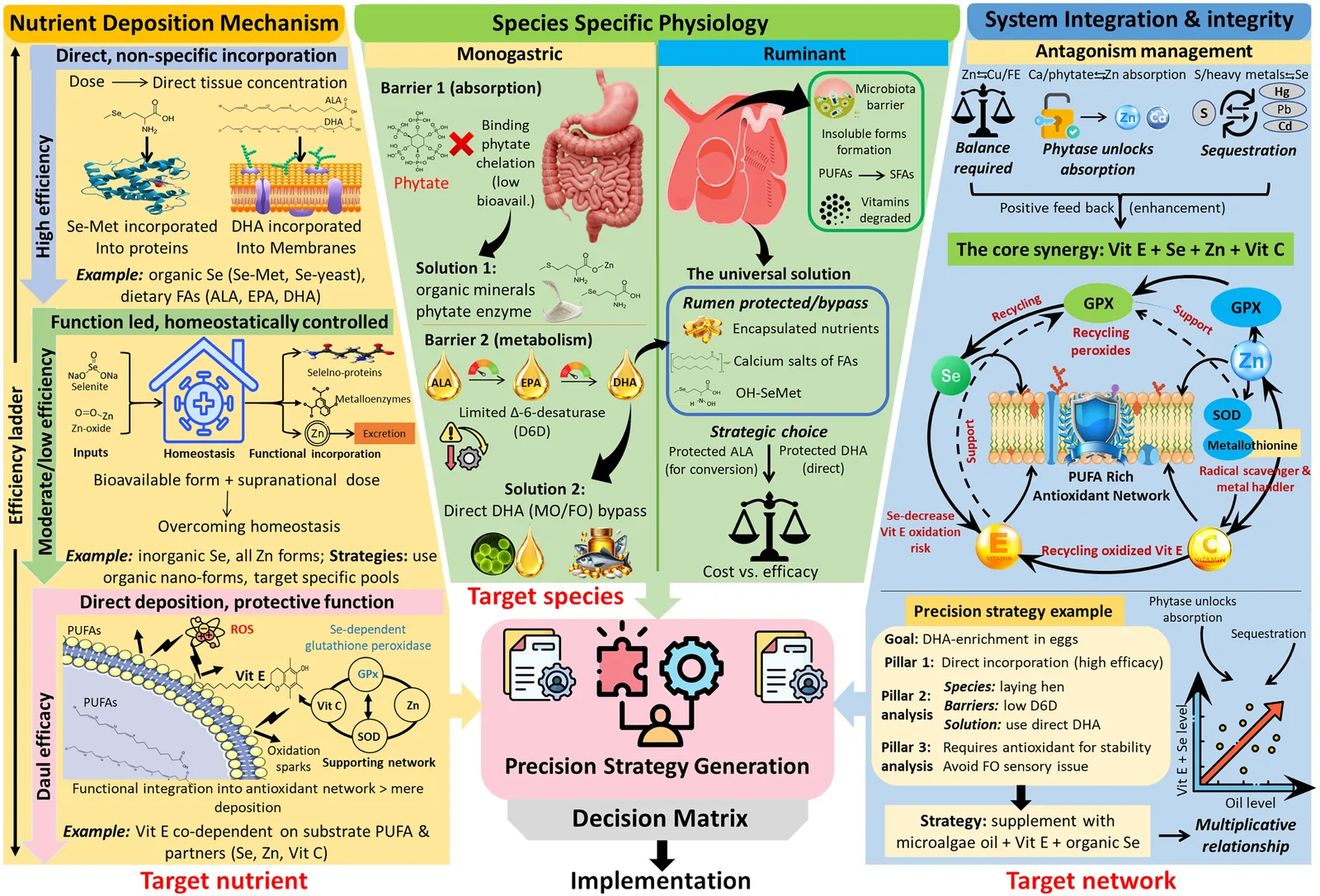
A schematic representation of the framework for precision enrichment of animal products.

The key step in most enrichment approaches is identifying the metabolic pathway mediating nutrient deposition, as this strongly influences the enrichment efficacy. According to the aforementioned sections, fortifying animal products through nutritional strategies is highly challenging, requiring comprehensive knowledge beyond the simple nutrient addition. In this respect, the efficacy of Se, Zn, Vit E and dietary oils is liable to factors such as the complex relationship between chemical form, digestive physiology, metabolic pathways, and nutrient interactions. As an illustration, targeting mineral enrichment by simply using high doses of an inorganic mineral is most probably associated with antagonisms, toxicity, and/or environmental damage more than proportional tissue enrichment (EFSA, 2014; Hejna et al., 2018). Therefore, rather than just listing impacts, this review suggests a coherent framework for "precision enrichment". Within this context, three interconnected pillars greatly contribute to the development of this framework, making it rationally effective and sustainable. These three pillars are concisely discussed below (extracted from the above sections). Moreover, Fig. 11 illustratively summarises the points and practical solutions of these pillars.

### 4.1 Pillar 1: the nutrient-specific mechanism of deposition

The process through which a nutrient is integrated into animal tissue fundamentally dictates the approach necessary for its enrichment. This efficacy is not uniform across animal species; rather, it operates on a spectrum dependent on whether a nutrient is actively homeostatically regulated or passively reservoir-sequestered (Cousins, 2010; Labunskyy et al., 2014). Misalignment between the supplementation strategy and the underlying deposition mechanism invariably results in wasted resources, metabolic stress, and environmental excretion without achieving the desired product value (EFSA, 2014). This principle is well illustrated by the contrasting behaviours of Zn and organic Se: meanwhile Zn is tightly controlled by metallothionein-mediated homeostasis, limiting dose-response tissue enrichment (as discussed in Section 3.2), organic Se bypasses this regulation through non-specific incorporation, enabling predictable, linear enrichment. Understanding this mechanistic dichotomy is the foundation of any precision approach. Accordingly, categorizing nutrients by their “deposition efficiency” provides a predictive blueprint for formulation. Based on the preceding analysis of additives, a categorization can be established, such as the following:

#### 4.1.1 High efficiency: direct, non-specific incorporation

Within this category, nutrients typically bypass rigorous systemic homeostatic regulation, enabling nutrient concentrations within tissues to rise linearly with bioavailable dietary intake. Organic Se (e.g., SeMet) and dietary FAs are a few fitting examples within this category. As an illustration, SeMet, which is distinct from methionine in the metabolic pathways of cells, exploits the non-specific amino acid transporter B⁰/SLC1A5 and is randomly substituted for methionine during protein synthesis, thereby forming an enormous non-specific systemic reservoir in muscle, milk, and egg albumen (Fairweather-Tait et al., 2010; Labunskyy et al., 2014). This non-selective pathway enables SeMet to elevate tissue Se concentrations 2.5- to 3-fold more efficiently than inorganic sources, which are restricted by the UGA-recoding selenoprotein synthesis machinery (Briens et al., 2014). Recent evidence further supports this efficiency, wherein Se boosted oxidative stability in a linear dose-dependant manner (Zhang et al., 2025b), confirming that this relationship is inherent to non-specific incorporation. In a similar pattern, dietary FAs can be directly incorporated into membranes (phospholipids) and/or adipose tissues (triglycerides), depending on the dietary lipid profile (Bionaz et al., 2020) and the animal metabolic state. This direct deposition pathway is particularly relevant for n-3 PUFAs, as the tissue n-3 FA concentration in monogastrics responds dose-dependently to dietary inclusion, for example, as demonstrated in hen eggs (Yalçin & Ünal, 2010). However, it is important to note that for ruminants, this process is generally more effective when lipid protection is employed before absorption and incorporation, considering the rumen biohydrogenation process transforms unprotected PUFAs to SFAs (Yakubu et al., 2023), negating the direct incorporation advantage. Moreover, this ruminal modification is not limited to lipids, as rumen microbes can degrade unprotected organic Se to yield insoluble selenides (Hachemi et al., 2023; Hachemi & Sherlock, 2024). These points provide a strong mechanistic basis for the consistent enrichment efficacy of organic Se and dietary oils observed across species, as systematically discussed in 3.1.2, 3.4.2.

#### 4.1.2 Moderate to low efficiency: function-led, homeostatically controlled incorporation

This category comprises nutrients whose tissue concentrations are actively defended by physiological set points, making brute-force supplementation extremely inefficient. Examples of this category are inorganic Se and all forms of Zn, due to their metabolic conversion requirement and strong homeostatic regulation rate. For instance, inorganic selenite and selenate are primarily employed (via bioconversion) for the production of selenoproteins (e.g., glutathione peroxidase) via the SECIS element machinery, which is a process subjected to rigorous homeostatic regulation through which excessive Se is excreted in the form of trimethylselenonium; thus, extensive tissue accumulation is avoided (Labunskyy et al., 2014). Zn, an important component for hundreds of metalloenzymes, represents an even stricter homeostatic challenge, in which its cellular flux is tightly buffered by metallothionine, as well as a systemic state of equilibrium maintained through absorption (DMT1, ZIP4) and largely excretion (Cousins, 2010; Li et al., 2019b). Accordingly, overdosing dietary Zn to force tissue deposition most likely generates competitive antagonism with copper and iron, alongside 90% of the excess being excreted through faeces, posing remarkable ecological risks (Zhang et al., 2020b; EFSA, 2014; Hölzel et al., 2012). In this respect, meaningful enrichment strategies using Se and Zn likely require “overcoming” homeostasis, mainly achieved by using forms with high bioavailability (such as organic and novel forms that exploit alternative absorptive pathways (e.g., PepT1 for amino acid chelates)) to optimize metabolic pathways and facilitate diffusion/deposition into body tissues, by targeting non-homeostatic pools with less rigorous regulation (e.g., egg yolk Vit E-mediated transfer and specific protein fractions), or by exceeding homeostatic thresholds via nano-forms, which carry risks of antagonisms and environmental loading (EFSA, 2014). The practical implication is that the cost-to-benefit ratio of Zn enrichment is unfavourable compared with Se or FAs, and producers must weigh marginal gains against environmental and regulatory constraints.

#### 4.1.3 Dual efficacy: direct deposition, protective function

The prime example for this category is Vit E, which represents a dual-function additive: serving simultaneously as a directly depositable nutrient and an essential metabolic stabilizer (functional antioxidant). Like organic Se and PUFAs (4.1.1, 4.1.2), α-tocopherol is efficiently transferred from diet to lipid-rich tissues and products (muscle phospholipid bilayers, egg yolk and milk fat globules, as extensively documented in Section 3.3.2) in a dose-dependent and measurable manner, conferring direct nutritional value to the consumer-facing product (Zingg, 2007). For instance, in laying hens, increasing dietary α-tocopherol from 10 to 200 mg/kg raised yolk α-tocopherol from 100 to about 500 μg/g (Section 3.3). However, its main enrichment value lies in its functional antioxidant role, operating as the primary lipid-phase radical quencher within membrane phospholipid bilayers. Within this role, it intercepts lipid peroxyl radicals (LOO•) to halt the propagation of lipid peroxidation (Traber & Stevens, 2011), protecting co-deposited PUFAs from peroxidative degradation in both living animal tissues and post-mortem stored animal products. Accordingly, this protective function becomes a "tax" on Vit E deposition: as PUFA levels increase, more α-tocopherol is sacrificially oxidized, reducing the net depositable α-tocopherol available for tissue storage (Section 3.3.3.2). This oxidative "tax" was empirically demonstrated by Tomažin et al. (2013), who reported that in broilers fed diets enriched with LO, α-tocopherol alone was insufficient to prevent lipid oxidation, and the combination of α- and γ-tocopherol provided superior protection, suggesting that the antioxidant component of Vit E efficacy depends on both dose and isomeric composition when the oxidative load is high. Further other considerations are with to highlight isomer efficacy, as Voljč et al. (2011) highlighted that current Vit E recommendations for broilers may be inadequate for n-3 PUFA-enriched diets, as the bioactivity of RRR-α-tocopherol exceeded that of all-rac-α-tocopherol by a factor greater than the conventionally assumed 1.36, further complicating the calculation of the oxidative "tax" in precision models. Furthermore, Vit E effectiveness is co-dependent on the activities and regenerative capacities of supporting systems like the Se-GPx and ascorbate systems (Pečjak et al., 2022; Sies & Stahl, 1995). These points must be taken into consideration during strategy development. Ultimately, a complete precision enrichment strategy involving Vit E must account for multiple dimensions: (1) achieving a target α-tocopherol tissue concentration to confer direct nutritional benefit to the consumer; (2) ensuring that this concentration is stoichiometrically proportional to the PUFA load to fulfil its protective function, which preserves the sensory and oxidative integrity of enriched products (Fritsche & Johnston, 1990); and (3) considering the potential spare and regenerative effects associated with the Se and Vit C supplementation.

### 4.2 Pillar 2: the dominance of species-specific physiology

Across livestock animals, differences in the digestive system and physiology have been well acknowledged. Accordingly, the digestive system of the target animal serves as the chief determinant of the success of the enrichment strategy (defining which nutrient form survives digestion to reach the site of absorption intact, or undergoes transformative modifications before reaching the absorption area), establishing a fundamental species-specific distinction, especially between monogastric animals and ruminants, in which this is particularly relevant for ruminant species, where the rumen microbiome extensively modifies dietary inputs, as discussed below.

The monogastric (swine and poultry) system is characterized by direct access to supplemented nutrients; however, it is subjected to metabolic limitations and immature digestive physiology in early life stages, as extensively discussed in Section 3.4.3.4. Herein, the potential main direct luminal absorption barrier can be the antagonist interactions (e.g., phytate-mediated chelation of divalent minerals like Zn²⁺ and Se as selenite), which can be tackled/avoided via the adoption/development of strategies focused on the enhancement of bioavailability, such as the use of organic minerals (e.g., SeMet and ZnMet do not chelate with phytate), employing phytase enzymes (hydrolyze phytic acid), or acidifying the diet to reduce phytate stability. For oils, the metabolic bottleneck in monogastric animals represents a challenge, wherein limited D6D and *FADS2* activity restrict ALA bioconversion to EPA and DHA in most monogastric species, particularly poultry; consequently, achieving meaningful DHA enrichment generally requires preformed DHA from marine sources like MO and FO (Section 3.4.1.3) (Glencross et al., 2025; Zhu et al., 2014b). However, the extent of this limitation varies across species, genotypes, gender, age and tissue types (Poureslami et al., 2010), with pigs generally exhibiting somewhat greater desaturase activity than poultry. Partial bioconversion does contribute to tissue EPA and DPA pools, and the relative contribution of endogenous conversion versus direct deposition merits further quantification across species. Furthermore, the insufficient endogenous emulsification at high fat loads may limit PUFA absorption (most notable in young animals); therefore, employment of exogenous emulsifiers (e.g., lysolecithin) can enhance lipid digestion and absorption from starter diets. Regarding Vit E, the selection of natural Vit E and ensuring adequate dietary fat content for micellar solubilization are the key bioavailability strategies.

In ruminants, the ruminal ecosystem is a distinguished challenge to strategy effectiveness. This ecosystem is a key transformative chamber that dictates nutrients’ fates, mainly represented in the degradation of unprotected organic compounds (e.g., unprotected vitamins (decreasing bio-availability by 30-50%) and amino acid chelates) and the extensive biohydrogenation of 80-95% UFAs to generate SFAs (primarily C18:0) (Hendawy et al., 2021; Hymøller & Jensen, 2010; Yakubu et al., 2023), thereby negating the enrichment benefit (Section 3.4.1.2). The oil physical form plays a critical role in FA flow, as in the case of extruded linseed, which delivered more non-hydrogenated FAs in dairy cows (Sterk et al., 2012). Similarly, inorganic Se and unprotected SeMet are reduced to insoluble selenides by ruminal microbes, lowering bioavailability (Section 3.1.3.1). Accordingly, effective ruminant enrichment strategies are generally defined by the application of rumen-protected forms or rumen-bypass technologies like encapsulated nutrients, calcium salts of FAs, and formaldehyde-treated protein coatings, along with the rumen-stable form OH-SeMet (Alkan & Murz, 2025). These technologies are generally considered important for achieving meaningful enrichment in ruminant products; however, it should be noted that partial enrichment has also been demonstrated with unprotected forms in certain contexts (e.g., low-level FO supplementation increasing milk CLA and n-3 FAs; Kupczyński et al., 2011), albeit with considerably lower efficiency compared with protected formulations. Furthermore, the decision to enrich with ALA (sourced from protected LO) or directly with EPA/DHA (derived from protected FO or MO) must involve a cost-benefit assessment between offering a substrate for limited endogenous conversion and supplying the costly end product directly. For Zn and Vit E, although they are less susceptible to ruminal degradation, their absorption efficiency is lower in ruminants due to complex formation with feed particles and microbial competition, respectively.

### 4.3 Pillar 3: the importance of system integration and synergy

Nutrients do not function independently; as an illustration, synergistic and antagonistic interactions affect their bioavailability, metabolism, and deposition efficacy. Thus, precision enrichment rejects the concept of a single additive; rather, it favours and necessitates the development of complementary nutrient systems that operate synergistically (Rooke et al., 2004). For instance, as n-3 FA enrichment is one of the main goals, the strategy must concurrently fortify the animal's antioxidant defence network. A dietary strategy that neglects these interactions most possibly leads to product degradation, metabolic imbalances and economic losses (Bernardi et al., 2022; Cousins & Liuzzi, 2018). Based on this fundamental principle, several framework-based interactions have been identified:

#### 4.3.1 The antioxidant synergetic network

This network type is the most critical interaction matrix for lipid-enriched functional foods, representing the core synergism. Fortification of animal products with PUFAs likely elevates the risk of oxidative vulnerability of the product, as these highly unsaturated molecules are prone to peroxidation. Therefore, the integration of an antioxidant network (Section 3.3.3.2) like Vit E (the primary lipid-soluble radical quencher), Vit C (mediates Vit E regeneration at the membrane-water interface), Se (for glutathione peroxidase to reduce hydroperoxides that compromise Vit E), and Zn (for superoxide dismutase and metallothionein) is not mandatory but practically recommended to preserve the functional value of the enriched product. Practical evidence underscores this synergy across species: in rabbit meat, despite that Se-yeast did not mitigate the pro-oxidant effect of a 4% dietary FO, Se+ Vit E combinations consistently preserve oxidative stability in PUFA-enriched pork and broiler meat (Bernardi et al., 2022; Danuta et al., 2020; Jiang et al., 2017); in broiler meat, the combination of Vit E+ Se+ Vit C significantly reduced TBARS compared with Vit E alone (Leskovec et al., 2019); in eggs, Se-yeast combined with Vit E extended shelf life by maintaining α-tocopherol stability (Zou et al., 2025); and in dairy cows, the addition of Vit E to n-3 FA-enriched diets protected milk from spontaneous oxidation (Rico et al., 2021). These supplementary components operate as a cohesive unit rather than isolated inputs, ultimately preserving UFAs in tissues. Hence, formulating these nutrients in isolation undermines the efficacy and economic efficiency of the entire strategy.

#### 4.3.2 Strategic antagonism management

Rationally, a successful synergistic strategy must be balanced with mitigation of competitive antagonism, driven by shared metabolic pathways. Across the review, several examples have been delivered, for example: (1) High dietary Zn levels must be balanced with copper and iron (Cousins & Liuzzi, 2018) (Section 3.2.3.1); (2) High concentrations of sulfur or heavy metals can sequester Se for the Na-dependent neutral amino acid transporter (B⁰ system) in the intestine, while, in ruminants, the ruminal sulfate-reducing bacteria transform excess sulfate to a reactive form that binds to Se and forms insoluble complexes (Arshad et al., 2021; Wolffram et al., 1989) (Section 3.2.3.1); and (3) Calcium and phytate can inhibit Zn absorption (Philippi et al., 2023). (4) high dietary C18:1n9 or LA can compromise ALA bioconversion (Elkin et al., 2018; Toomer et al., 2019; Ibrahim et al., 2018) (Section 3.4.3.3). These antagonisms are not theoretical; they have practical consequences and can reach immunomodulatory effects. Therefore, the proposed concept of precision formulation must comprehensively evaluate the entire supplementation program to control competitive interactions (e.g., Zn:copper:iron, sulfur:Se) and adjust inclusion levels, chemical forms (protected forms or enzymes such as phytase) and time of supplementation to minimize interference. However, as the literature reveals gaps in certain areas, practical implementation requires further validation across diverse commercial production systems, aiming to maximize both bioavailability and functionality.

#### 4.3.3 Relationship between oils and antioxidants

The relationship between dietary PUFA content and antioxidant requirement is stoichiometric (multiplicative in nature) rather than merely additive, pointing out a proportional relationship; specifically, the requirement of dietary Vit E (or other additives with antioxidant properties) increases linearly with the type of PUFAs and their concentrations. This principle has been clearly demonstrated in n-3-enriched eggs (Zou et al., 2025) and PUFA-rich milk (Rico et al., 2021). As a practical formulation guideline, Fritsche and Johnston (1990) proposed that at least 10 mg α-tocopherol should accompany each additional gram of dietary n-3 PUFA to maintain tissue oxidative stability; thus, it provides an actionable minimum threshold for precision enrichment formulations. Of note, this ratio establishment is based on limited experimental conditions; consequently, it likely requires adjustment according to several factors, comprising the specific PUFA species (e.g., DHA, with 6 double bonds, is substantially more peroxidizable than ALA, with 3 double bonds), the target animal species and its endogenous antioxidant capacity, the presence of other components of the antioxidant network (e.g., Se-dependent glutathione peroxidase, Vit C), and the intended storage conditions and shelf-life of the final product. Accordingly, rather than being a set universal requirement, this recommendation should be viewed as a baseline. The consequences of inadequate antioxidant provision relative to PUFA load can be severe; for example, yolk malondialdehyde levels quadratically increase with LO inclusion, and rabbit loins fed LO without Vit E scaling exhibit significantly elevated TBARS during storage (Dal Bosco et al., 2018; Lee et al., 2021). Herein, in order to optimize the ratio for practical feed formulation, validation studies specific to species and products are required.

### 4.4 Practical applications of the precision enrichment-decision matrix

The previously described triplicate framework is a valuable tool that can be employed as a decision matrix for producers and nutritionists, enhancing fortification efficiency and aspects related to the environment, sustainability, and economy. As a result, the focus shifts from the overall output “what works” to the optimized outcome “why it works and how to optimize it”, consequently transforming the development of functional animal products into a foreseeable science instead of an empirical art. Accordingly, the following questions are employed to formulate strategies for enrichment targets: (1) Mechanism: what is the deposition pathway for the nutrient I am targeting? (2) Physiology: what are the barriers that are specific to each species? (3) Integration: what synergies or antagonisms require careful management?

**Fig. 12:**
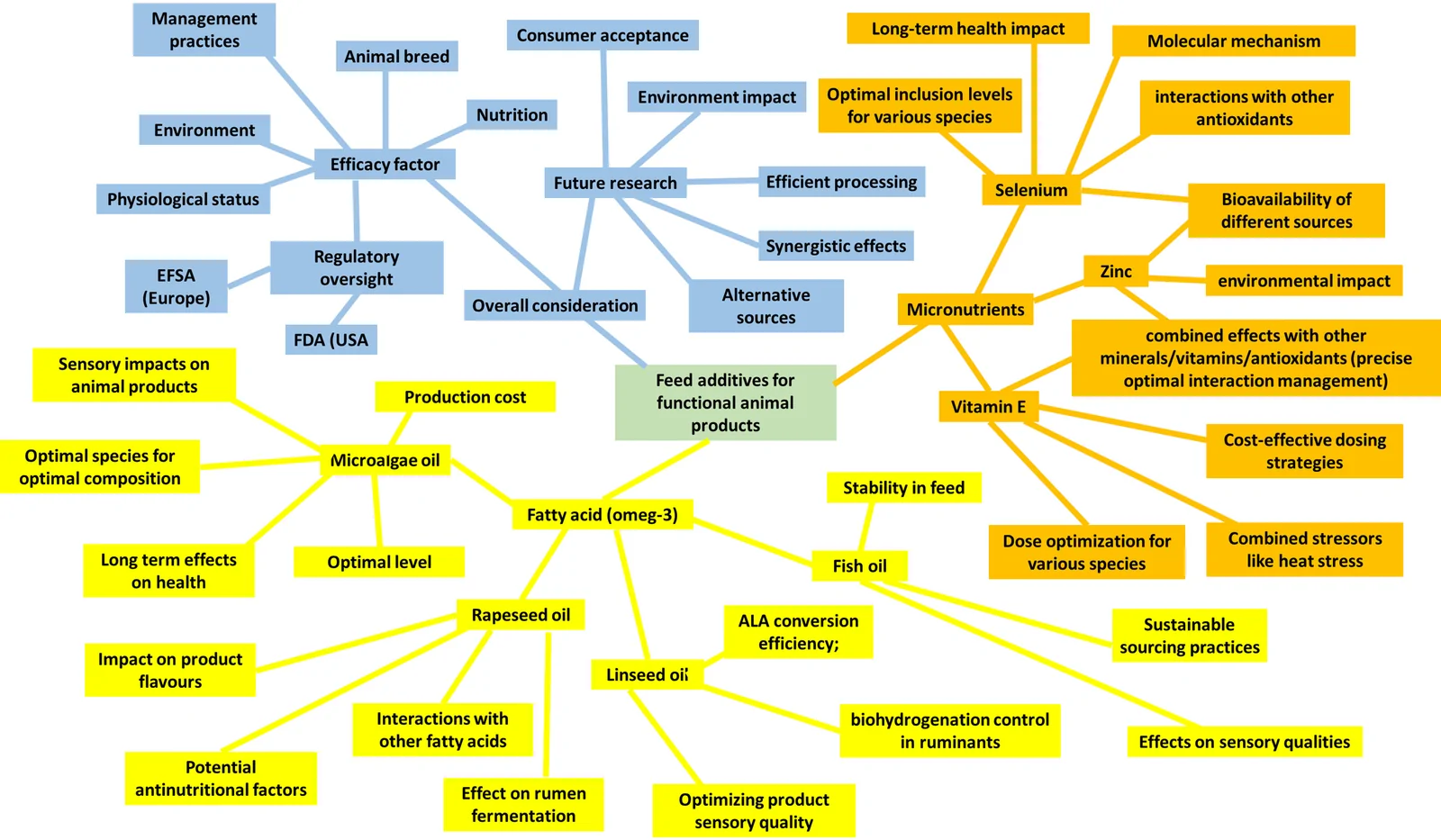
Key components of different feed additives for functional animal products, highlighting the existing knowledge gaps related to each specific dietary supplement.

As an illustration, a target to "increase DHA in milk" requires querying the matrix: (1) Mechanism: DHA relies on direct incorporation, requiring preformed DHA sources; (2) Physiology: rumen-protected marine oils are generally recommended to reduce biohydrogenation losses in ruminants (Tóth et al., 2019); (3) Integration: DHA demands co-supplementation with rumen-protected Vit E and Se to prevent milk fat oxidation and off-flavours (Bragaglio et al., 2015), as well as select MO over FO to enhance sensory compatibility. On the contrary, targeting "increase ALA in broiler meat" yields a distinct matrix: (1) Mechanism: direct deposition of ALA is efficient and economically viable through plant oils, especially LO; (2) Physiology: monogastric absorption is direct, but D6D and *FADS2* limitations limit DHA bio-production (Glencross et al., 2025); (3) Integration: Vit E must be scaled to the LO dose to prevent lipid peroxidation (Bernardi et al., 2022). Through mapping these 3 pillars, nutritionists can anticipate efficacy as well as avoid biological barriers. However, it should be noted that this proposed precision framework represents a conceptual model derived from current evidence; its practical implementation at a commercial scale requires ongoing validation and adjustment. Accordingly, several gaps are yet noticeable (see Fig. 12), referring to the need for more pillars that may emerge in the future, which is a typical aspect of advancements in technologies and research.

### 4.5 Technological advancements and a precision enrichment-decision matrix

Precision livestock farming (PLF) technologies are rapidly facilitating the commercial use of precision enrichment. The real-time monitoring technologies, such as near-infrared spectroscopy for online feed quality assessment, wearable biosensors for metabolic status monitoring, automated milk composition analyzers (e.g., MFD or oxidation markers), inline egg quality sensors, and AI-assisted diet optimization algorithms, are of great help. For instance, the employment of these technological advancements offers tremendous opportunities and possibilities to dynamically re-adjust antioxidant and PUFA supplementation strategies, as well as their ratios, in response to individual animal variation, production stage, environmental stressors and feedstuff batch composition. Moreover, nano-encapsulation and microencapsulation technologies are improving the delivery of bioactive nutrients (e.g., rumen-protected FO, and Nano-Se), enhancing rumen bypass and intestinal bioavailability. Through these technological advancements, individualized enrichment treatments would replace population-average enrichment protocols, which are typically static in the field. In addition, these advancements play crucial roles in the development of responsive tactics that minimize both under-supplementation (which limits product enrichment) and over-supplementation (which increases cost, toxicity risk, and environmental burden) alongside its related ecological consequences (Se and Zn excretion and n-3 PUFA-derived off-flavours), while maximizing functional food output per unit of supplement input. However, to attain this vision, animal nutritionists, computational scientists, sensor engineers, and environmental life cycle analyzers must collaborate together across disciplines, which is a crucial strategic path for functional animal product development in the upcoming ten years.

### 4.6 Economic and commercial considerations for functional feed supplementation

Though numerous studies confirm the enrichment of animal foods via dietary-based strategies, several concerns, such as economic feasibility and logistic field applicability, are equally important for practical adoption. need further investigations and validations. Among these confrontations, at the feed formulation level, the main challenge presented in the cost differential between additives’ forms is of great interest. Organic (like chelated SeMet and ZnMet) and novel forms (e.g., Nano-Se, Se yeast and encapsulated/coated lipids) typically have an initial high cost per ton of feed compared with inorganic salts (such as NaSe and ZnO) and unprotected oils (Cai et al., 2021; Surai & Fisinin, 2016), thereby must be offset by the market value of the enriched product. However, a strict cost-per-ton study is unlikely to capture the complete economic picture; thus, a broader view with various aspects is needed, including, importantly, the perspective of return-on-investment (ROI) basis precision, considering the premium pricing of functional foods (e.g., Se-enriched eggs or DHA-fortified milk/meat) in markets where consumers are health-conscious, market demand, and regulatory compliance costs.

Over and above that, farm-level logistics must be considered with respect to field applicability. For example, high doses of Zn or unprotected oils can damage feed mill equipment, compromise feed palatability (consequently reducing feed intake), increase both handling and storage costs, or provoke digestive upsets, negating theoretical enrichment positive effects. In this regard, the stability of supplemented nutrients during feed processing (pelleting and extrusion) and storage must be verified, as losses can undermine the intended enrichment. Moreover, the transition from inorganic to organic minerals frequently facilitates lower inclusion rates to provide equivalent or greater tissue deposition (Briens et al., 2014; EFSA, 2014), which can offset the higher unit cost along with minimizing trace mineral excretion into the environment (mitigating ecological footprint) (Hölzel et al., 2012), which is a present-day sustainability and regulatory concern. In addition, targeted nutritional synergies are crucial for economic optimization; for example, the dietary combination of Vit E, Vit C and Se creates a regenerative antioxidant network that boosts oxidative stability far more effectively than high doses of Vit E alone, possibly minimizing the high cost related to the use of expensive natural RRR-α-tocopherols at high inclusion levels (Leal et al., 2019; Pečjak et al., 2022). In addition, Vit E with probiotics showed to improve broiler meat qualities more than either supplement alone; therefore, multi-functional feed additive blends may offer a more cost-efficient approach by simultaneously addressing enrichment, oxidative stability, and product quality in a single formulation.

The high production cost and volatile supply chains continue to limit the use of innovative lipid sources, especially those driven from microalgae. Herein, FO is relatively cheaper than MO; however, it impairs sensorial attributes (fishy off-flavours) and, consequently, compromises consumer acceptance and product marketability, lessening the possibility for premium pricing (Feng et al., 2020; Van Wyngaard et al., 2023). In contrast, MO preserves sensory quality, but its high manufacturing costs presently limit its practical application to high-end (Shah et al., 2018). In this respect, the development of cost-effective heterotrophic fermentation technologies and the exploration of alternative marine sources (e.g., krill oil, calanus oil or fish by-products) may gradually reduce costs. However, until large-scale algae cultivation significantly reduces costs, the commercial use of MO may remain confined to high-end product lines. Likewise, in ruminants, the economic feasibility of DHA-enriched ruminant products will remain a key challenge, and strategies based on plant oil n-3 (ALA) enrichment may represent a more accessible intermediate goal. However, the adoption of encapsulated additives (to prevent biohydrogenation and MFD) is largely recommended; accordingly, protected fats are, to some extent, a necessary capital expenditure for successful dairy enrichment (He & Armentano, 2011). Eventually, in order for precision nutrition to progress from experimental trials to broad industry adoption, nutritionists must balance the biological effectiveness of novel dietary additives against feed conversion ratios, animal health indices, market price premiums for functional claims, and environmental compliance expenditures.

## 5 Conclusion

Based on the literature reviewed over the past fifteen years, strategic dietary manipulation is a ***valuable and attainable approach*** for developing functional animal-derived foods that meet evolving consumer demands for health-promoting foods. From the reviewed literature, to enhance the functional antioxidant levels of meat, milk, and eggs, Se and Zn are among the most widely used microelements, mostly in the following forms: NaSeIII, SeMet, Se-yeast, Nano-Se, ZnMet, ZnSO_4_·7H_2_O, and Zn-Pal. In the majority of the reviewed studies, a ***hierarchy*** across supplement forms is likely demonstrated, in which the efficacy of organic and nano forms of Se and Zn consistently outperforms traditional inorganic forms (like NaSe and ZnO), mainly attributable to their higher bioavailability and metabolic integration; however, the evidence base is heterogeneous, and certain studies have reported meaningful enrichment with inorganic forms. Crucially, the selection of inorganic forms may carry some risk by essence under high dietary PUFA conditions, mainly through acting like pro-oxidant; thus, the quality of a product might be accidentally compromised by choosing the improper mineral form. D-α-tocopherol (Vit E) is the most widely used feed supplement for improving animal product value. Among its forms, the natural forms exhibited greater biopotency than did synthetic analogues. Critically, each supplement’s ***efficacy*** is intricately tied to the nutrient chemical form, species-specific digestive physiology, dietary interactions, and environmental sustainability. The evidence reviewed in this work further underscores that these principles are not merely theoretical but are supported by empirical evidence across diverse production systems, from broiler chickens fed LO and Se-yeast to dairy cows supplemented with rumen-protected FO and Vit E, consistently suggesting that mechanistic alignment between nutrient form and metabolic pathway is one of the most important determinant of enrichment success. Routinely, Vit E is co-supplemented with Se and/or Vit C in animal feed for efficient animal product development, a strategy supported by the antioxidant regeneration network described in 3.3, 4.3.3. Nevertheless, Vit E biological value is also influenced by factors similar to those affecting Se/Zn efficacy. Notably, the synergistic ***interaction*** between nutrients, especially between Vit E and Se, is of particular scientific and practical importance. Their interaction serves as a useful tool for optimizing the antioxidant defence systems in both animals and their obtained products, and represents a central principle of the precision enrichment framework proposed in this review. In this respect, it is important to emphasize that while preclinical and epidemiological data are promising, robust human intervention trials are necessary to validate the health benefits associated with these enriched animal-derived foods, which would help narrow the gap between animal nutrition research and evidence-based public health recommendations.

To ensure the development of omega-3 (n-3) PUFA-rich animal products, LO, RO, FO and MO of nutritional importance have been used in recent years. This review underscores the ***distinction*** between monogastric animals and ruminants; swine and poultry efficiently deposit dietary PUFAs, whereas ruminants benefit more from rumen-protected lipids to bypass microbial biohydrogenation, even if there has been evidence of partial enrichment with unprotected forms. Likewise, lipid enrichment strategies in ruminants can align with climate goals, as LO has shown efficacy in reducing methane emissions. In addition, MO appears to be among the most effective single sources for DHA enrichment across species, while LO provides a cost-effective alternative for ALA deposition. The ***combination*** of plant-based oils with marine-based oils has shown efficacy in enriching a broad spectrum of n-3 PUFAs, enhancing the contents of ALA, EPA, DPA and DHA. However, n-3 PUFA enrichment, although beneficial for human health, introduces challenges such as ***oxidative instability*** and, in the case of FO, sensory trade-offs. The parallel addition of antioxidants (especially Vit E scaled to the PUFA load) or encapsulation is highly advisable for maintaining product quality, shelf life and sensorial characteristics.

From the above, the development of functional animal-derived foods is best supported by a ***precision nutrition framework***, which integrates multiple additive dimensions simultaneously in a coherent system, rather than relying on a strict single additive strategy. Such an approach necessitates a thorough understanding of (1) nutrient-specific deposition mechanisms, (2) species-specific physiology, and (3) system integration and synergy to provide a rational and predictive structure for designing enrichment programs. Furthermore, these strategies should comply with the regulated/recommended levels, cost-effectiveness and environmental aspects. ***Technological advancements*** can also be integrated within the concept of a precision nutrition approach. Precision livestock farming, using real-time sensors and AI, enables dynamic, individualized antioxidant/PUFA supplementation to improve functional outputs while reducing waste and environmental impact.

It should be noted that even though preclinical and epidemiological studies considerably support the claimed medical benefits of these improved products, long-term human intervention trials remain necessary to provide solid validation. In a similar manner, it is important to maintain long-term monitoring of animal health and environmental impacts of novel forms, as well as proposed high-dose strategies, before broad commercial adoption can be recommended.

## 6 Research gaps and future research

It is well known that animal nutrition and functional food research areas have been rapidly progressing within recent decades; however, some topics are not well comprehended and merit further investigations. With respect to the review topic, future research should focus on comprehensive ***comparative*** studies between inorganic and organic Zn/Se sources, as well as novel forms like Nano-Zn/Se and Se-enriched probiotics. These studies should evaluate not only the deposition in animal products but also the bioavailability thresholds, cost-effectiveness, interaction with other dietary cofactors (e.g., high phytate and fibre contents), and long-term impacts on animal health. In addition, it would be valuable to explore ***new innovations*** like Nano-Se and chelated Zn technologies for enhanced stability and reduced ecological impact, particularly in monogastric and ruminant systems. It is also recommended that new and innovative feed supplements that combine multiple beneficial nutrients be investigated, making it easier to achieve the desired nutritional outcomes in various animal-derived foods. Among the identified gaps, the need for exact stoichiometric models defining the antioxidant-to-PUFA scaling ratios (Pillar 3) is particularly pressing; therefore, future studies should empirically determine the minimum Vit E and Se required per gram of specific PUFAs (DHA *vs*. ALA) to help ensure shelf-life stability across different animal products, species, and storage conditions. However, strategies based on combined methods would benefit from a holistic understanding of ***interactions*** in both digestion and metabolism. Again, the ***environmental*** impact of Se and Zn supplementation, especially from innovative sources such as Nano-Se, should be evaluated via life cycle assessment (LCA) alongside the economic feasibility of adopting such strategies in large-scale animal production systems.

The intricate relationship between animal health and the ***gastrointestinal microbiome*** system is very significant, which signifies the research shift towards dietary strategies for manipulating the microbiome profile as a primary tool for functional food development. Future studies with these innovative focuses may aid in reducing the antibiotic load in animal farming, accelerating the global regulatory harmonization movement, improving product qualities, and decreasing the ecological footprint. Moreover, elucidating nutrient-microbiome axes through integrated multi-omics approaches, combining metagenomics (community structure), metatranscriptomics (functional gene expression) and metabolomics (luminal metabolite profiles) in well-controlled livestock feeding studies, would substantially advance mechanistic understanding of enrichment strategies.

Researchers should also look into the ***long-term*** effects of adding oils to animal diets on their health, reproductive performance, and product quality (both nutritive and sensorial), with a focus on the ***sensory attributes*** (odour, taste, and texture) and the shelf life of meat, milk, and eggs that have been enriched with PUFAs. A specific gap remains in defining the safe upper limits of marine oil inclusion, aiming at avoiding excessive supplementation. Within this perspective, integrating ***AI-driven formulation tools*** to dynamically adjust n-6:n-3 ratios, antioxidant levels, and mineral balances on the basis of real-time feed quality and animal health data could be highly beneficial. It is also important to assess the use of various oil supplements in animal feed and their ***environmental footprint*** by employing tools like life cycle assessment (LCA) and considering factors such as land use efficiency (particularly for LO and RO), resource input for algal cultivation, and potential changes in greenhouse gas emissions, particularly in ruminants.

While linseed, rapeseed, fish, and microalgae oils have been extensively studied, future research should explore the potential of other sustainable oil sources, such as insect-derived oils or oils from underutilized crops, to enhance FA profiles in animal products. To achieve the best outputs of this approach, ***multidisciplinary collaboration*** (e.g., nutritionists, microbiologists, and environmental scientists) is important to address gaps in nutrient-gut-environment interactions. A key priority remains conducting robust ***human clinical trials*** to support the purported health benefits of consuming these enriched animal-derived foods.

## Ethical statement

The current manuscript does not involve human or animal models; therefore, ethical approval is not available. Moreover, none of the data presented within the manuscript necessitates permission of approval.

## AI-generative data declaration

The authors did not use any artificial intelligence assisted technologies in the writing process, except for English language proofreading.

## Financial support statement

This work was partially funded by the Hungarian Academy of Sciences (HUN-REN-MATE, Mycotoxins in the Food Chain Research Group) and by the Hungarian National Laboratory project RRF-2.3.1-21-2022-00007, and further by the Research Excellence Program 2026 of the Hungarian University of Agriculture and Life Sciences.

## CRediT authorship contribution statement

**Omeralfaroug Ali:** Writing – review & editing, Writing – original draft, Visualization, Validation, Supervision, Software, Resources, Project administration, Methodology, Investigation, Funding acquisition, Formal analysis, Data curation, Conceptualization. **Moses Teye:** Writing – review & editing, Validation, Methodology, Investigation. **Tamás Tóth:** Writing – review & editing, Writing – original draft, Validation, Methodology, Investigation, Formal analysis, Data curation. **George Bazar:** Writing – review & editing, Validation, Methodology, Investigation, Conceptualization. **András Szabó:** Writing – review & editing, Validation, Methodology, Investigation, Funding acquisition, Formal analysis. **Richard Badu:** Writing – review & editing, Validation, Investigation. **Mohamed Maki:** Writing – review & editing, Validation. **Haruna Gado Yakubu:** Writing – review & editing, Writing – original draft, Visualization, Validation, Supervision, Methodology, Investigation, Formal analysis, Data curation, Conceptualization.

## Declaration of competing interest

The authors declare that they have no known competing financial interests or personal relationships that could have appeared to influence the work reported in this paper.
